# Reconstitution of a Minimal Ribosome-Associated Ubiquitination Pathway with Purified Factors

**DOI:** 10.1016/j.molcel.2014.07.006

**Published:** 2014-09-18

**Authors:** Sichen Shao, Ramanujan S. Hegde

**Affiliations:** 1 MRC Laboratory of Molecular Biology, Cambridge, CB2 0QH, UK

## Abstract

Ribosomes stalled on aberrant mRNAs engage quality control mechanisms that degrade the partially translated nascent polypeptide. Ubiquitination of the nascent protein is mediated by the E3 ligase Listerin via a mechanism involving ribosome subunit dissociation. Here, we reconstitute ribosome-associated ubiquitination with purified factors to define the minimal components and essential steps in this process. We find that the primary role of the ribosome splitting factors Hbs1, Pelota, and ABCE1 is to permit Listerin access to the nascent chain. Listerin alone can discriminate 60S- from 80S-nascent chain complexes to selectively ubiquitinate the former. Splitting factors can be bypassed by artificially removing the 40S subunit, suggesting that mere steric hindrance impedes Listerin recruitment. This was illustrated by a cryo-EM reconstruction of the 60S-Listerin complex that identifies a binding interface that clashes with the 40S ribosomal subunit. These results reveal the mechanistic logic of the core steps in a ribosome-associated quality control pathway.

## Highlights

• Stalled ribosome-nascent chain ubiquitination is reconstituted with purified factors
• Ribosome splitting is required for ubiquitination machinery to access nascent chains
• Listerin E3 ligase has the capacity to discriminate 60S subunits from 80S ribosomes
• Ribosome-Listerin structure reveals binding is mutually exclusive with 40S subunit

## Graphical Abstract

**Figure:**
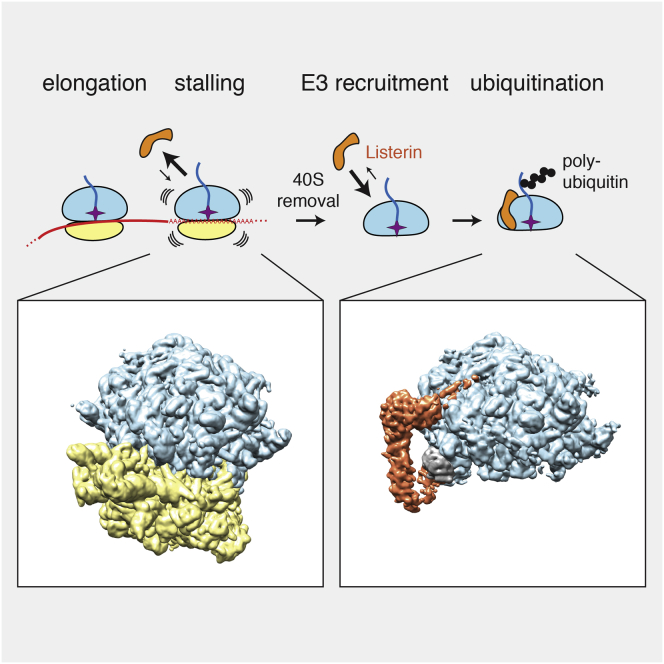

## Teaser

Ribosomes that stall during protein synthesis selectively recruit the ubiquitin ligase Listerin to tag the nascent polypeptide for degradation. Shao and Hegde have reconstituted stalled ribosome-associated ubiquitination to reveal the minimal required factors, the sequence of events leading to Listerin recruitment, and the structure of the ribosome-Listerin complex.

## Introduction

Cells constantly monitor the quality of their proteins and selectively destroy aberrant species. Failure of quality control can lead to misfolded protein accumulation, an event linked to numerous diseases from diabetes to neurodegeneration (Chiti and Dobson, 2006). Quality control is now appreciated to act at essentially every stage in the life of a protein (Wolff et al., 2014). The defining and decisive event in any quality control pathway is substrate detection and selective targeting for degradation. Delineating the mechanistic basis of these key decisions for each quality control pathway is an important goal.

A newly discovered set of quality control pathways acts at the ribosome on nascent polypeptides that have yet to complete synthesis. Ubiquitinated nascent chains were observed many years ago (Sato et al., 1998), and proof-of-principle experiments established the possibility of cotranslational degradation (Turner and Varshavsky, 2000). However, systematic demonstration of nascent chain ubiquitination on the ribosome has only recently been undertaken (Duttler et al., 2013, Wang et al., 2013), and the pathways are poorly understood. The best defined of these is a ribosome-associated quality control (RQC) pathway that degrades the partially synthesized protein products of translationally stalled ribosomes (Bengtson and Joazeiro, 2010, Brandman et al., 2012, Dimitrova et al., 2009).

This pathway was discovered from the observation in yeast that translation of a “nonstop” mRNA lacking a stop codon generates a protein product degraded by the proteasome (Ito-Harashima et al., 2007). Nascent protein degradation was linked to multiple modes of ribosomal stalling including mRNA truncation, secondary structure within the coding region, and translation of polybasic residues such as reading into the polyA tail (Dimitrova et al., 2009). Reporters for this degradation pathway led to the identification of Ltn1 as the E3 ubiquitin ligase required for nonstop protein ubiquitination at the ribosome (Bengtson and Joazeiro, 2010). Listerin, the mammalian homolog of Ltn1, causes neurodegeneration when mutated in mouse (Chu et al., 2009), highlighting the physiologic importance of RQC.

A combination of genetic and physical interaction studies in yeast identified additional components of this RQC pathway including Asc1, Hel2, Tae2, Rqc1, and Cdc48 (Brandman et al., 2012, Defenouillère et al., 2013). Asc1 and Hel2 appear to act early in the pathway and are needed for stalling of ribosomes at codons of polybasic residues (Brandman et al., 2012, Kuroha et al., 2010). Once stalled, a poorly understood set of events lead to nascent chain ubiquitination. Affinity purification of tagged Ltn1 copurified the 60S ribosomal subunit, Tae2, Rqc1, Cdc48, and ubiquitinated products presumed to represent nascent polypeptides (Brandman et al., 2012, Defenouillère et al., 2013). Affinity purification of either Tae2 or Rqc1 copurified the same 60S complex. Notably, 60S recruitment of Ltn1, Tae2, and Rqc1 was independent of the other two factors. By contrast, Cdc48 recruitment was dependent on Ltn1-mediated ubiquitination, Rqc1, and Tae2 (Brandman et al., 2012, Defenouillère et al., 2013). Additional functional studies of Cdc48 suggested that this AAA+ ATPase acts at a late step to extract ubiquitinated nascent chains from the ribosome for proteasomal degradation (Defenouillère et al., 2013, Verma et al., 2013).

While these studies in yeast have defined a number of components and suggested a basic framework for the RQC pathway, numerous mechanistic questions remain to be addressed. A major issue is the order of events leading to nascent chain ubiquitination: while the final RQC complex is observed on 60S subunits, their initial order of recruitment, their roles in generating 60S from 80S ribosomes, and the step at which ubiquitination is triggered are all debated. A second question is how stalled ribosomes are discriminated from translating ribosomes and what role any of these factors play in stall recognition. Third, the functions of individual components are mostly unclear. The best functional data are for Ltn1 in mediating nascent chain ubiquitination (Bengtson and Joazeiro, 2010, Brandman et al., 2012) and for Cdc48 in nascent chain extraction (Defenouillère et al., 2013, Verma et al., 2013). Finally, how protein quality control events relate to the mRNA surveillance pathways that degrade stall-inducing messages is not understood (Shoemaker and Green, 2012).

To begin addressing these and other mechanistic questions, we recently initiated efforts to develop an in vitro system that recapitulates at least some aspects of the RQC pathway (Shao et al., 2013). Using a mammalian translation extract, we demonstrated that stalled ribosome-nascent chains (RNCs) rely on Listerin for their ubiquitination. Listerin, which is normally soluble, was selectively recruited to stalled RNCs. As in yeast, both Listerin and ubiquitinated nascent chains were found predominantly on 60S ribosome subunits, which were the preferred targets of ubiquitination. This implicated a role for ribosome splitting in the RQC pathway.

Splitting of 80S ribosomes is mediated by the ATPase ABCE1 (Pisarev et al., 2010, Shoemaker and Green, 2011). Recruitment of ABCE1 to empty or stalled ribosomes requires the eRF1 and eRF3 homologs Pelota and Hbs1 (Becker et al., 2012, Pisareva et al., 2011). In the case of stalled RNCs, splitting generates a unique 60S-nascent chain-tRNA complex that was speculated to be recognized by RQC components for protein quality control (Rodrigo-Brenni and Hegde, 2012, Shao et al., 2013). Consistent with this, a GTPase-deficient Hbs1 inhibited ribosome splitting and impaired Listerin recruitment to and ubiquitination of stalled RNCs in vitro (Shao et al., 2013). In yeast, deletion of Dom34 (the Pelota homolog) stabilized the protein product of a nonstop mRNA to the same level as Ltn1 deletion or Cdc48 inactivation (Verma et al., 2013), although the basis of this effect was not analyzed further. Thus, while ribosome splitting is implicated in Listerin-mediated ubiquitination, the relationship between these two events remains mechanistically obscure.

In this study, we begin with the previously characterized RNC ubiquitination system in translation extracts and systematically dissect it down to purified factors. This reductionist strategy allowed us to define the minimal components for converting a stalled 80S-RNC into a polyubiquitinated 60S-RNC. The purified system enabled us to order the sequence of events, determine the roles played by the main factors, and determine a 60S-Listerin structure that explains the basis of 60S-selective ubiquitination. The establishment of a purified system for the central reactions in the RQC pathway should greatly facilitate detailed structural and mechanistic studies of these and downstream events.

## Results

### Purification of Stalled 80S RNCs

**Figure 1:**
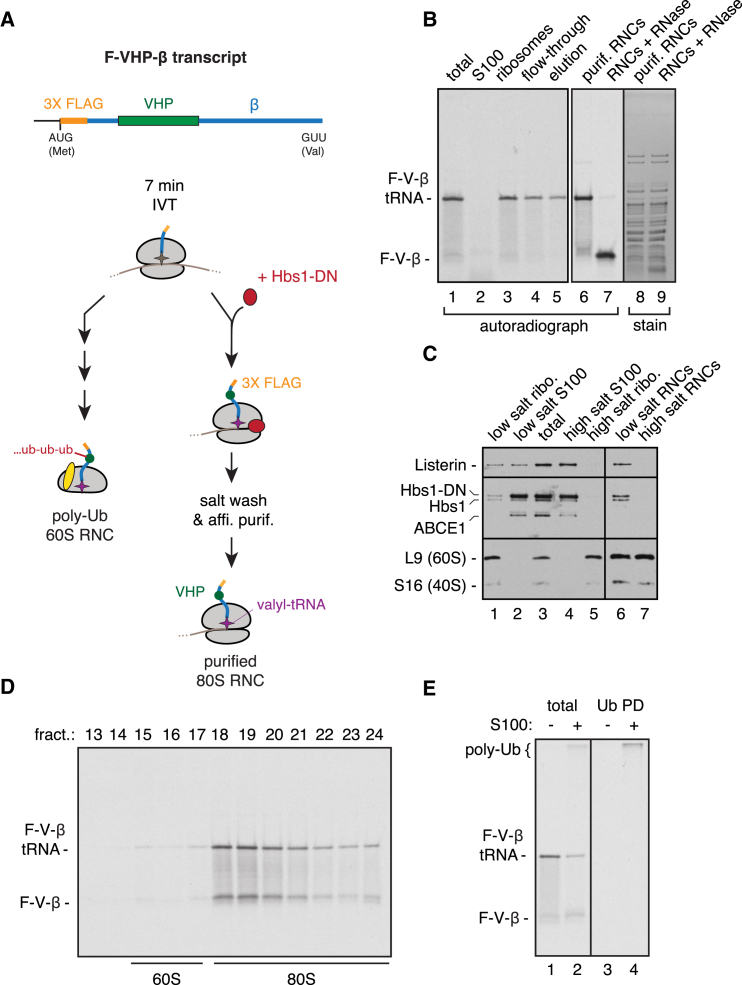
Purified Stalled 80S RNCs Are Ubiquitinated In Vitro (A) Scheme for purifying stalled 80S RNCs housing the model substrate F-VHP-β. (B) F-VHP-β was in vitro translated with ^35^S-methionine and purified by the scheme in (A). Fractions during the purification were analyzed by SDS-PAGE and autoradiography. The primary product contains an attached tRNA (F-V-β-tRNA), which is hydrolyzed by RNase to F-V-β (lane 7). Lanes 8 and 9 show Coomassie-blue-stained image of lanes 6 and 7, respectively. (C) In vitro translation reaction of F-VHP-β (lane 3) was fractionated using physiologic or high-salt conditions (lanes 1–5) and affinity purified via the FLAG tag (lanes 6 and 7), as shown in (A). All fractions were immunoblotted for Listerin, Hbs1, ABCE1, and the ribosomal proteins L9 and S16. Endogenous Hbs1 and exogenously added Hbs1-DN are labeled. (D) A 10%–30% sucrose gradient analysis of ^35^S-labeled F-VHP-β RNCs, purified as in (B), with positions of 60S and 80S indicated. (E) Purified ^35^S-labeled F-VHP-β RNCs were subjected to ubiquitination reactions with or without S-100 derived from RRL. The total reaction products and ubiquitin pull-downs (Ub PD) via the His tag were analyzed by SDS-PAGE and autoradiography. The unmodified substrate (F-V-β-tRNA) and poly-ubiquitinated (poly-Ub) products are indicated. (See also Figure S1.)

The first requirement for fully reconstituting the RQC pathway is the preparation of a well-defined starting substrate. We therefore optimized in vitro translation conditions to purify stalled 80S RNC complexes (Figure 1A). Our model nascent chain (F-VHP-β; Figure 1A) contains an N-terminal 3X-tandem FLAG tag, the autonomously folding villin head piece (VHP) domain, and the unstructured cytosolic domain of Sec61β. Radiolabeled nascent F-VHP-β was translated in vitro in a reticulocyte lysate using a stop-codon-lacking transcript whose last triplet codes for valine. A ribosome reaching the end of this transcript will stall with an intact tRNA-linked nascent chain and initiate downstream steps in the RQC pathway (Figure 1A, left option). To preclude the initiation of this pathway, we added ∼5-fold excess of a GTPase-deficient dominant negative Hbs1 (Hbs1-DN) 7 min after the start of translation, when ribosomes were still in the middle of the transcript. Upon continued elongation, Hbs1-DN should outcompete endogenous Hbs1 to prevent splitting of 80S ribosomes that reach the end of the transcript (Figure 1A, right option). Hbs1-DN inhibition was verified using a short F-β nascent chain reporter that “drops off” the ribosome upon splitting (Figures S1A and S1B available online).

F-VHP-β RNCs prepared with Hbs1-DN were then stripped of peripherally associated proteins (including Hbs1-DN) with high salt and affinity purified via the FLAG tag (Figure 1B). The high-salt wash effectively prevented various ribosome-associating factors from cosedimenting with RNCs, resulting in a purified RNC preparation that did not contain detectable amounts of Listerin, ABCE1, Hbs1, or Hbs1-DN (Figure 1C). The nascent chain remained attached to the tRNA, as evidenced by its shift with RNase treatment (Figure 1B). The small amount of tRNA loss seems to occur during gel electrophoresis as evidenced by efficient recovery of these products during purification (Figure 1B) and sucrose gradient sedimentation (Figure 1D). Coomassie staining of the purified preparation revealed the expected ribosomal protein profile (Figure 1B), and both subunits were present in their starting ratios as judged by immunoblotting (Figure 1C) and sedimentation of the radiolabeled nascent chain as an 80S particle (Figure 1D). Importantly, the nascent chain was not detectably ubiquitinated, indicating that it had not engaged the RQC pathway.

Purified 80S F-VHP-β RNCs were inert to the addition of recombinant E1, E2 (UbcH5a), ubiquitin, and energy (Figure 1E, lanes 1 and 3). This contrasts with the observed ubiquitination of a crude ribosome fraction of VHP-β RNCs isolated under physiologic conditions (Shao et al., 2013), indicating that the more rigorously purified RNCs translated in the presence of Hbs1-DN have lost key factors needed for nascent chain ubiquitination. Importantly, addition of cytosolic S-100 fraction to purified F-VHP-β RNCs generated a high-molecular weight smear that was verified to be polyubiquitination by ubiquitin pull-downs (Figure 1E, lanes 2 and 4). Like the previously characterized native RNC substrate (Shao et al., 2013), the affinity purified substrate was ubiquitinated rapidly (within 5 min) and highly processively (Figure S1C), resulting in almost all of the products migrating at the very top of the gel (Figures 1E and S1C). Sedimentation assays verified that the ubiquitinated products are ribosome associated, and ubiquitination was prevented if the F-VHP-β substrate was released from ribosomes with RNase prior to the reaction (data not shown). Thus, affinity purified 80S F-VHP-β RNC is a functional substrate of the RQC pathway poised at the step of a fully intact stalled ribosome.

### Uncoupling Ribosome Splitting and Ubiquitination of Stalled RNCs

**Figure 2:**
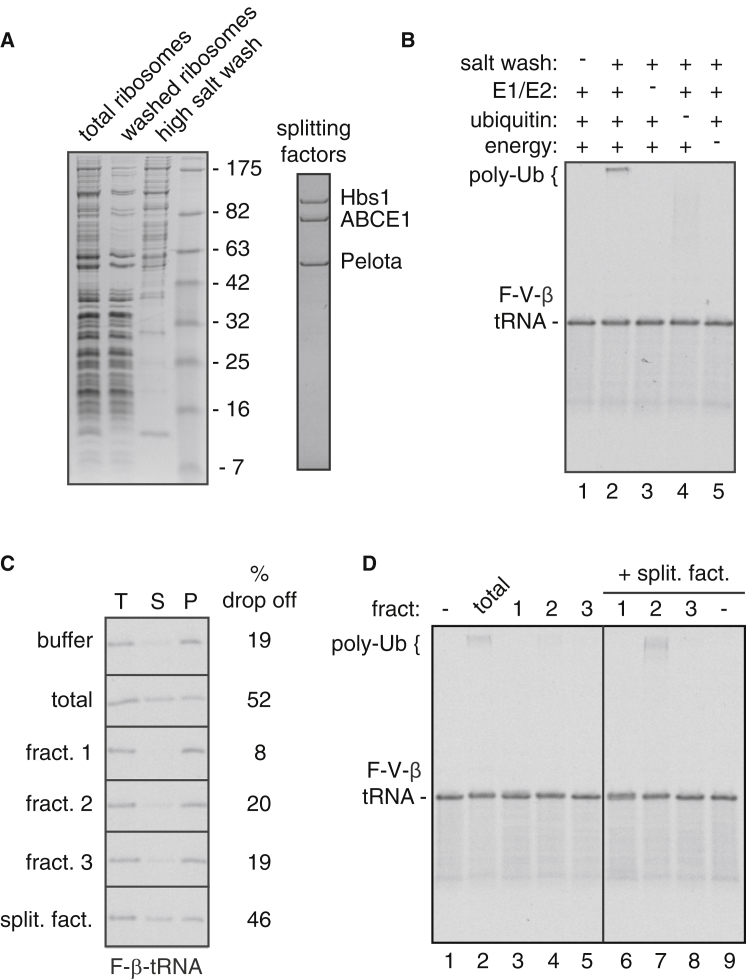
Uncoupling Ribosome Splitting and Ubiquitination Activities (A) Coomassie-stained gels of fractions from a high salt extraction of native reticulocyte ribosomes (left) and purified splitting factors (right). (B) Purified ^35^S-labeled 80S F-VHP-β RNCs were subjected to ubiquitination assays with the indicated components, and the total reaction products were analyzed by autoradiography. (C) Drop-off assay to monitor ribosome splitting using purified ^35^S-labeled F-β RNCs. The total reaction (T), supernatant (S), and pellet (P) fractions were analyzed by autoradiography, and the relative amount of F-β-tRNA in the supernatant was quantified (% drop off). (D) Purified ^35^S-labeled 80S F-VHP-β RNCs were subjected to ubiquitination assays with the indicated components, and the total reaction products analyzed by autoradiography. The unmodified substrate (F-V-β-tRNA) and poly-ubiquitinated (poly-Ub) products are indicated. (See also Figure S2.)

With a defined substrate in hand, we combined fractionation studies with functional assays to identify the steps and factors involved in the mammalian RQC pathway. Since most, if not all, reactions in this pathway occur on ribosomes, we reasoned that a ribosome salt wash may contain many of the requisite factors. Indeed, a concentrated ribosome salt wash (Figure 2A) prepared from reticulocyte lysate or HEK293T cells was sufficient to mediate ubiquitination of purified 80S F-VHP-β RNCs when supplemented with E1 and E2 enzymes, ubiquitin, and energy (Figures 2B and S2A). The salt wash also contained ribosome splitting activity, which was evident by the ∼50% nascent chain drop off using affinity purified 80S F-β RNCs as a substrate (Figure 2C).

To understand the relationship between the ubiquitination and splitting activities, we fractionated the active ribosome salt wash into three fractions via anion exchange chromatography (Figure S2B). The individual fractions had poor ribosome splitting and ubiquitination activities relative to the starting salt wash (Figures 2C and 2D), suggesting that both reactions may involve multiple components that get separated during the fractionation. Indeed, immunoblotting of these fractions revealed that the splitting factors Hbs1 and ABCE1 were efficiently separated away from each other and the E3 ligase Listerin (Figure S2B).

Since ribosome splitting is known to involve Pelota, Hbs1, and ABCE1 (Becker et al., 2011, Becker et al., 2012, Pisarev et al., 2010, Pisareva et al., 2011, Shoemaker et al., 2010), we tested these purified factors (Figure 2A) relative to the native salt wash and found them to be equally active (Figure 2C, bottom panel). Titration experiments showed that 50 nM splitting factors yielded the same degree of splitting as that seen in total translation extracts (data not shown). Using this optimized concentration of recombinant splitting factors, we retested the fractions for ubiquitination activity. Relative to the fractions in isolation, splitting activity now revealed ubiquitination activity specifically in fraction 2 at a similar level to either the starting salt wash (Figure 2D) or a mixture of fractions 1–3 (Figure S2C). These results show that ribosome splitting does not independently lead to ubiquitination, which requires additional factors. Conversely, the ubiquitination factors do not have appreciable splitting activity, but rely on splitting for ubiquitination. Importantly, all factors needed for ribosome splitting-dependent ubiquitination reside in fraction 2.

### Ribosome Splitting Precedes Listerin-Mediated Ubiquitination

**Figure 3:**
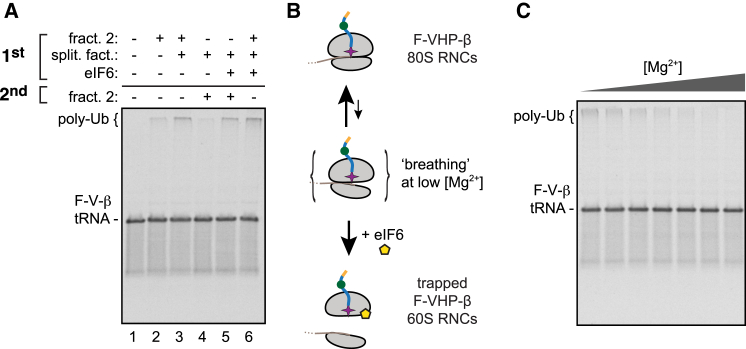
Ribosome Splitting Precedes Ubiquitination (A) Purified ^35^S-labeled 80S F-VHP-β RNCs were subjected to a two-stage ubiquitination reaction with a preincubation (1^st^) for 10 min with the indicated components, followed by another 10 min incubation (2^nd^) without or with fraction 2. Total reaction products were analyzed by autoradiography. (B) Schematic diagram illustrating the Mg^2+^-dependent dissociation of 80S RNC by eIF6. (C) Ubiquitination reactions of ^35^S-labeled 80S F-VHP-β RNCs at Mg^2+^ concentrations from 3.4 mM to 14.4 mM. All reactions contained eIF6 and fraction 2 of the ribosome salt wash.

In an attempt to understand why the splitting factors are required for RNC ubiquitination, we performed order-of-addition experiments. We found that maximal ubiquitination is only achieved when 80S RNCs are simultaneously incubated with ubiquitination factors (i.e., fraction 2) and splitting factors (Figure 3A, lane 3). In contrast, pretreating the RNCs with splitting factors before adding fraction 2 significantly diminishes ubiquitination (Figure 3A, lane 4). This suggests that ubiquitination works best when the machinery is present at the time of splitting. There are two general explanations for this result. First, it is possible that the splitting factors recruit the ubiquitination machinery to load it onto the 60S subunit at the time of splitting. Alternatively, 40S rebinding to the 60S after splitting could be a strong competing reaction to ubiquitination. In a native setting, this rebinding would be impeded because various initiation factors bind to free subunits at their interaction interface; but in our purified system, the high affinity between the subunits results in rapid reassociation.

To distinguish between these possibilities, we introduced the antiassociation factor eIF6 into our assays. This factor binds to the interface side of 60S subunits and prevents 40S association (Gartmann et al., 2010, Valenzuela et al., 1982). When eIF6 is included in the pretreatment reaction with splitting factors, fraction 2 becomes fully active for ubiquitination (Figure 3A, lane 5, compare to lane 4). Adding eIF6 at the same time as both fraction 2 and splitting factors does not further enhance ubiquitination (Figure 3A, lane 6), indicating that eIF6 does not have intrinsic stimulatory activity. These results suggest that the ubiquitination machinery recognizes 60S-RNCs but not 80S-RNCs. In this view, the splitting factors are acting primarily to reveal the substrate for ubiquitination, with 40S subunit rebinding serving as a potent (and presumably nonphysiologic) inhibitor.

To test this idea further and exclude another essential role for the splitting factors, we asked whether artificially removing 40S subunits from our 80S RNCs permits ubiquitination by fraction 2. To do this, we exploited the observation that at moderate magnesium concentrations the intersubunit interaction is sufficiently dynamic to allow 60S capture by high concentrations of eIF6 (Valenzuela et al., 1982) (see diagram, Figure 3B). We found that splitting factors could be effectively bypassed in this manner, leading to efficient ubiquitination by fraction 2 in the presence of eIF6 as magnesium concentrations were progressively lowered (Figure 3C). These observations argue that while the splitting factors may play a key role in identifying stalled ribosomes, they are not necessary for the specificity of the ubiquitination machinery for 60S over 80S RNCs; this preference is apparently intrinsic to the ubiquitination factors. Thus, ribosome splitting and ubiquitination do not need to be directly coordinated, as long as rejoining of the ribosome subunits is prevented, as would occur in vivo. We do not exclude the possibility that communication between the splitting and ubiquitination factors can improve efficiency, but our experiments have yet to provide evidence for this.

### Minimal Machinery for Stalled RNC Ubiquitination

**Figure 4:**
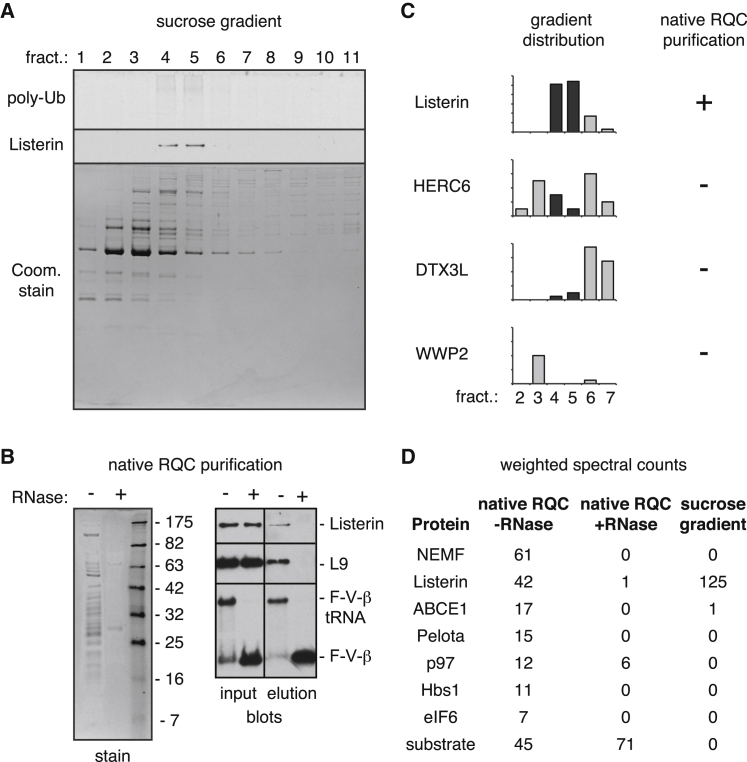
Identification of Ubiquitination Machinery (A) Fractions from the final sucrose gradient step of a purification scheme (see Figure S3A) were analyzed for RNC ubiquitination activity (top), Listerin immunoblot (middle), and Coomassie stain (bottom). (B) Affinity purification of in vitro translated F-VHP-β RNCs via the scheme in Figure S3B. The products purified in the absence or presence of RNase were analyzed by Coomassie staining (left) and immunoblotting (right). (C) Abundance profiles of E3 ubiquitin ligases across gradient fractions 2–7 from (A) identified by mass spectrometry. Fractions 4 and 5, where peak RNC ubiquitination activity was observed in (A), are highlighted in black. (D) Weighted spectral counts of select proteins identified from mass spectrometry analysis of the native RQC purification from panel B (see Table S1 for the complete list). The spectral counts for these same proteins from the sucrose gradient fractions of (A) are also shown. (See also Figure S3 and Table S1.)

To identify the factors for RNC ubiquitination via an unbiased strategy, we followed the splitting-dependent ubiquitination activity through several fractionation steps (see diagram, Figure S3A). Further separation of fraction 2 by cation exchange followed by sucrose gradient led to an enriched set of proteins with a single peak of activity (Figure 4A). No unexpected loss or heterogeneity of the activity was observed during fractionation, suggesting that the starting activity in the salt wash was fully represented in the final enriched fractions. Mass spectrometry on fractions 2–7 across the gradient provided an abundance profile for every protein, which was compared to the ubiquitination activity profile across the same fractions. This narrowed our focus to 18 cofractionating proteins (Figure S3C).

This list was compared to the set of proteins identified from a purified, native, and functional RQC complex (see Figure S3B for purification scheme). As a negative control, the native RQC sample was divided just before the affinity step, and one half was mildly digested with RNase to release the nascent chain while leaving the ribosome intact. Coomassie staining of the final native RQC preparation showed a set of proteins dependent on an intact tRNA, including the characteristic ribosomal protein profile (Figure 4B). All proteins from this experiment were identified by mass spectrometry (Table S1) and cross-checked with the candidates obtained from the functional fractionation strategy (Figure S3C).

Remarkably, the only E3 ligase common to both lists was Listerin (Figure S3C). Although four ligases were detected in the gradient fractions, only Listerin peaked in the same fractions as maximal ubiquitination activity (Figure 4C). Listerin was also the sole E3 ligase identified from the native RQC sample (Figure 4D). The only other protein we identified using this cross-validation approach was DHX29, a protein involved in 43S initiation complex formation (Pisareva et al., 2008). However, the large size of DHX29 (∼150 kD) argued against its cofractionation with Listerin via an interaction, and nothing about it pointed to a role in ubiquitination. Thus, Listerin is the ligase for RNCs in this pathway, as had been expected from earlier genetic and biochemical analyses (Bengtson and Joazeiro, 2010, Brandman et al., 2012, Shao et al., 2013).

More surprising, however, was the absence of any other obvious functional factors revealed by the unbiased activity-based purification strategy. All other proteins that cofractionated with ubiquitination activity were absent from the native sample and were too large to have copurified via Listerin. While the mammalian homologs of yeast Tae2 (NEMF), Rqc1 (TCF25), and Cdc48 (p97) could be observed in the pull-down of either native RQC complexes or native Listerin-containing complexes (Figure 4D; data not shown), they were completely absent from the gradient fractions. This indicates that, in the absence of the ribosome, these factors are not in a stable physical complex with Listerin and, more importantly, that they are not strictly required for stalled RNC ubiquitination. These observations led us to strongly suspect that Listerin plus splitting factors would be sufficient for stalled RNC ubiquitination.

**Figure 5:**
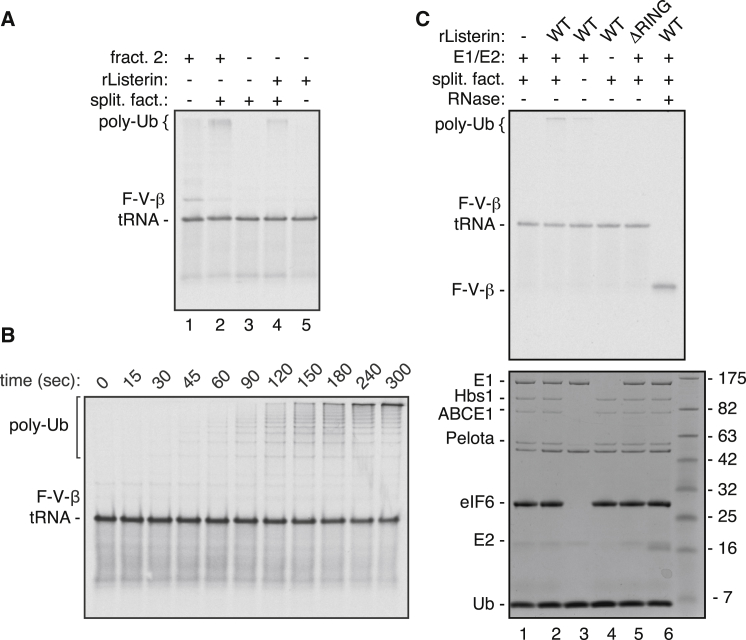
Minimal Machinery for Stalled RNC Ubiquitination (A) Ubiquitination reactions of ^35^S-labeled 80S F-VHP-β RNCs with the indicated components comparing fraction 2 of the ribosome salt wash with purified Listerin at 0.3 nM, a concentration equivalent to that found in fraction 2. (B) ^35^S-labeled 80S F-VHP-β RNCs were incubated with 75 nM E1, 250 nM UbcH5a, 10 μM ubiquitin, 50 nM splitting factors, 250 nM eIF6, 2.4 nM Listerin, and energy. Aliquots were removed at the indicated time points and analyzed by SDS-PAGE and autoradiography. (C) Ubiquitination reactions of ^35^S-labeled 80S F-VHP-β RNCs with the indicated purified factors (Listerin at 12 nM). One reaction was pretreated with RNase (lane 6) before the reaction. Shown are the autoradiograph (top) and Coomassie stain (bottom) of the same gel. (See also Figure S4)

To test this, FLAG-tagged human Listerin was purified from HEK293T cells (Figure S4A) and verified to be enzymatically active in a RING domain-dependent manner (Figure S4B). Recombinant Listerin at physiological levels was able to recapitulate the level of purified RNC ubiquitination achieved by fraction 2 in the presence of splitting factors, E1, E2, ubiquitin, and energy (Figure 5A). A time course of ubiquitination under optimized conditions of all factors showed very rapid and highly processive nascent chain ubiquitination (Figure 5B), comparable to the reaction with complete lysate (Figure S1C). Like the endogenous activity, ubiquitination by purified Listerin was dependent on ribosome splitting factors, the E1 and E2 enzymes, the RING domain, and a ribosome-bound tRNA-associated substrate (Figure 5C).

At nonphysiologically high levels, Listerin could mediate a lower degree of slower and less processive RNC ubiquitination even in the absence of splitting factors (Figure S4C). This may be due to either low affinity interactions between Listerin and 80S RNCs or some degree of spontaneous ribosome splitting to transiently expose 60S RNCs over time. To rigorously compare Listerin-mediated ubiquitination of 60S- versus 80S-RNCs, we staged the splitting and ubiquitination reactions (Figure S4D). Purified 80S RNCs were treated with splitting factors and eIF6 to generate a mixture of 60S and 80S nascent chain complexes. These complexes were separated on a sucrose gradient, and fractions containing each complex were presented to purified Listerin, E1, E2, ubiquitin, and ATP. Ubiquitination of 60S nascent chains was at least 10-fold more efficient than that of 80S nascent chains, whose ubiquitination was not convincingly detected (Figure S4E).

Additional experiments with 80S-RNCs showed that replacing Hbs1 with Hbs1-DN or adding subsets of splitting factors did not stimulate Listerin-mediated ubiquitination (data not shown). Thus, the entire set of wild-type splitting factors is needed for efficient Listerin-mediated ubiquitination. These results argue that ubiquitination is selective to 60S RNCs and that this specificity is apparently an intrinsic property of Listerin, since the splitting factor requirement can be bypassed by artificially removing the 40S subunit (Figures 3B and 3C). We conclude that the minimal machinery needed to processively ubiquitinate stalled nascent chains comprises of the E3 ligase Listerin; the splitting factors Hbs1, ABCE1, and Pelota; the E1 and E2 enzymes; ubiquitin; and energy.

### Listerin Specifically Recognizes 60S-Associated Substrates

**Figure 6:**
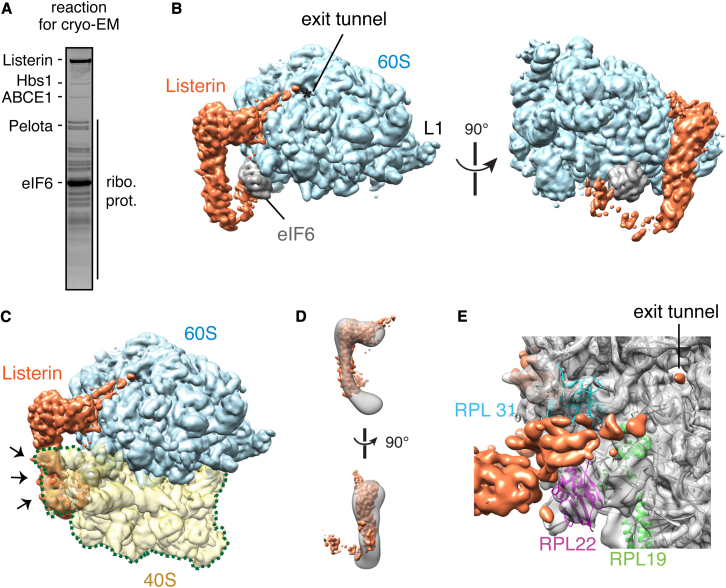
Listerin Specifically Recognizes 60S Subunits (A) Coomassie stain of reaction used for cryo-EM analysis containing ∼120 nM F-VHP-β RNCs, equimolar splitting factors (Hbs1, ABCE1, and Pelota), 1 mM ATP, 1 mM GTP, and a ∼10-fold excess of purified Listerin and eIF6. Purified proteins and ribosomal proteins (ribo. prot.) are indicated. (B) Cryo-EM reconstructions of the 60S ribosome subunit (cyan) with eIF6 (gray) and an additional density attributed to Listerin (orange). The ribosomal exit tunnel (asterisk) and L1 stalk are marked. (C) Listerin binding to 60S ribosome subunits clashes (arrows) with where a 40S subunit (yellow) would bind on an 80S ribosome. (D) Fitting of 60S-bound Listerin density into negative stain reconstructions of free Ltn1. (E) Close-up of the region of Listerin interaction with the 60S ribosomal subunit near the exit tunnel with nearby proteins (RPL22, RPL19, and RPL31) indicated. (See also Figure S5.)

A purified system for this part of the RQC pathway permitted us to examine the products of this well-defined reaction by cryo-EM. Concentrated 80S F-VHP-β RNCs were incubated with purified splitting factors, eIF6, and Listerin to assemble the complex of interest (Figure 6A). Parallel biochemical experiments verified that these complexes remained active for RNC ubiquitination (Figure S5A). The reactions were vitrified on EM grids and a data set containing 154,257 ribosome particles was collected. Initial particle classification using Relion (Scheres, 2012) identified a subset of 40,265 particles representing 60S ribosomes. Multiple rounds of subsequent classification of the 60S subset resolved two clear populations. The first, containing 11,710 particles, produced a map of the 60S subunit containing eIF6. The second, containing 9,148 particles, produced a 60S map containing eIF6 plus an additional density (Figure 6B; extra density in orange).

Because our reconstituted system contains only purified factors, and this additional density is not consistent with the known structures or positions of any of the splitting factors (Becker et al., 2011, Becker et al., 2012), we could reliably assign it to Listerin. Cryo-EM reconstructions of reactions in which Listerin was present at substantially lower concentrations failed to reveal the extra density (data not shown), further validating its assignment. The Listerin density tracks along the 60S subunit from near the 40S interface to near the exit tunnel (Figure 6B). Notably, the majority of the density does not appear to contact the ribosome, with relatively small interaction sites near either end. The clearest interaction is at the side closer to the exit tunnel, while the putative interaction near the 40S interface is poorly visible but close to eIF6.

The overall resolution of this map was determined to be 4.9 Å, while local resolution (Figure S5B) ranged from ∼4 Å for the 60S subunit and eIF6, to over 15 Å for parts of Listerin. Nevertheless, this initial view combined with biochemical and functional knowledge permits a number of provisional conclusions. The most unambiguous result is that this position of Listerin clashes extensively with the 40S subunit (Figure 6C, arrowheads). This agrees well with the requirement for subunit splitting and explains how Listerin is solely capable of discriminating 60S-substrates from 80S-nascent chains.

Definitively orienting Listerin within this density is hampered by relatively little prior structural knowledge of Listerin and the modest resolution of the current map. Nevertheless, it seems likely that the bulk of the strongly visible density corresponds to the numerous HEAT repeats predicted to form the central ∼1,000 residues of Listerin (Lyumkis et al., 2013). The overall shape and volume of this density is consistent with similar HEAT repeat domains from other proteins such as exportin and the cullins. The Listerin density in our EM map matches the general shape of isolated Ltn1 visualized by negative stain EM (Figure 6D) (Lyumkis et al., 2013). Based on the orientation assigned to the negative stain map (Lyumkis et al., 2013), the side closer to the exit tunnel in our structure would be the C terminus. Our rigorous demonstration that Listerin directly ubiquitinates the nascent chain via its C-terminal RING domain is consistent with this assignment.

Since the RING domain is not actually necessary for Ltn1 binding to the yeast ribosome (Brandman et al., 2012), the major interaction with the ribosome at the exit tunnel side most likely occurs via the poorly characterized domain between the HEAT repeats and the RING domain. While the limited resolution of Listerin precludes detailed analysis of this interaction, the proteins nearby are L22, L31, and L19 (Figure 6E). Of these, L22 would be the strongest candidate for being directly involved since it is closest to the Listerin density. Future high-resolution structural information is needed to resolve this point. The RING domain has presumably contributed to the density that trails from L22 toward the exit tunnel, some of which could conceivably be the nascent chain. This would place the RING within ∼40 Å of the nascent chain, which is well within the range of known RING-substrate distances (Deshaies and Joazeiro, 2009). Thus, Listerin binds to the 60S subunit such that 40S binding is mutually exclusive and the RING domain is near the exit tunnel.

## Discussion

The normal translation cycle can be interrupted during elongation for any number of reasons ranging from physiologic pauses to pathologic stalls. Physiologic pauses are often linked to biosynthetic processes such as polypeptide folding (Komar, 2009), mRNA splicing (Rüegsegger et al., 2001, Yanagitani et al., 2011), frameshifting (Farabaugh, 1996), and protein localization (Walter and Blobel, 1981). By contrast, pathologic stalls are typically linked to degradative fates, including mRNA decay (Doma and Parker, 2006, Kuroha et al., 2010, Shoemaker and Green, 2012), polypeptide ubiquitination (Bengtson and Joazeiro, 2010, Shao et al., 2013), and ribosomal subunit degradation (Cole et al., 2009). Correctly linking the initiating event during translation to the appropriate downstream response is critical to cellular physiology, and defects in this coupling can lead to pathologic consequences (Chu et al., 2009, Rüegsegger et al., 2001, Yanagitani et al., 2011). The mechanisms by which the translation machinery interfaces with many of these downstream pathways remain enigmatic.

We have begun to investigate this issue for pathologic stalls that trigger the RQC pathway leading to nascent polypeptide ubiquitination and degradation. A critical question is what factors participate in the decisive step of discriminating stalled ribosomes to initiate the RQC pathway. The early contender from yeast studies was Ltn1, whose recruitment to ribosomes was thought to be triggered by stalling (Bengtson and Joazeiro, 2010). Somewhat puzzlingly, most Ltn1 was primarily associated with 60S subunits, not 80S ribosomes or polysomes (Bengtson and Joazeiro, 2010, Brandman et al., 2012, Shao et al., 2013). However, undegraded nascent chains in ΔLtn1 strains accumulated on 80S ribosomes, suggesting that Ltn1 may act on this species, which eventually gets disassembled to 60S subunits (Bengtson and Joazeiro, 2010, Defenouillère et al., 2013, Verma et al., 2013).

An alternative model was proposed based on experiments in a mammalian in vitro translation extract (Shao et al., 2013). Here, production of stalled nascent chains in reactions lacking the E2 enzyme led to both 60S and 80S complexes, with only the former containing Listerin and getting ubiquitinated when E1, E2, ubiquitin, and ATP were added. Furthermore, Hbs1 depletion or mutation partially inhibited 60S formation and nascent chain ubiquitination, suggesting that ribosome splitting precedes Listerin-mediated ubiquitination. This model was consistent with the observation that Ltn1 is found on 60S together with ubiquitinated nascent chains, but distinguishing between the two views remained a challenge.

Our reconstitution and structural studies provide three lines of evidence that decisively favor ribosome splitting preceding Listerin recruitment. First, purified 80S RNCs were not effectively ubiquitinated by either native or recombinant Listerin unless splitting factors were present in the reaction. Second, artificially splitting ribosomes by 60S trapping was sufficient to stimulate nascent chain ubiquitination. Third, the Listerin-60S structure shows extensive clashes with 40S, effectively precluding interaction with 80S RNCs. We therefore conclude that ribosome splitting is upstream of Listerin recruitment and nascent chain ubiquitination. This suggests that splitting factors are a key element of stalled ribosome discrimination and initiation of the RQC pathway.

In our study, we split ribosomes using Pelota, Hbs1, and ABCE1, factors identified to recycle empty and stalled 80S ribosomes in earlier in vitro experiments (Pisareva et al., 2011). The physiologic targets of these factors are only now emerging and clearly include ribosomes at the end of truncated mRNAs (Guydosh and Green, 2014, Tsuboi et al., 2012). Because we employed a truncated mRNA in our experiments, the use of Pelota/Hbs1/ABCE1 is appropriate and physiologically relevant. Whether other types of stalls utilize the same recycling mechanism is not clear. It is possible that, depending on circumstances, factors in addition to or instead of Pelota/Hbs1/ABCE1 participate in splitting. For example, ribosome-associated factors such as Asc1/RACK1 (Brandman et al., 2012, Kuroha et al., 2010) and Hel2 (Brandman et al., 2012) seem to be important for stalling ribosomes at polybasic domains. Whether these factors are linked to the subsequent splitting reaction or involved in other types of stalls is not known. Similarly, initiation factors (Pisarev et al., 2007), release factors, or other RF3-like proteins such as Ski7 could also play a role in stall-induced splitting. The existence of multiple routes to ribosome recycling may explain why Dom34 and Hbs1 are not essential in yeast, why they seem to impact the RQC pathway in some but not other studies, and why they were not hits in genetic screens that found other RQC factors.

By contrast to this unresolved upstream step, the ligase that directly ubiquitinates the nascent chain would appear to be firmly settled. Earlier studies showed Ltn1 in yeast and Listerin in mammals as required for stalled nascent chain ubiquitination (Bengtson and Joazeiro, 2010, Brandman et al., 2012, Shao et al., 2013, Verma et al., 2013), strongly implicating this as the direct ligase. This expectation was rigorously validated in the current study, where both an unbiased activity-based purification strategy and a substrate-based coassociation strategy culminated with Listerin as the sole cofractionating E3 ligase. These results, together with our finding that recombinant Listerin can mediate ubiquitination at near-physiologic levels, establishes Listerin as the nascent chain E3 ligase for the RQC pathway.

The molecular basis of how Listerin finds its targets has been partially illuminated by our 60S-Listerin structure and addresses an otherwise puzzling issue: how can this ligase recognize its nascent chain target in only one of several potential contexts? A polypeptide in the 60S-NC complex should be efficiently targeted, while the identical polypeptide on an elongating or paused ribosome, free in solution, or as a mature protein must be scrupulously avoided. The answer lies in the fact that the ligase does not actually recognize its target, but rather the vessel housing that target. This is an emerging theme in quality control, where biosynthetic factors are increasingly found to act as adaptors between the polypeptide and a ligase (Hessa et al., 2011, Rodrigo-Brenni and Hegde, 2012).

For the RQC pathway, highly selective access is provided by removing the 40S subunit to expose regions occupied subsequently by Listerin. While this observation nicely explains Listerin specificity for 60S over 80S RNCs, it does not address why empty 60S subunits do not compete. Earlier experiments had suggested that such discrimination may involve the exposed tRNA of a 60S complex given that Listerin recruitment was more efficient when a nascent chain was present (Shao et al., 2013). Unfortunately, our current structure is poorly resolved in this region and does not address this issue.

It is possible that other RQC factors such as Tae2 or Rqc1 recognize and stabilize the tRNA. Indeed, we find that recombinant Listerin in our purified system is less stably associated with the 60S-NC complex than native Listerin in the crude salt wash (data not shown). Similarly, Ltn1 association with 60S subunits was reduced in ΔTae2 yeast strains (Defenouillère et al., 2013). It is therefore attractive to posit that, although not required for 60S-Listerin binding or nascent chain ubiquitination, Tae2 and/or Rqc1 stabilize this complex to improve efficiency or specificity of recognition. A stable complex may be important for preventing 40S rebinding and recruiting downstream factors such as Cdc48. Indeed, Cdc48 association with 60S complexes, but not ubiquitination per se, was dependent on Rqc1 (Brandman et al., 2012, Defenouillère et al., 2013). Analysis of these downstream steps remains an important goal that should be facilitated by the ability to fully reconstitute the preceding steps with purified factors.

Considering the roles provisionally assigned to the various factors from our and other studies, it would seem that the decisive and committed step of the RQC pathway is ribosome splitting. This would not only be irreversible due to loss of the 40S subunit and associated mRNA but also permits the ubiquitination machinery to efficiently access the nascent chain. This conclusion suggests that cotranslational ubiquitination (Duttler et al., 2013, Wang et al., 2013) should be mechanistically and conceptually distinguished from the RQC pathway analyzed here. While both involve ubiquitination at the ribosome, the RQC pathway is not cotranslational, as ubiquitination occurs after ribosome splitting via a ligase with 60S specificity. By contrast, cotranslational ubiquitination would necessarily involve polypeptides engaged in translation and therefore capable of being elongated (Wang et al., 2013). Since Listerin does not seem capable of binding 80S ribosomes, the mechanisms underlying cotranslational ubiquitination probably involve unidentified factors distinct from the RQC pathway. Thus, there appear to be multiple ribosome-associated pathways of quality control that warrant study in the future.

## Experimental Procedures

### Reagents and General Biochemistry

Constructs encoding VHP-β, β, Hbs1, Hbs1-DN, Pelota, and eIF6 have been described (Shao et al., 2013). Standard procedures were used to generate tagged substrate constructs and expression constructs of WT and ΔRING Listerin and ABCE1. Rabbit polyclonal antibodies against Hbs1 and ABCE1 were raised with peptide antigens (Cambridge Research Biomedicals). All other antibody reagents and in vitro translation-based methods have been described (Hessa et al., 2011, Mariappan et al., 2010, Shao and Hegde, 2011, Shao et al., 2013, Sharma et al., 2010, Stefanovic and Hegde, 2007). Details can be found in Supplemental Experimental Procedures.

### Ubiquitination and Ribosome Splitting Assays

Ubiquitination reactions of F-VHP-β and splitting reactions of F-β 80S RNCs (5 nM) were at 32°C for 5–20 min with the components indicated in individual figures. Final concentrations of purified components were as follows: 50 nM splitting factors, 250 nM eIF6, 75 nM E1, 250 nM of E2 (UbcH5a), 10 μM of His- or HA-tagged ubiquitin, and 0.3 nM to 50 nM Listerin, as indicated in the individual figure legends. Ubiquitination reactions were analyzed directly by SDS-PAGE or denatured and subjected to ubiquitin pull-downs. Splitting reactions were centrifuged in a TLA120.1 rotor at 70,000 rpm for 30 min and equal amounts of the total, supernatant, and pellet fractions analyzed by SDS-PAGE.

### Cryo-Electron Microscopy and Image Analysis

The reaction shown in Figure 6A was incubated for 15 min at 32°C and immediately vitrified on R2/2 Cu 400 mesh grids (Quantifoil) using an FEI Vitrobot. Automated data collection (EPU software, FEI) was conducted on a Titan Krios operated at 300 kV at 104,478× magnification with 1-s exposures ranging in defocus values from 2 to 3.5 μm. Particles were picked with e2boxer (Tang et al., 2007) and then classified and refined using Relion (Scheres, 2012). For comparisons in the displayed figures, all structures were low pass filtered to 8 Å. Figures were visualized and generated using Chimera (Pettersen et al., 2004).

## Accession Numbers

The EM Data Bank accession numbers for the cryo-EM density maps reported in this study are 2702, 2703, and 2704.

## Supplemental Information

Document S1. Figures S1–S5, Table S1, and Supplemental Experimental Procedures

Document S2. Article plus Supplemental Information
