# Supplementary material for: Reconstitution of a Minimal Ribosome-Associated Ubiquitination Pathway with Purified Factors
Source: Mol Cell. 2014 Sep 18;55(6):880–90. doi: 10.1016/j.molcel.2014.07.006 (PMC4175178; doi:10.1016/j.molcel.2014.07.006)
Supplement: Document S1. Figures S1–S5, Table S1, and Supplemental Experimental Procedures [file mmc1.pdf]

Molecular Cell, Volume *55*

Supplemental Information

Reconstitution of a Minimal Ribosome-Associated Ubiquitination Pathway with Purified Factors

Sichen Shao and Ramanujan S. Hegde

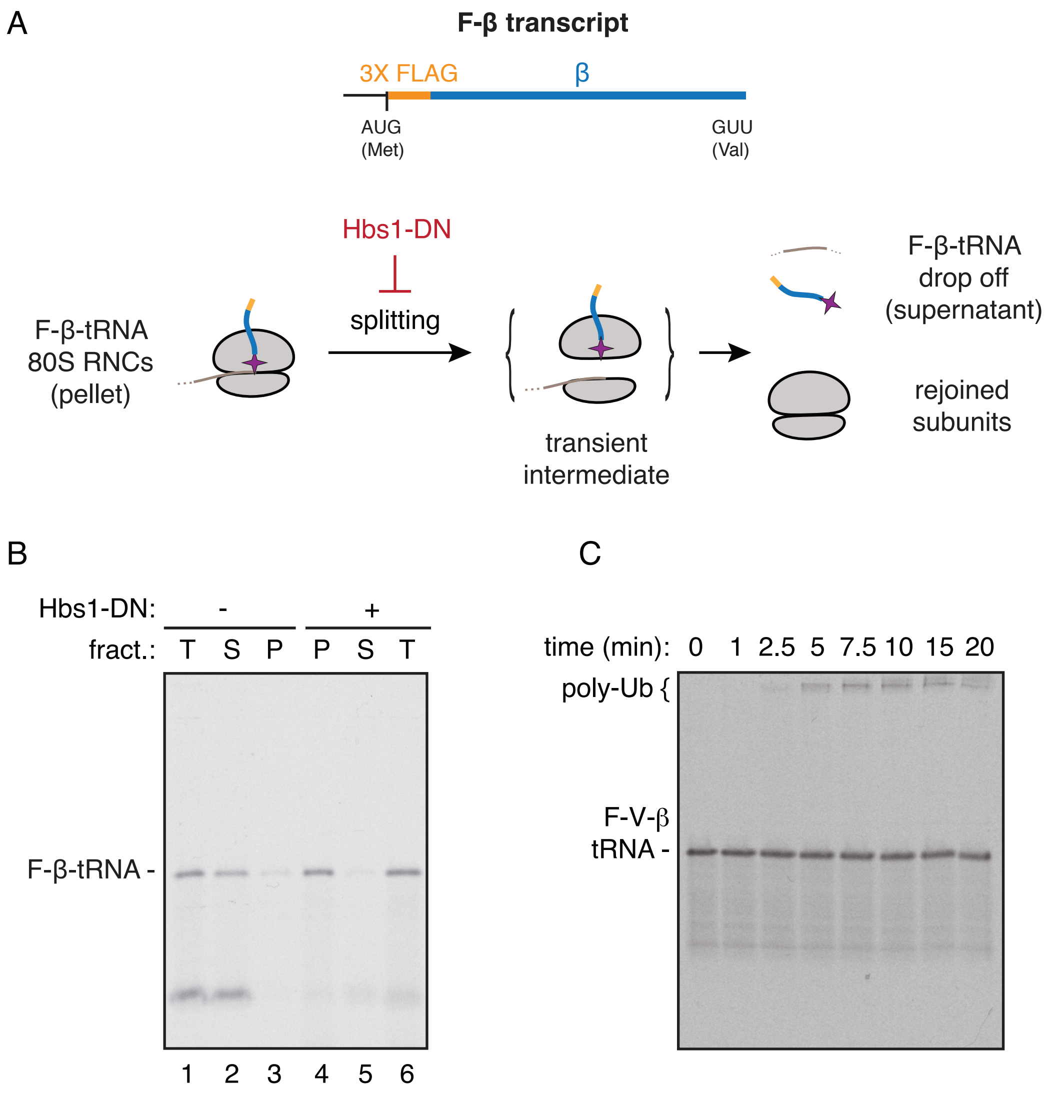

Fig. S1 (Related to Fig. 1) (A) Schematic diagram showing the principle of the drop off assay to monitor ribosome splitting. The substrate is a stop codon-less model substrate (F-β, top) containing an N-terminal 3X FLAG tag (orange) followed by the unstructured region of Sec61β (blue) and ending in a valine codon (GUU). In this assay, stalled F-β ribosome-nascent chains (RNCs) are stably ribosome-associated as long as the tRNA is intact. Ribosome splitting would transiently allow the short nascent chain-tRNA to "back-slide" out of the 60S subunit before 40S re-joining can occur. Centrifugation can distinguish drop off products (supernatant) from 80S- or 60S-associated products (pellet). Thus, an intact tRNA-attached nascent chain in the supernatant can only arise as a consequence of ribosome splitting. A dominant negative version of the splitting factor Hbs1 (Hbs1-DN) should inhibit splitting, thus preventing drop off of F-β-tRNA. (B) Drop off assay of total translation reactions (T) of F-β with or without Hbs1-DN. In the absence of Hbs1-DN, the majority of F-β-tRNA is found in the supernatant (S), indicating efficient ribosome splitting activity (lanes 1-3). Hbs1-DN effectively inhibits splitting, causing all of F-β-tRNA to remain associated with ribosomal pellets (P, lanes 4-6). (C) Autoradiograph of a timecourse of purified ^{35}S labeled 80S F-VHP-β RNC ubiquitination (see Fig. 1a) incubated with of 75 nM E1, 250 nM E2, 10 µM tagged ubiquitin, energy, and cytosolic S-100. F-V-β-tRNA denotes primary tRNA-associated nascent chain product, and poly-Ub denotes ubiquitinated substrate.

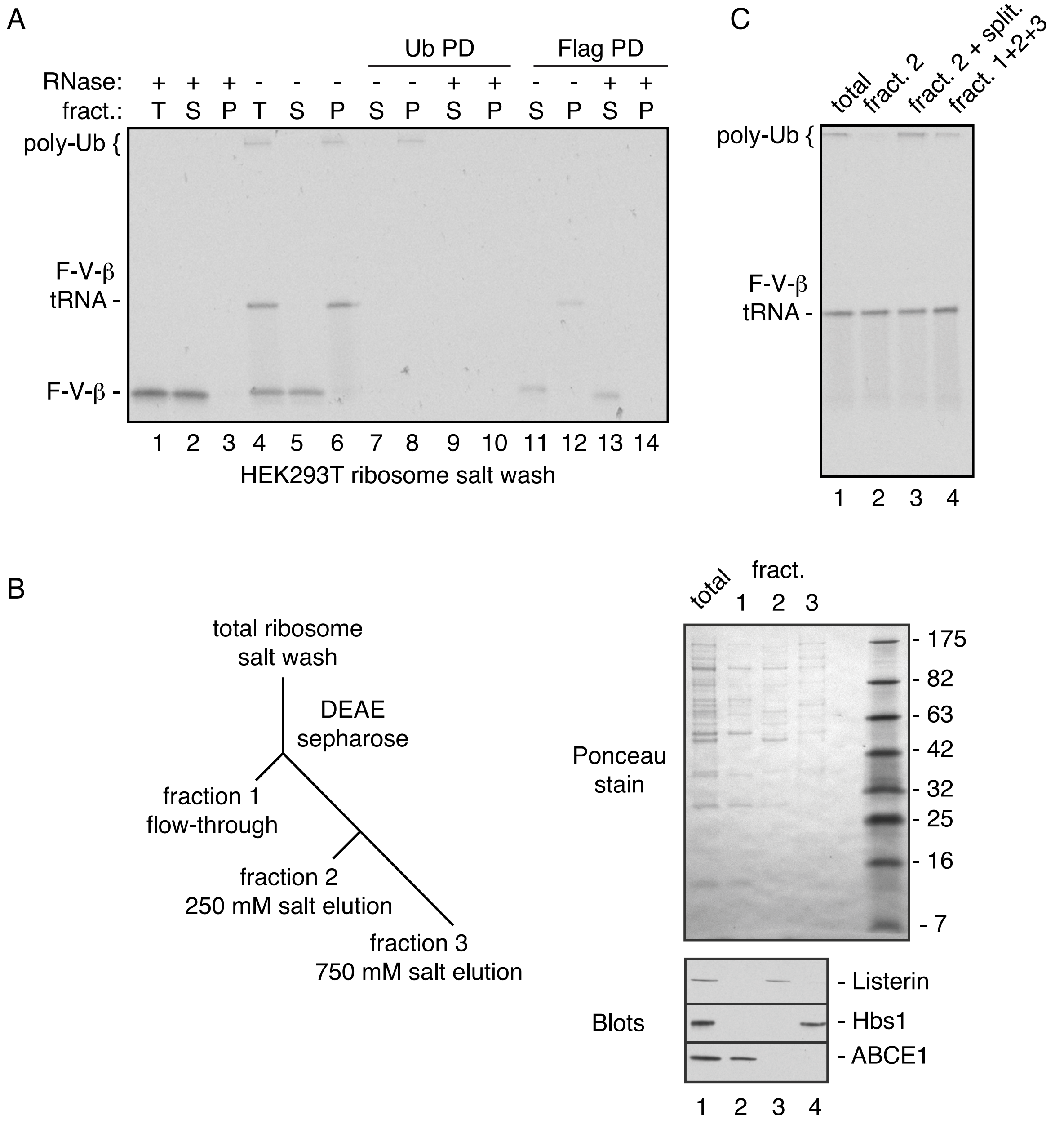

Fig. S2 (Related to Fig. 2) (A) The ribosome salt wash from HEK293T cells can mediate poly-ubiquitination (poly-Ub) of purified ^{35}S labeled F-VHP-β RNCs. Ubiquitination is dependent on an intact tRNA (compare lanes 1-3 of reactions conducted after RNase treatment to lanes 4-6). Total reactions (T) were centrifuged to isolate soluble factors (S) and ribosomal pellets (P), showing that ubiquitinated products remain ribosome-associated (lanes 6, 8, and 12). Ubiquitinated products can be distinguished by pulldowns against tagged ubiquitin (lanes 7-10). (B) Fractionation procedure to generate three fractions from total ribosome salt wash via anion exchange chromatography (left). The total ribosome salt wash (lane 1) and the individual fractions (lanes 2-4) were analyzed by SDS-PAGE and transferred to nitrocellulose membranes for immunoblotting (right). Ponceau stain of the membrane (top) demonstrates distinct protein profiles between the three fractions. Immunoblotting (bottom) reveals that the splitting factors Hbs1 and ABCE1 are fractionated away from each other and from the E3 ligase Listerin during this process. (C) Purified 80S F-VHP-β was incubated with 75 nM E1, 250 nM E2, 10 μM tagged ubiquitin, energy, and either total ribosome salt wash (lane 1), fraction 2 (lane 2), fraction 2 with recombinant splitting factors (lane 3), or a combination of fractions 1, 2, and 3 (lane 4). Reactions were directly analyzed by SDS-PAGE and autoradiography to visualize the tRNA-associated F-VHP-β nascent chains (F-V-β-tRNA) and ubiquitinated products (poly-Ub). Fraction 2 can mediate ubiquitination in the presence of recombinant splitting factors (lane 3) to the levels obtained with total ribosome salt wash (lane 1) or with the combination of all three fractions of the salt wash (lane 4).

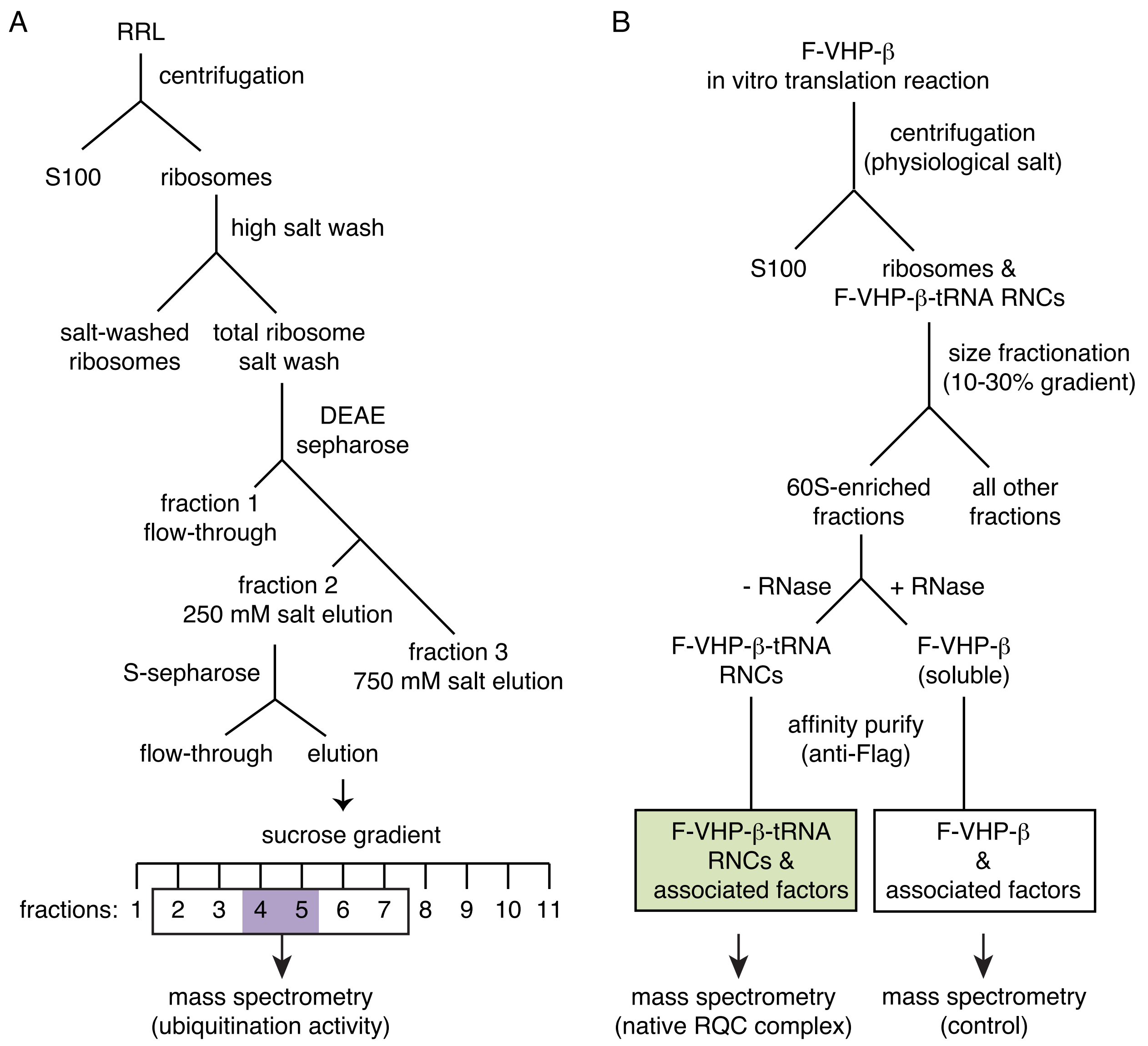

C

	2	3	4	5	6	7	Total	in Native
DHX29	5	10	77	50	27	12	181	12
Listerin	0	0	51	54	17	3	125	42
CKAP5	2	14	55	32	8	1	112	0
ARD1	3	0	31	31	3	7	75	0
SAMD9L	0	0	23	26	12	2	63	0
SMC1A	0	0	21	25	10	7	63	0
PLCB3	0	6	28	17	7	1	59	0
ECM29	0	0	24	24	6	1	55	0
SAMD9	0	0	18	23	10	2	53	0
NAA20	0	3	8	13	1	0	25	0
XRN1	0	0	6	6	0	0	12	0
SBF1	0	0	5	2	0	1	8	0
RPTOR	0	0	4	3	0	0	7	0
CBP	0	0	4	1	0	0	5	0
PARVB	0	0	2	2	0	0	4	0
CTPS2	0	0	2	3	0	0	5	0
FACI	0	0	2	1	0	0	3	0
EHD4	0	0	2	1	0	0	3	0

Fig. S3 (Related to Fig. 4) (A) Fractionation scheme for partial purification of splitting-dependent RNC ubiquitination activity. Rabbit reticulocyte lysate (RRL) was centrifuged to isolate native ribosomes and S100. A total salt wash was prepared from the ribosomes by adjusting salt levels to 750 mM KAc and centrifugation through a high salt 0.5 M sucrose cushion. The ribosome salt wash was desalted and then fractionated by anion exchange chromatography (DEAE-sepharose) into the flow-through (fraction 1) and two step elutions (fractions 2-3). Fraction 2, which contains ribosome splitting-dependent ubiquitination activity (Fig. 2b), was further fractionated by cation exchange (S-sepharose). The salt elution, which contains the ubiquitination activity, was further separated on a 5-25% sucrose gradient into 11 fractions. Of these, fractions 4 and 5 (purple) were identified to have splitting-dependent ubiquitination activity (Fig. 4a). Mass spectrometry analysis was used to identify the proteins present in gradient fractions 2-7 (see panel C). (B) Affinity purification scheme to isolate native RNC complexes containing RQC components. F-VHP-β RNCs were produced by in vitro translation (without Hbs1-DN) and pelleted via centrifugation. The ribosomal pellet was resuspended and subject to size fractionation on a 10-30% sucrose gradient. The fractions enriched for 60S ribosomal subunits were pooled and divided in two aliquots. One aliquot was treated with RNase while the other was left untreated. Both samples were then subject to affinity purification of the RNCs via the N-terminal 3X FLAG tag on the nascent chain. The elutions of the F-VHP-β RNCs and the control sample treated with RNase were analyzed by Coomassie staining (Fig. 4b) and mass spectrometry to identify associating proteins (Table S1). (C) Table of all protein identifications across the gradient fractions from panel A whose weighted spectral counts peak in the same fractions as ubiquitination activity (purple). Spectral counts for these same proteins from the native RQC sample prepared in panel B are also shown (green).

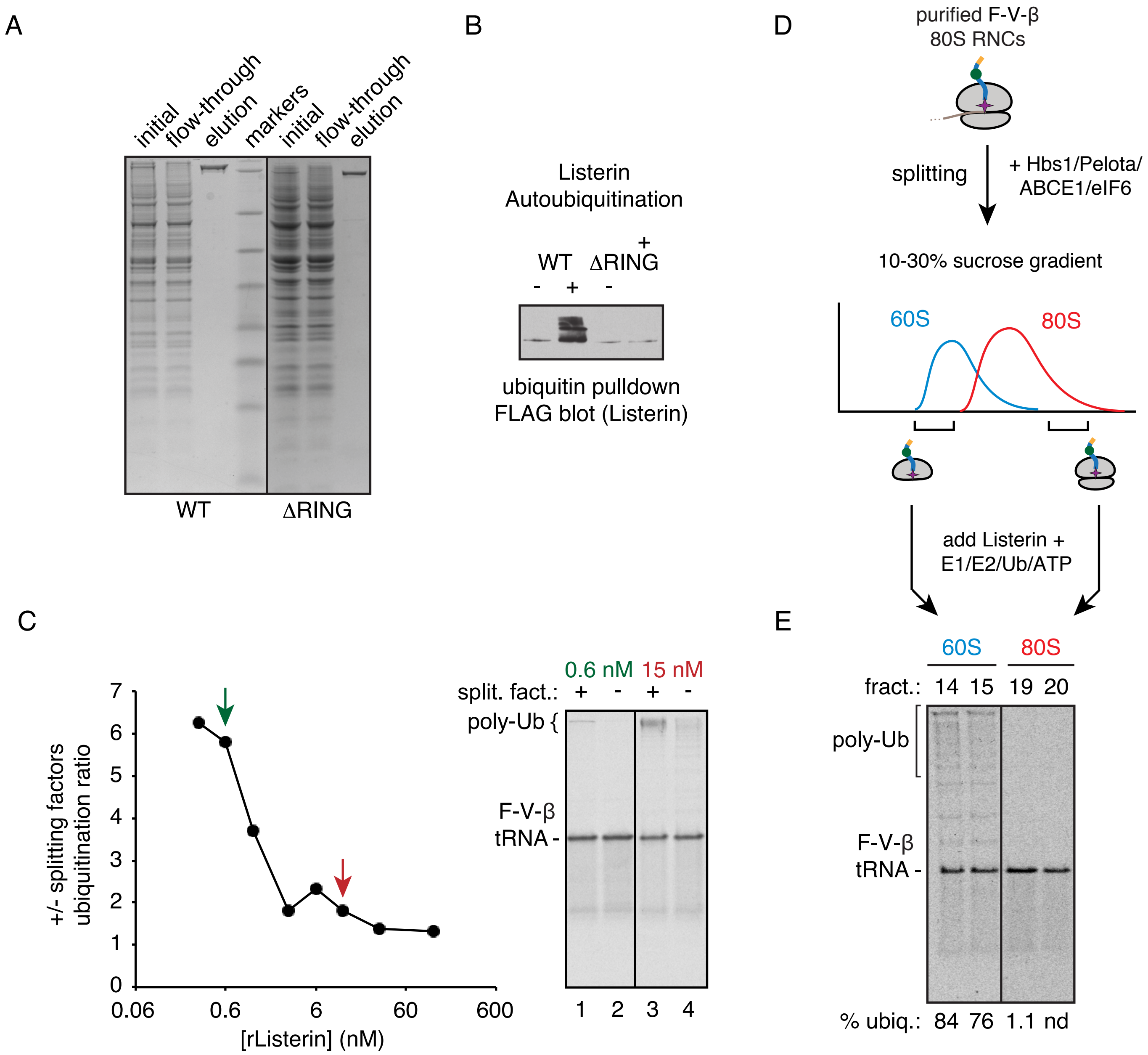

Fig. S4 (Related to Fig. 5) (A) Coomassie stained gel of Flag-tagged wildtype (WT) and ΔRING Listerin purified from transiently transfected HEK293T cells. (B) Purified WT or ΔRING Listerin was incubated with 75 nM E1, 250 nM E2 (UbcH5a), 10 µM tagged ubiquitin, and energy. Ubiquitin pulldowns from these reactions were analyzed by anti-FLAG immunoblot to detect Listerin. Note that Listerin is autoubiquitinated in a reaction that is dependent on the E1 and E2 enzymes and the RING domain. (C) Purified Listerin was titrated into reactions containing purified 80S F-VHP-β-tRNA RNCs (F-V-β-tRNA), E1 and E2 enzymes, ubiquitin, and energy with or without splitting factors (split. fact.). Reactions were analyzed by SDS-PAGE and autoradiography (representative samples shown on right). Polyubiquitination (poly-Ub) chains containing four or more ubiquitins were quantified, and the ratio of ubiquitination obtained with splitting factors to the level obtained without splitting factors was plotted against Listerin concentration (left, log scale on x-axis). Compared to physiological concentrations of Listerin (green arrow, lanes 1 and 2), which is strongly dependent on splitting factors for ubiquitination, higher amounts of Listerin (red arrow, lanes 3 and 4) ubiquitinate substrates at comparable levels with or without splitting factors. However, ubiquitination in the absence of splitting factors is not as processive (compare lanes 3 and 4). (D) Experimental scheme to isolate and compare competency of 60S- versus 80S-RNCs for Listerin-mediated ubiquitination. (E) 60S- or 80S-RNCs isolated from a splitting reaction of purified F-V-β-tRNA RNCs were incubated with 75 nM E1, 250 nM E2, 6 nM rListerin, 10 µM tagged ubiquitin, and energy. Percent ubiquitination is indicated (% of total substrate in ubiquitinated species; nd is not detected). Note that 60S RNCs are preferred substrates for Listerin-mediated ubiquitination.

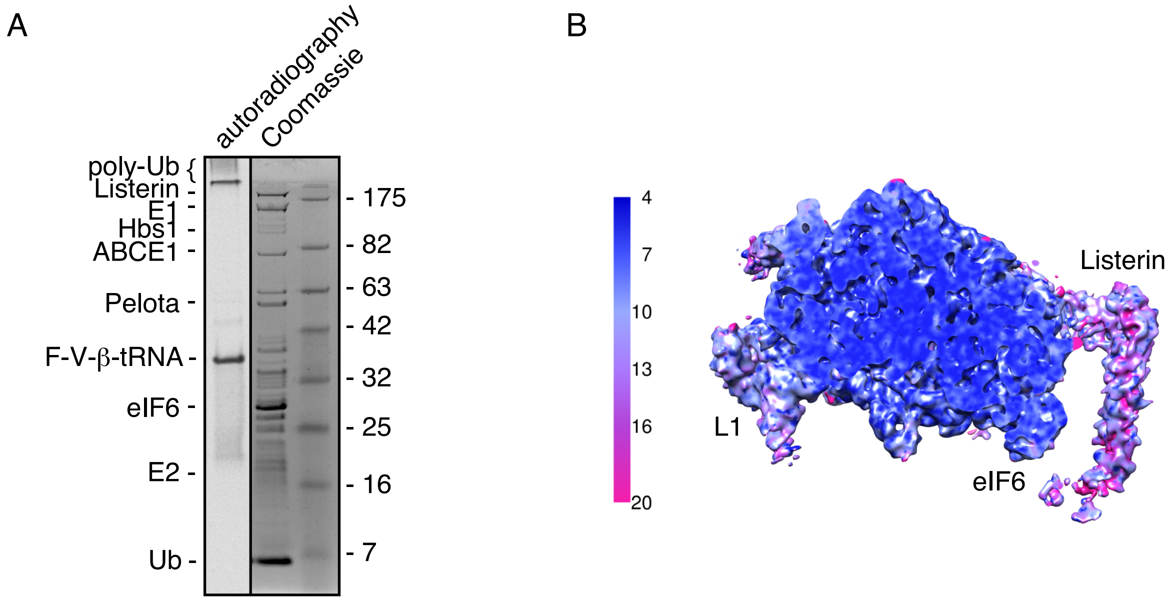

Fig. S5 (Related to Fig. 6) (A) Ubiquitination reaction of purified ^{35}S labeled 80S F-VHP-β-tRNA RNCs with all purified factors at levels comparable to those used for cryo-EM studies in Fig. 6a. Autoradiograph shows efficient nascent chain ubiquitination; Coomassie stained image of the same gel is also shown. (B) Local resolution map of 60S-Listerin reconstruction showing that the ribosome and eIF6 are much better resolved than the Listerin density.

Specific for tRNA

ID	+ RNase	- RNase
PAIRB	3	75
NEMF	0	61
LTN1	1	42
TPP2	1	37
SLFN14	1	24
TTC37	0	31
LARP1	0	23
SKIV2	0	21
ABCE1	0	17
PABP	0	17
PFKL	2	13
PELO	0	15
DJC21	0	16
NACA	0	13
YBOX1	0	13
TCPG	0	13
FOCAD	0	13
DHX29	0	12
HBS1L	0	11
DESP	0	9
CCT8	0	9
SMBP2	0	10
DNAJC13	0	9
GROL	0	8
APOD	0	8
PSMD3	1	7
PA2G4	1	7
DHX9	0	8
MARR	0	7
ZNF622	0	7
SND1	0	7
HS90B	0	7
CASP14	0	6
CCT2	0	7
C6orf86	0	5
PSB5	1	4
C1TC	0	6
ABCF1	0	5
IFRD1	0	5
CASB	1	3
TGM3	1	3
LOX12	1	3
TCPD	0	4
AGO2	0	4
CLH1	0	3
AP2A2	0	3
G3P	0	3
ZC3HF	0	4
SMC2	0	3
GEMIN5	0	3
DSC1	0	3
TCPA	0	3

Not specific for tRNA

ID	+ RNase	- RNase
IGKC	98	63
IGH1M	75	57
SC61B	47	31
VIL1	24	14
HBB	12	22
H13	32	0
KRT6C	14	17
HSP7C	13	18
ERCC4	12	14
TRY10	17	9
PRSS1	12	6
VCP	6	12
PYR1	8	8
HBA	5	6
PSMD2	8	3
RFET7	4	6
DCD	3	6
BCEN2424	6	3
PSMD1	4	5
CASA2	7	1
PRS4	5	3
PRS7	3	5
MALE	5	2
CASA1	4	2
PSMD4	3	3
PRS8	2	4
PSD11	2	3
LGB2	5	0
PSB6	2	2
THIO	1	2
RANBP10	2	1
C27C1	1	2
PRS10	3	0

Translation-related factors

ID	+ RNase	- RNase
EF2	7	146
REF	50	29
EIF3A	0	44
EIF3C	1	24
EIF3B	5	6
IF4GI	0	12
EIF3E	2	18
IF2G	4	5
EIF3D	1	7
EIF6	0	7
IF2B	1	5
EI2BD	0	6
EI2BE	2	5
IF2A	0	4
IF2P	0	4
IF4A1	0	3
EIF3I	0	3
EIF3G	1	0
FARSB	0	10
LARS	0	8
DARS	0	7
IARS	0	6
EPRS	0	7
FARSA	0	3
QARS	1	1

60S proteins

ID	+ RNase	- RNase
RPL3	0	166
RPL5	0	107
RPL7A	0	89
RPLP0	1	81
RPL18	12	71
RPL4	0	70
RPL7	3	87
RPL13	3	61
RPL24	3	56
RPL6	0	58
RPL11	6	39
RPL13A	4	39
RPL21	0	41
RPL8	1	37
RPL23A	4	33
RPL10	0	36
RPL30P	2	33
RPL12	3	32
RPL19	0	34
RPL30	2	38
RPL17	2	30
RPL23	2	30
RPL27A	2	28
RPL26	1	30
RPL14	0	31
RPL27	1	28
RPL35A	1	28
RPL18A	0	28
RPL31	2	26
RPL15	1	26
RPL28	2	26
RPLP2	0	26
RPL38	1	25
PL34	1	22
RPS7	2	20
RPL36A	1	21
RPL36	2	18
RPL22	1	17
RPL9	0	16
RPL35	1	15
RPL37A	0	17
RPL10A	0	13
RPL30.1	0	10
RPL32	0	11
RPL22L	0	9
RPLS2	0	8
RPLA1	0	4
RPL37	0	3

40S proteins

ID	+ RNase	- RNase
RPS2	3	125
RPS3	6	124
GBLP	2	93
RPS3A	7	71
RPS4X	2	69
RPS27A	23	40
RPS6	0	60
RPS18	8	51
RPS8	0	55
RPS9	5	43
RPSA	3	42
RPS16	1	44
RPS19	5	37
RPS27L	3	38
RPS15A	3	35
RPS21	2	53
RPS23	0	29
RPS14	2	26
RPS5	2	29
RPS12	2	29
RPS25	1	23
RPS24	0	32
RPS20	0	20
RPS11	0	18
RPS17	0	19
RPS29	2	14
RPS13	3	16
RPS28	1	13
RPS30	0	14
RPS26.2	0	13
RPS27	0	8
RPS10	0	7
RSP17	0	6

Table S1 (Related to Fig. 4) Mass spectrometry of the native mammalian RQC complex.
F-VHP-β RNCs produced by in vitro translation were purified to isolate native RQC complexes with and without RNase A treatment as in Fig. S3b. Gel slices from the purified samples (Fig. 4b) were analyzed by mass spectrometry. All identified proteins with more than 3 spectral counts are listed. The F-VHP-β substrate and components thought to be involved in the RQC pathway from earlier work are highlighted in yellow and green, respectively.

Supplemental Experimental Procedures

Plasmids and antibodies

The constructs encoding untagged VHP-β and β, WT and dominant negative Hbs1, Pelota, and eIF6 have been described (Shao et al., 2013). For isolation of 80S RNCs, the open reading frames of VHP-β and β were cloned into an SP64 vector containing an N-terminal 3X tandem Flag tag using standard protocols. The human open reading frames of Listerin and ABCE1 were cloned into a pcDNA-based vector encoding an N-terminal 3X tandem Flag tag using standard procedures. ΔRING Listerin was generated using Phusion mutagenesis. Anti-Listerin (Abcam), anti-Flag (Sigma), anti-RPL9 (Santa Cruz), and anti-RPS16 (Santa Cruz) antibodies have been described (Shao et al., 2013). Rabbit polyclonal antibodies against Hbs1 and ABCE1 were generated with KLH-conjugated peptide antigens [Hbs1-ARHRNVRGYNYDEDFE, ABCE1-IKDVEQKKSGNYFFLDD] (Cambridge Research Biomedicals). Anti-Flag resin and 3X Flag peptide used for affinity purification were obtained from Sigma.

In vitro transcription and translation

All transcripts were generated using PCR products. The 5' primer anneals just before the SP6 promoter in the SP64 vector. The 3' primer to generate F-VHP-β anneals to residues 63-68 of the Sec61β sequence and appends an additional valine residue to generate a stable valyl-tRNA (see Fig. 1a). The 3' primer to generate F-β truncates after residue 40 of the Sec61β open reading frame and appends the residues "MLKV" to generate stable RNCs (Fig. S1a). Transcription reaction were carried out with PCR templates and SP6 polymerase for 1 hour at 37°C. The transcripts were used directly for translation in an in vitro rabbit reticulocyte (RRL) system as previously described (Shao et al., 2013; Sharma et al., 2010).

Purification of salt-washed 80S RNCs

A translation reaction of F-VHP-β or F-β was assembled and incubated at 32°C. After 7 minutes, an excess (~50 nM) of dominant negative Hbs1 (Hbs1-DN) was added and the reaction was allowed to proceed for an additional 18 minutes (see diagram, Fig. 1a). Translation reactions were placed on ice and adjusted to a final concentration of 750 mM KAc and 15 mM $MgAc_2$. 1 mL of salt-adjusted translation reaction was layered on a 1.6 mL high salt sucrose cushion (50 mM Hepes, pH 7.4, 750 mM KAc, 15 mM $MgAc_2$, 0.5 M sucrose) and spun for 1 hour at 100,000 rpm in a TLA100.3 rotor in a Beckman Optima-Max ultracentrifuge. For isolation of RNCs under physiological conditions (Fig. 1c), no adjustments were made to the salt concentrations and ribosomes were isolated from translation reactions through a 0.5 M sucrose cushion in physiological salt (50 mM Hepes, pH 7.4, 100 mM KAc, 2.5 mM $MgAc_2$). The ribosome pellet was resuspended in RNC buffer (50 mM Hepes, 100 mM KAc, 5 mM $MgAc_2$, 1 mM DTT) and incubated with anti-Flag resin for 1-1.5 hour at 4°C. The resin was washed three times with RNC buffer with 0.1% Triton X-100, three times with 50 mM Hepes, 250 mM KAc, 5 mM $MgAc_2$, 0.5% Triton X-100, 1 mM DTT, and three times with RNC buffer. Bound RNCs were eluted with 0.1 mg/ml 3X Flag peptide in RNC buffer. Two elutions of one column volume each were conducted for 30 minutes at room temperature and combined.

Preparation of S-100 and ribosome salt wash

Rabbit reticulocyte lysate (RRL) S-100 was prepared by spinning crude RRL in a TLA100.3 rotor at 100,000 rpm for 40 minutes. The supernatant was used directly for downstream

assays (Fig. 1e). The pellet containing crude ribosomes was resuspended into ribosome wash buffer (20 mM Hepes, pH 7.4, 100 mM KAc, 1.5 mM $MgAc_2$, 0.1 mM EDTA, 1 mM DTT) and re-pelleted over a 0.5 M sucrose cushion in ribosome wash buffer. The ribosome pellet ("native ribosomes") was resuspended in 20 mM Hepes, pH 7.4, 200 mM KAc, 1.6 mM $MgAc_2$, 10% glycerol, 1 mM DTT and stored at -80°C. To generate a ribosome salt wash (Fig. 2a), native ribosomes were adjusted to a final concentration of 750 mM KAc, 15 mM $MgAc_2$. 1 mL of the ribosomes was layered over a 1.6 mL high salt 0.5 M sucrose cushion and centrifuged in a TLA100.3 rotor at 100,000 rpm for 1 hour. The supernatant was saved and either dialyzed against RNC buffer containing 10% glycerol for functional assays or desalted over a PD-10 column (GE Healthcare) equilibrated in RNC buffer for further fractionation steps (Fig. S2b). To generate a ribosome salt wash from tissue culture cells (Fig. S2a), HEK293T cells were lysed in 50 mM Hepes, pH 7.4, 100 mM KAc, 2.5 mM $MgAc_2$, 0.5% Triton X-100, 1 mM DTT, 1X protease inhibitor cocktail (Roche) and spun in a tabletop centrifuge for 10 minutes at 4°C. Ribosomes and the accompanying ribosome salt wash from the post-nuclear supernatant were then isolated exactly according to the procedure for reticulocyte lysate above.

Fractionation of ribosome salt wash

Desalted total ribosome salt wash was passed over a DEAE-sepharose column equilibrated in RNC buffer (Fig. S2b). The flow-through (fraction 1) was collected, and the column was washed extensively with RNC buffer. The first step elution (fraction 2) was with 50 mM Hepes, 250 mM KAc, 5 mM $MgAc_2$, 1 mM DTT. The column was washed three times with the first elution buffer before the second step elution (fraction 3) with 50 mM Hepes, pH 7.4, 750 mM KAc, 5 mM $MgAc_2$, 1 mM DTT. For functional assays, all elutions were dialyzed against RNC buffer containing 10% glycerol. Fraction 2 after the DEAE step purification was further fractionated over an S-sepharose column equilibrated in RNC buffer (Fig. S3a). The flow-through and elution with 750 mM KAc were collected and analyzed for splitting-dependent ubiquitination activity. The elution was then fractionated over a 5-25% sucrose gradient in RNC buffer in a TLS-55 rotor at 55,000 rpm for 5 hours 4°C with the slowest acceleration and deceleration settings. Eleven 200 μL fractions were collected from the top and analyzed for activity (Fig. 4a). Fractions 2-7 were then processed for mass spectrometry analysis (Fig. 4d).

Affinity purification of native nascent chain-tRNA RQC complexes

An 8 ml translation reaction of F-VHP-β (without Hbs1-DN) was incubated for 30 minutes at 32°C to permit generation of native RQC complexes (see Fig. 1a). 1 ml aliquots were layered over 1.6 mL sucrose cushions containing 0.5 M sucrose in physiological salt (50 mM Hepes, pH 7.4, 100 mM KAc, 2.5 mM $MgAc_2$, 1 mM DTT) and spun in a TLA110 rotor at 100,000 rpm for 1 hour at 4°C. The ribosomal pellets were resuspended in a total of 800 μL of RNC buffer. 200 μL was loaded onto each of four 4.8 mL 10-30% sucrose gradients and spun in a MLS-50 rotor at 50,000 rpm for 2 hours at 4°C with the slowest acceleration and deceleration settings. Twenty-five 200 μL fractions were collected from the top. Based on previous characterizations of this gradient, fractions 14-17, which contain the majority of 60S ribosomal subunits (Shao et al., 2013), were pooled for downstream purification. The pooled fractions were divided into two halves. One half was treated with 50 μg/mL RNase A at room temperature for 15 min to generate a negative control of non-tRNA specific interacting proteins. Both samples (with and without RNase treatment) were incubated with 50 μL of packed anti-Flag resin at 4°C for 1 hour. The resin was washed three times in RNC buffer with 0.1% Triton X-100, three times with 50 mM Hepes, pH 7.4, 250 mM KAc, 5 mM

$MgAc_2$, 0.5% Triton X-100, 1 mM DTT, and then another three times with RNC buffer with 0.1% Triton X-100. Elutions were carried out with 0.15 mg/mL 3X Flag peptide in RNC buffer with 0.1% Triton X-100 for 30 minutes at room temperature. Two sequential elutions were combined and subject to TCA precipitation and subsequent analysis by SDS-PAGE (Fig. 4b) and mass spectrometry (Table S1).

Ubiquitination and ribosome subunit splitting assays

For most functional assays, F-VHP-β RNCs at 5 nM was incubated with S-100 (Fig. 1e), ribosome salt wash, or ribosome salt wash fractions (Fig. 2b-2d, S2a, and S2c) at ratios normalized to their levels in total lysate based on the fractionation procedure. With purified components, increasing the concentration of RNCs to ~60-100 nM had little qualitative effect on the results (Fig. S5a). Recombinant splitting factors (Fig. 2a) were added to a final concentration of either 50 nM or to equimolar levels as the RNCs in the reaction (Fig. 2c, 2d, 5b, 5c, and S5a). Recombinant Listerin was added at a range of 0.3 nM ("physiological" concentration by comparison to fraction 2) and titrated up to 120 nM (Fig. S4c). Unless otherwise indicated, all reactions contained 75 nM E1, 250 nM of E2 (UbcH5a), and 10 μM of His- or HA-tagged ubiquitin (all from Boston Biochem). Where applicable (Fig. 1b, 4c, and 5c), reactions were treated with 50 μg/mL of RNase A for 10 minutes at room temperature before the addition of ubiquitination reagents. Reactions were conducted at 32°C for 5-20 minutes, and either analyzed directly by SDS-PAGE and autoradiography, subject to additional centrifugation steps, or rapidly denatured for ubiquitin pulldowns as indicated in individual figure legends. To monitor ribosome subunit splitting (Fig. 2c), isolated 80S F-β RNCs were incubated with the desired components at the same concentrations and conditions as for ubiquitination reactions. The reactions were then centrifuged in a TLA120.1 rotor at 70,000 rpm for 30 minutes at 4°C. The supernatant was saved and the pellet was resuspended in the original reaction volume's worth of 1% SDS. Equal amounts of the total, supernatant, and pellet fractions were loaded for SDS-PAGE analysis and autoradiography.

Purification of recombinant proteins

His-tagged eIF6 and Pelota were purified via Ni-NTA using standard procedures. Briefly, the constructs were transformed into BL21(DE3) cells. Cells were grown under antibiotic selection, induced overnight at 16°C with 0.2 mM IPTG, and harvested and lysed with a microfluidizer in lysis buffer (1X PBS, 250 mM NaCl, 10 mM imidazole, 1X protease inhibitor cocktail, 1 mM DTT). Lysates were clarified by centrifugation, bound to a Ni-NTA column by gravity flow, and washed extensively with lysis buffer. Elution was carried out in lysis buffer containing 250 mM imidazole. Peak fractions were pooled and dialyzed into RNC buffer with 10% glycerol. Flag-tagged Hbs1, Hbs1-DN, and ABCE1 were transfected into HEK293T cells using TransIT 293 (Mirus). Cells were split 1:4 the day after transfection and purified two days later. Generally, eight 10 cm dishes of confluent cells were harvested in ice cold PBS and lysed in a total volume of 1.5 mL of lysis buffer (50 mM Hepes, pH 7.4, 100 mM KAc, 5 mM $MgAc_2$, 0.5% Triton X-100, 1 mM DTT, 1X protease inhibitor cocktail). Lysates were spun for 10 minutes in a tabletop centrifuge at 4°C and the post-nuclear supernatant was incubated with 100 μL of packed anti-Flag resin for 1-1.5 hour at 4°C. The resin was washed three times with lysis buffer, three times with 50 mM Hepes, pH 7.4, 400 mM KAc, 5 mM $MgAc_2$, 0.5% Triton X-100, 1 mM DTT, and three times with RNC buffer. Elutions were carried out with one column volume of 0.1 mg/mL 3X Flag peptide in RNC buffer at room temperature for 30 minutes. Two sequential elutions were combined for downstream assays. Flag-tagged WT and ΔRING Listerin (Fig. 5) were transfected into HEK293T cells using TransIT 293 (Mirus), split 1:4 the day after transfection, and purified

two days after. Sixteen 10 cm dishes of confluent Listerin-expressing cells were harvested in ice cold PBS and lysed in ~1 mL 25 mM Hepes, pH 7.4, 125 mM KAc, 15 mM $MgAc_2$, 1:20,000 dilution of RNasin (Promega), 50 μg/mL cycloheximide, 100 μg/mL digitonin, 1 mM DTT, 1X protease inhibitor cocktail (Roche), rotating at 4°C for 10 minutes. The lysates were then spun in a tabletop centrifuge at 4°C for 10 minutes. The post-nuclear supernatant was centrifuged in a TLA100.3 rotor at 100,000 rpm for 40 minutes at 4°C. The S-100 was incubated with 100 μL of packed anti-Flag resin for 1-1.5 hour at 4°C. The resin was washed three times in the buffer used to lyse the cells, three times in 25 mM Hepes, pH 7.4, 400 mM KAc, 5 mM $MgAc_2$, 100 μg/mL digitonin, 1 mM DTT, and three times in 25 mM Hepes, pH 7.4, 250 mM KAc, 5 mM $MgAc_2$, 10% glycerol, 50 μg/mL digitonin, 1 mM DTT. Elutions were carried out with one column volume of 0.1 mg/mL 3X Flag peptide in the final wash buffer at room temperature for 30 minutes. Two sequential elutions were combined for downstream assays.

Miscellaneous biochemistry

Samples for ubiquitin pulldowns were denatured by boiling in 1% SDS, 0.1 M Tris, pH 8. His-ubiquitin pulldowns were performed with Ni-NTA in 1X PBS, 250 mM NaCl, 0.5% Triton X-100, 10 mM imidazole. HA-ubiquitin and Flag-ubiquitin pulldowns were performed in 1X PBS, 250 mM NaCl, 0.5% Triton X-100 with either anti-Flag resin or polyclonal anti-HA antibody with Protein A agarose beads. All samples were incubated with resin for 1-2 hours rotating at 4°C, washed twice in the pulldown buffer and eluted with protein sample buffer for SDS-PAGE analysis. 10 mM EDTA was included in the elution of the pulldowns with Ni-NTA to chelate the nickel. TCA precipitations were carried out at 4°C for 10 minutes with 15% TCA and 0.5% Triton X-100. Proteins were washed once with cold acetone, air dried, and solubilized directly protein sample buffer for SDS-PAGE analysis. SDS-PAGE was performed with 7.5% or 10% Tris-tricine gels. For immunoblots, gels were transferred to 0.2 μm nitrocellulose membrane (Bio-Rad) and blocked in 5% milk in PBS with 0.1% Tween-20. Primary antibodies were incubated at the following concentrations: 1:500 anti-Listerin, 1:4000 anti-Hbs1, 1:4000 anti-ABCE1, 1:100 anti-RPL9, 1:100 anti-RPS16, 1:500 anti-TCF25, 1:5000 anti-Flag. For autoradiography, gels were fixed and stained in 0.1% Coomassie R250 in 40% ethanol, 10% acetic acid for at least 20 minutes. After destaining in 10% ethanol, 7.5% acetic acid, gels were dried and exposed to MR film (Kodak). For mass spectrometry, SDS-PAGE gels were stained with colloidal Coomassie stain (Pierce) and destained in water. For gradient fractions, gels were run approximately only 1 inch into the separating gel. ~1 mm gel slices were excised from each lane and analyzed for protein identifications by in-house mass spectrometry facilities.

Cryo-electron microscopy and image analysis

Reactions containing approximately 120 nM F-VHP-β RNCs, equimolar amounts of splitting factors, and approximately 10-fold excess of Listerin and eIF6 (Fig. 6a) were incubated for 15 minutes at 32°C and immediately frozen on grids. Samples were applied to glow-discharged R2/2 Cu 400 mesh grids (Quantifoil) coated with a thin (~50 Å) layer of continuous carbon and vitrified with an FEI Vitrobot. Grids were screened on a Tecnai T12. Several datasets collected on a Polara allowed for sample optimization, mainly to increase the amount of eIF6 and Listerin in the reaction to favor stable 60S subunits and complex formation. Overnight automated data collection (EPU software, FEI) was conducted on a Titan Krios operated at 300 kV, at 104,478X magnification. One second exposures yielding a total dose of 35 electrons/$Å^2$ were collected with defocus values ranging from 2 to 3.5 μm. Particles were picked with e2boxer (EMAN2) (Tang et al., 2007), yielding an initial dataset

of 172,267 particles, which was processed through Relion (Scheres, 2012). After 2D classification, 154,257 particles underwent 3D classification. 80S and 60S ribosome classes, consisting of 70,883 and 40,265 particles, respectively, were combined and refined separately. The 3D refinements were then used to reclassify the corresponding datasets. For the 80S ribosomes, this yielded classes of 80S ribosomes with P and E-site tRNA density (44,255 particles) and of empty ribosomes with and without eEF2 bound. The 80S ribosome with tRNA density was then individually refined, corrected for beam-induced movement and radiation damage by movie frame processing (Bai et al., 2013), and further refined. This resulted in a final resolution, according to gold-standard FSC criteria, of 3.8 Å for the 80S with P and E-site tRNA density. For the 60S ribosomes, the first 3D classification yielded a class of 26,925 particles with a weak additional density that corresponded to Listerin. This class was further refined and used for another round of 3D classification. Listerin-containing particles from this classification step (15,188) were refined and corrected for movement and radiation damage. This refined model was used for another round of classification with a mask for the Listerin density, yielding 9,148 particles with clear Listerin density (Fig. 6b and 6c). Refinement of this class yielded a final structure with a resolution of 4.9 Å, although the local resolution for Listerin itself ranges from 5 to more than 15 Å (Fig. S5b). After the first round of 3D classification, classes of empty 60S subunits (11,710 particles) were also combined and refined to a final resolution of 4.4 Å. For comparisons in the displayed figures, all structures displayed were low pass filtered to 8 Å. Listerin difference density was fit into "snapshot V" of negative stain reconstructions of yeast Ltn1 (emd-2252, Fig. 6d) (Lyumkis et al., 2013). Rigid body fitting of eIF6-bound 60S (Fig. 6e) was performed with the crystal structure of *T. thermophila* 60S ribosome subunit in complex with eIF6 (molecule 2, PDB 4A17 and 4A19) (Klinge et al., 2011). Figures were visualized, analyzed, and generated using the UCSF Chimera package (Pettersen et al., 2004).

Supplemental References

Bai, X.-C., Fernandez, I.S., McMullan, G., and Scheres, S.H. (2013). Ribosome structures to near-atomic resolution from thirty thousand cryo-EM particles. Elife *2*, e00461–e00461.

Klinge, S., Voigts-Hoffmann, F., Leibundgut, M., Arpagaus, S., and Ban, N. (2011). Crystal structure of the eukaryotic 60S ribosomal subunit in complex with initiation factor 6. Science *334*, 941–948.
