# Supplementary material for: Reconstitution of a Minimal Ribosome-Associated Ubiquitination Pathway with Purified Factors
Source: Mol Cell. 2014 Sep 18;55(6):880–90. doi: 10.1016/j.molcel.2014.07.006 (PMC4175178; doi:10.1016/j.molcel.2014.07.006)
Supplement: Document S2. Article plus Supplemental Information [file mmc2.pdf]

CellPress

Molecular Cell
Article

# Reconstitution of a Minimal Ribosome-Associated Ubiquitination Pathway with Purified Factors

Sichen Shao[1] and Ramanujan S. Hegde[1,*]
[1]MRC Laboratory of Molecular Biology, Cambridge, CB2 0QH, UK
*Correspondence: rhegde@mrc-lmb.cam.ac.uk
http://dx.doi.org/10.1016/j.molcel.2014.07.006
This is an open access article under the CC BY license (http://creativecommons.org/licenses/by/3.0/).

## SUMMARY

**Ribosomes stalled on aberrant mRNAs engage quality control mechanisms that degrade the partially translated nascent polypeptide. Ubiquitination of the nascent protein is mediated by the E3 ligase Listerin via a mechanism involving ribosome subunit dissociation. Here, we reconstitute ribosome-associated ubiquitination with purified factors to define the minimal components and essential steps in this process. We find that the primary role of the ribosome splitting factors Hbs1, Pelota, and ABCE1 is to permit Listerin access to the nascent chain. Listerin alone can discriminate 60S- from 80S-nascent chain complexes to selectively ubiquitinate the former. Splitting factors can be bypassed by artificially removing the 40S subunit, suggesting that mere steric hindrance impedes Listerin recruitment. This was illustrated by a cryo-EM reconstruction of the 60S-Listerin complex that identifies a binding interface that clashes with the 40S ribosomal subunit. These results reveal the mechanistic logic of the core steps in a ribosome-associated quality control pathway.**

## INTRODUCTION

Cells constantly monitor the quality of their proteins and selectively destroy aberrant species. Failure of quality control can lead to misfolded protein accumulation, an event linked to numerous diseases from diabetes to neurodegeneration (Chiti and Dobson, 2006). Quality control is now appreciated to act at essentially every stage in the life of a protein (Wolff et al., 2014). The defining and decisive event in any quality control pathway is substrate detection and selective targeting for degradation. Delineating the mechanistic basis of these key decisions for each quality control pathway is an important goal.

A newly discovered set of quality control pathways acts at the ribosome on nascent polypeptides that have yet to complete synthesis. Ubiquitinated nascent chains were observed many years ago (Sato et al., 1998), and proof-of-principle experiments established the possibility of cotranslational degradation (Turner and Varshavsky, 2000). However, systematic demonstration of nascent chain ubiquitination on the ribosome has only recently been undertaken (Duttler et al., 2013; Wang et al., 2013), and the pathways are poorly understood. The best defined of these is a ribosome-associated quality control (RQC) pathway that degrades the partially synthesized protein products of translationally stalled ribosomes (Bengtson and Joazeiro, 2010; Brandman et al., 2012; Dimitrova et al., 2009).

This pathway was discovered from the observation in yeast that translation of a “nonstop” mRNA lacking a stop codon generates a protein product degraded by the proteasome (Ito-Harashima et al., 2007). Nascent protein degradation was linked to multiple modes of ribosomal stalling including mRNA truncation, secondary structure within the coding region, and translation of polybasic residues such as reading into the polyA tail (Dimitrova et al., 2009). Reporters for this degradation pathway led to the identification of Ltn1 as the E3 ubiquitin ligase required for nonstop protein ubiquitination at the ribosome (Bengtson and Joazeiro, 2010). Listerin, the mammalian homolog of Ltn1, causes neurodegeneration when mutated in mouse (Chu et al., 2009), highlighting the physiologic importance of RQC.

A combination of genetic and physical interaction studies in yeast identified additional components of this RQC pathway including Asc1, Hel2, Tae2, Rqc1, and Cdc48 (Brandman et al., 2012; Defenouillère et al., 2013). Asc1 and Hel2 appear to act early in the pathway and are needed for stalling of ribosomes at codons of polybasic residues (Brandman et al., 2012; Kuroha et al., 2010). Once stalled, a poorly understood set of events lead to nascent chain ubiquitination. Affinity purification of tagged Ltn1 copurified the 60S ribosomal subunit, Tae2, Rqc1, Cdc48, and ubiquitinated products presumed to represent nascent polypeptides (Brandman et al., 2012; Defenouillère et al., 2013). Affinity purification of either Tae2 or Rqc1 copurified the same 60S complex. Notably, 60S recruitment of Ltn1, Tae2, and Rqc1 was independent of the other two factors. By contrast, Cdc48 recruitment was dependent on Ltn1-mediated ubiquitination, Rqc1, and Tae2 (Brandman et al., 2012; Defenouillère et al., 2013). Additional functional studies of Cdc48 suggested that this AAA+ ATPase acts at a late step to extract ubiquitinated nascent chains from the ribosome for proteasomal degradation (Defenouillère et al., 2013; Verma et al., 2013).

While these studies in yeast have defined a number of components and suggested a basic framework for the RQC pathway, numerous mechanistic questions remain to be addressed. A major issue is the order of events leading to nascent chain ubiquitination: while the final RQC complex is observed on 60S subunits, their initial order of recruitment, their roles in generating

60S from 80S ribosomes, and the step at which ubiquitination is triggered are all debated. A second question is how stalled ribosomes are discriminated from translating ribosomes and what role any of these factors play in stall recognition. Third, the functions of individual components are mostly unclear. The best functional data are for Ltn1 in mediating nascent chain ubiquitination (Bengtson and Joazeiro, 2010; Brandman et al., 2012) and for Cdc48 in nascent chain extraction (Defenouillère et al., 2013; Verma et al., 2013). Finally, how protein quality control events relate to the mRNA surveillance pathways that degrade stall-inducing messages is not understood (Shoemaker and Green, 2012).

To begin addressing these and other mechanistic questions, we recently initiated efforts to develop an in vitro system that recapitulates at least some aspects of the RQC pathway (Shao et al., 2013). Using a mammalian translation extract, we demonstrated that stalled ribosome-nascent chains (RNCs) rely on Listerin for their ubiquitination. Listerin, which is normally soluble, was selectively recruited to stalled RNCs. As in yeast, both Listerin and ubiquitinated nascent chains were found predominantly on 60S ribosome subunits, which were the preferred targets of ubiquitination. This implicated a role for ribosome splitting in the RQC pathway.

Splitting of 80S ribosomes is mediated by the ATPase ABCE1 (Pisarev et al., 2010; Shoemaker and Green, 2011). Recruitment of ABCE1 to empty or stalled ribosomes requires the eRF1 and eRF3 homologs Pelota and Hbs1 (Becker et al., 2012; Pisareva et al., 2011). In the case of stalled RNCs, splitting generates a unique 60S-nascent chain-tRNA complex that was speculated to be recognized by RQC components for protein quality control (Rodrigo-Brenni and Hegde, 2012; Shao et al., 2013). Consistent with this, a GTPase-deficient Hbs1 inhibited ribosome splitting and impaired Listerin recruitment to and ubiquitination of stalled RNCs in vitro (Shao et al., 2013). In yeast, deletion of Dom34 (the Pelota homolog) stabilized the protein product of a nonstop mRNA to the same level as Ltn1 deletion or Cdc48 inactivation (Verma et al., 2013), although the basis of this effect was not analyzed further. Thus, while ribosome splitting is implicated in Listerin-mediated ubiquitination, the relationship between these two events remains mechanistically obscure.

In this study, we begin with the previously characterized RNC ubiquitination system in translation extracts and systematically dissect it down to purified factors. This reductionist strategy allowed us to define the minimal components for converting a stalled 80S-RNC into a polyubiquitinated 60S-RNC. The purified system enabled us to order the sequence of events, determine the roles played by the main factors, and determine a 60S-Listerin structure that explains the basis of 60S-selective ubiquitination. The establishment of a purified system for the central reactions in the RQC pathway should greatly facilitate detailed structural and mechanistic studies of these and downstream events.

## RESULTS

### Purification of Stalled 80S RNCs

The first requirement for fully reconstituting the RQC pathway is the preparation of a well-defined starting substrate. We therefore optimized in vitro translation conditions to purify stalled 80S RNC complexes (Figure 1A). Our model nascent chain (F-VHP-β; Figure 1A) contains an N-terminal 3X-tandem FLAG tag, the autonomously folding villin head piece (VHP) domain, and the unstructured cytosolic domain of Sec61β. Radiolabeled nascent F-VHP-β was translated in vitro in a reticulocyte lysate using a stop-codon-lacking transcript whose last triplet codes for valine. A ribosome reaching the end of this transcript will stall with an intact tRNA-linked nascent chain and initiate downstream steps in the RQC pathway (Figure 1A, left option). To preclude the initiation of this pathway, we added ~5-fold excess of a GTPase-deficient dominant negative Hbs1 (Hbs1-DN) 7 min after the start of translation, when ribosomes were still in the middle of the transcript. Upon continued elongation, Hbs1-DN should outcompete endogenous Hbs1 to prevent splitting of 80S ribosomes that reach the end of the transcript (Figure 1A, right option). Hbs1-DN inhibition was verified using a short F-β nascent chain reporter that "drops off" the ribosome upon splitting (Figures S1A and S1B available online).

F-VHP-β RNCs prepared with Hbs1-DN were then stripped of peripherally associated proteins (including Hbs1-DN) with high salt and affinity purified via the FLAG tag (Figure 1B). The high-salt wash effectively prevented various ribosome-associating factors from cosedimenting with RNCs, resulting in a purified RNC preparation that did not contain detectable amounts of Listerin, ABCE1, Hbs1, or Hbs1-DN (Figure 1C). The nascent chain remained attached to the tRNA, as evidenced by its shift with RNase treatment (Figure 1B). The small amount of tRNA loss seems to occur during gel electrophoresis as evidenced by efficient recovery of these products during purification (Figure 1B) and sucrose gradient sedimentation (Figure 1D). Coomassie staining of the purified preparation revealed the expected ribosomal protein profile (Figure 1B), and both subunits were present in their starting ratios as judged by immunoblotting (Figure 1C) and sedimentation of the radiolabeled nascent chain as an 80S particle (Figure 1D). Importantly, the nascent chain was not detectably ubiquitinated, indicating that it had not engaged the RQC pathway.

Purified 80S F-VHP-β RNCs were inert to the addition of recombinant E1, E2 (UbcH5a), ubiquitin, and energy (Figure 1E, lanes 1 and 3). This contrasts with the observed ubiquitination of a crude ribosome fraction of VHP-β RNCs isolated under physiologic conditions (Shao et al., 2013), indicating that the more rigorously purified RNCs translated in the presence of Hbs1-DN have lost key factors needed for nascent chain ubiquitination. Importantly, addition of cytosolic S-100 fraction to purified F-VHP-β RNCs generated a high-molecular weight smear that was verified to be polyubiquitination by ubiquitin pull-downs (Figure 1E, lanes 2 and 4). Like the previously characterized native RNC substrate (Shao et al., 2013), the affinity purified substrate was ubiquitinated rapidly (within 5 min) and highly processively (Figure S1C), resulting in almost all of the products migrating at the very top of the gel (Figures 1E and S1C). Sedimentation assays verified that the ubiquitinated products are ribosome associated, and ubiquitination was prevented if the F-VHP-β substrate was released from ribosomes with RNase prior to the reaction (data not shown). Thus, affinity purified 80S F-VHP-β RNC is a functional substrate of the RQC pathway poised at the step of a fully intact stalled ribosome.

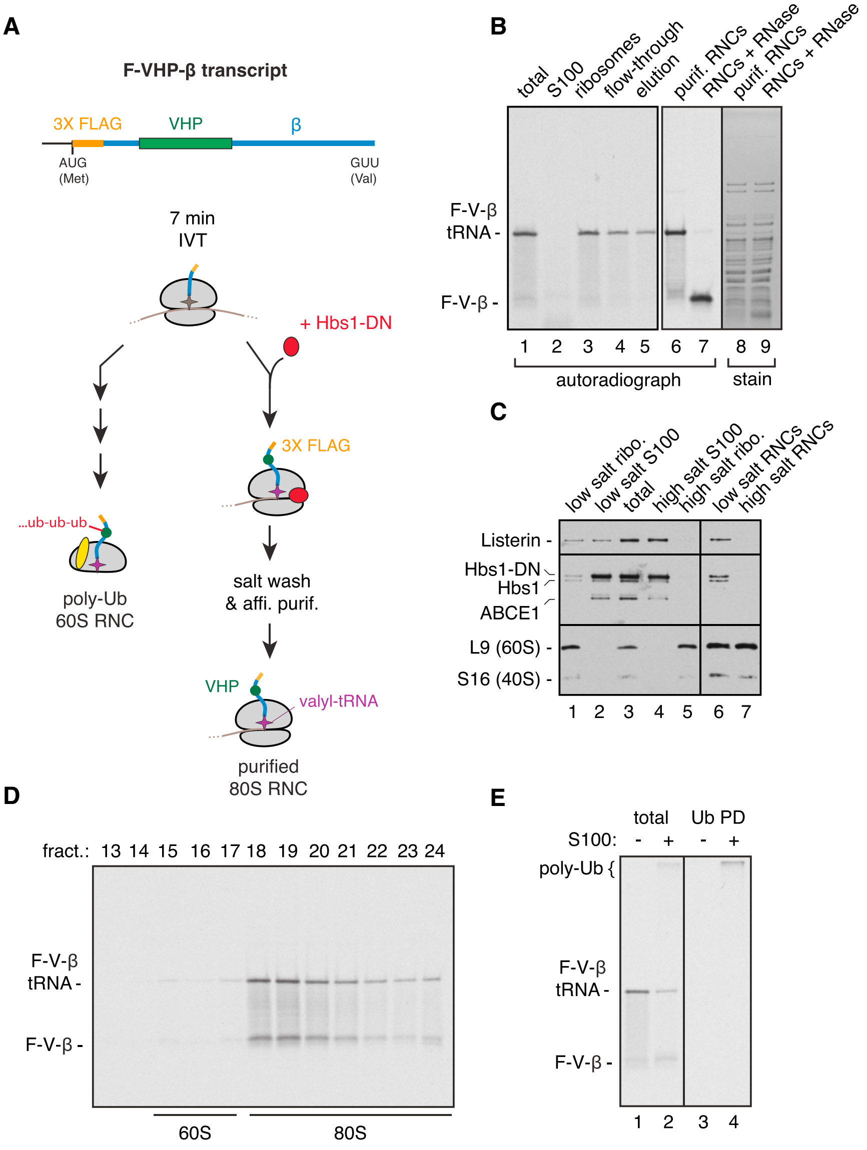


**Figure 1. Purified Stalled 80S RNCs Are Ubiquitinated In Vitro**

(A) Scheme for purifying stalled 80S RNCs housing the model substrate F-VHP-β.

(B) F-VHP-β was in vitro translated with $^{35}$S-methionine and purified by the scheme in (A). Fractions during the purification were analyzed by SDS-PAGE and autoradiography. The primary product contains an attached tRNA (F-V-β-tRNA), which is hydrolyzed by RNase to F-V-β (lane 7). Lanes 8 and 9 show Coomassie-blue-stained image of lanes 6 and 7, respectively.

(C) In vitro translation reaction of F-VHP-β (lane 3) was fractionated using physiologic or high-salt conditions (lanes 1–5) and affinity purified via the FLAG tag (lanes 6 and 7), as shown in (A). All fractions were immunoblotted for Listerin, Hbs1, ABCE1, and the ribosomal proteins L9 and S16. Endogenous Hbs1 and exogenously added Hbs1-DN are labeled.

(D) A 10%–30% sucrose gradient analysis of $^{35}$S-labeled F-VHP-β RNCs, purified as in (B), with positions of 60S and 80S indicated.

(E) Purified $^{35}$S-labeled F-VHP-β RNCs were subjected to ubiquitination reactions with or without S-100 derived from RRL. The total reaction products and ubiquitin pull-downs (Ub PD) via the His tag were analyzed by SDS-PAGE and autoradiography. The unmodified substrate (F-V-β-tRNA) and poly-ubiquitinated (poly-Ub) products are indicated. (See also Figure S1.)

## Uncoupling Ribosome Splitting and Ubiquitination of Stalled RNCs

With a defined substrate in hand, we combined fractionation studies with functional assays to identify the steps and factors involved in the mammalian RQC pathway. Since most, if not all, reactions in this pathway occur on ribosomes, we reasoned that a ribosome salt wash may contain many of the requisite factors. Indeed, a concentrated ribosome salt wash (Figure 2A) prepared from reticulocyte lysate or HEK293T cells was sufficient to mediate ubiquitination of purified 80S F-VHP-β RNCs when supplemented with E1 and E2 enzymes, ubiquitin, and energy (Figures 2B and S2A). The salt wash also contained ribosome splitting activity, which was evident by the ~50% nascent chain drop off using affinity purified 80S F-β RNCs as a substrate (Figure 2C).

To understand the relationship between the ubiquitination and splitting activities, we fractionated the active ribosome salt wash into three fractions via anion exchange chromatography (Figure S2B). The individual fractions had poor ribosome splitting and ubiquitination activities relative to the starting salt wash (Figures 2C and 2D), suggesting that both reactions may involve multiple components that get separated during the fractionation. Indeed, immunoblotting of these fractions revealed that the splitting factors Hbs1 and ABCE1 were efficiently separated away from each other and the E3 ligase Listerin (Figure S2B).

Since ribosome splitting is known to involve Pelota, Hbs1, and ABCE1 (Becker et al., 2011, 2012; Pisarev et al., 2010; Pisareva et al., 2011; Shoemaker et al., 2010), we tested these purified factors (Figure 2A) relative to the native salt wash and found them to be equally active (Figure 2C, bottom panel). Titration experiments showed that 50 nM splitting factors yielded the same degree of splitting as that seen in total translation extracts (data not shown). Using this optimized concentration of recombinant splitting factors, we retested the fractions for ubiquitination activity. Relative to the fractions in isolation, splitting activity now revealed ubiquitination activity specifically in fraction 2 at a similar level to either the starting salt wash (Figure 2D) or a mixture of fractions 1–3 (Figure S2C). These results show that ribosome splitting does not independently lead to ubiquitination, which requires additional factors. Conversely, the ubiquitination

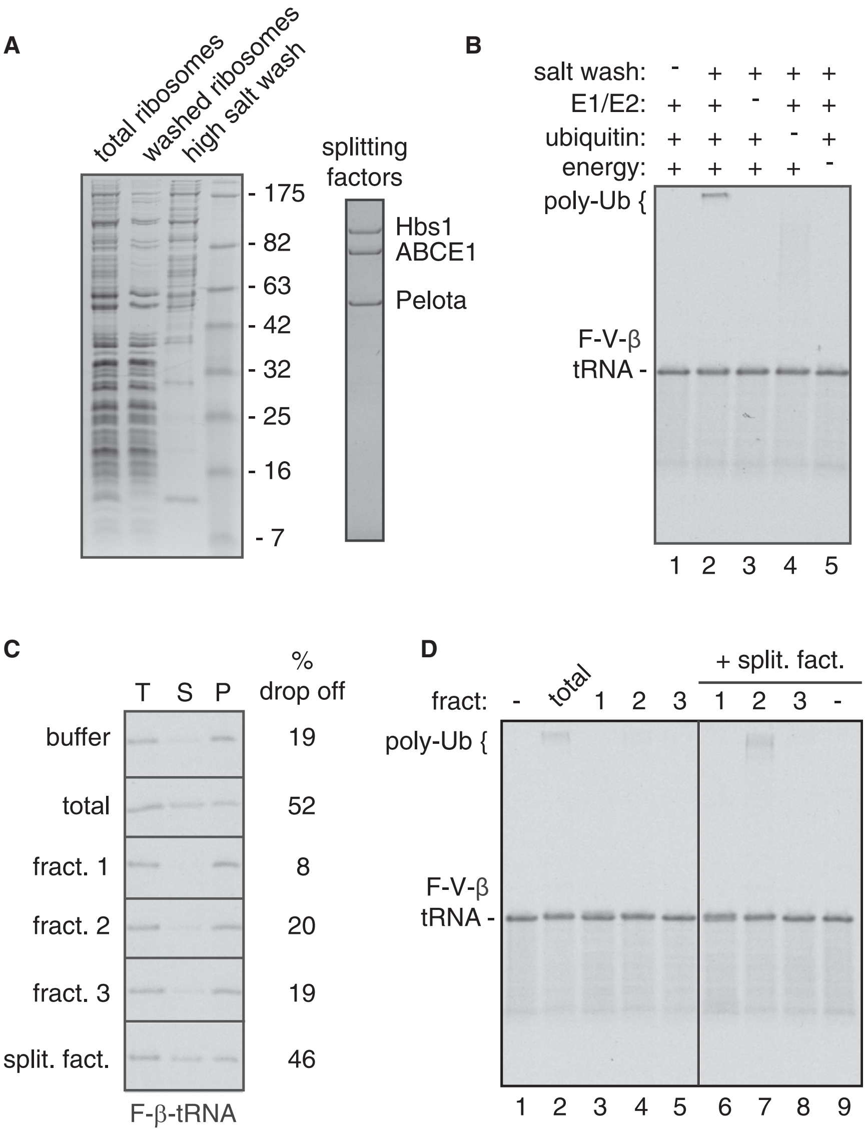


**Figure 2. Uncoupling Ribosome Splitting and Ubiquitination Activities**

(A) Coomassie-stained gels of fractions from a high salt extraction of native reticulocyte ribosomes (left) and purified splitting factors (right).

(B) Purified $^{35}$S-labeled 80S F-VHP-β RNCs were subjected to ubiquitination assays with the indicated components, and the total reaction products were analyzed by autoradiography.

(C) Drop-off assay to monitor ribosome splitting using purified $^{35}$S-labeled F-β RNCs. The total reaction (T), supernatant (S), and pellet (P) fractions were analyzed by autoradiography, and the relative amount of F-β-tRNA in the supernatant was quantified (% drop off).

(D) Purified $^{35}$S-labeled 80S F-VHP-β RNCs were subjected to ubiquitination assays with the indicated components, and the total reaction products analyzed by autoradiography. The unmodified substrate (F-V-β-tRNA) and poly-ubiquitinated (poly-Ub) products are indicated. (See also Figure S2.)

factors do not have appreciable splitting activity, but rely on splitting for ubiquitination. Importantly, all factors needed for ribosome splitting-dependent ubiquitination reside in fraction 2.

## Ribosome Splitting Precedes Listerin-Mediated Ubiquitination

In an attempt to understand why the splitting factors are required for RNC ubiquitination, we performed order-of-addition experiments. We found that maximal ubiquitination is only achieved when 80S RNCs are simultaneously incubated with ubiquitination factors (i.e., fraction 2) and splitting factors (Figure 3A, lane 3). In contrast, pretreating the RNCs with splitting factors before adding fraction 2 significantly diminishes ubiquitination (Figure 3A, lane 4). This suggests that ubiquitination works best when the machinery is present at the time of splitting. There are two general explanations for this result. First, it is possible that the splitting factors recruit the ubiquitination machinery to load it onto the 60S subunit at the time of splitting. Alternatively, 40S rebinding to the 60S after splitting could be a strong competing reaction to ubiquitination. In a native setting, this rebinding would be impeded because various initiation factors bind to free subunits at their interaction interface; but in our purified system, the high affinity between the subunits results in rapid reassociation.

To distinguish between these possibilities, we introduced the antiassociation factor eIF6 into our assays. This factor binds to the interface side of 60S subunits and prevents 40S association (Gartmann et al., 2010; Valenzuela et al., 1982). When eIF6 is included in the pretreatment reaction with splitting factors, fraction 2 becomes fully active for ubiquitination (Figure 3A, lane 5, compare to lane 4). Adding eIF6 at the same time as both fraction 2 and splitting factors does not further enhance ubiquitination (Figure 3A, lane 6), indicating that eIF6 does not have intrinsic stimulatory activity. These results suggest that the ubiquitination machinery recognizes 60S-RNCs but not 80S-RNCs. In this view, the splitting factors are acting primarily to reveal the substrate for ubiquitination, with 40S subunit rebinding serving as a potent (and presumably nonphysiologic) inhibitor.

To test this idea further and exclude another essential role for the splitting factors, we asked whether artificially removing 40S subunits from our 80S RNCs permits ubiquitination by fraction 2. To do this, we exploited the observation that at moderate magnesium concentrations the intersubunit interaction is sufficiently dynamic to allow 60S capture by high concentrations of eIF6 (Valenzuela et al., 1982) (see diagram, Figure 3B). We found that splitting factors could be effectively bypassed in this manner, leading to efficient ubiquitination by fraction 2 in the presence of eIF6 as magnesium concentrations were

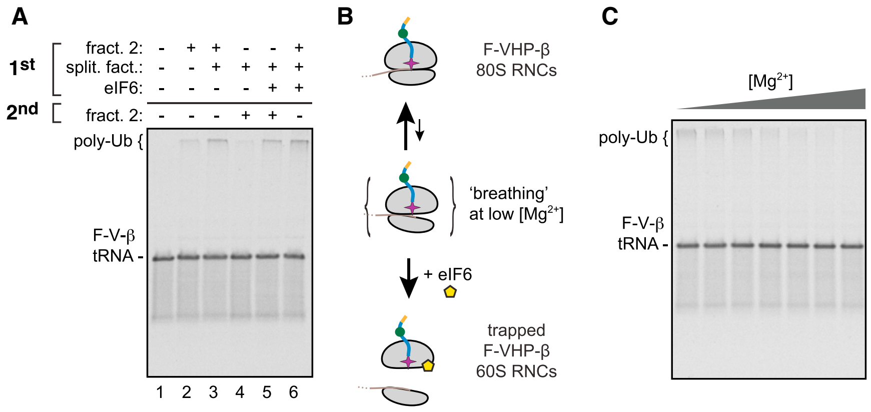


**Figure 3. Ribosome Splitting Precedes Ubiquitination**

(A) Purified $^{35}$S-labeled 80S F-VHP-β RNCs were subjected to a two-stage ubiquitination reaction with a preincubation (1$^{st}$) for 10 min with the indicated components, followed by another 10 min incubation (2$^{nd}$) without or with fraction 2. Total reaction products were analyzed by autoradiography.

(B) Schematic diagram illustrating the $Mg^{2+}$-dependent dissociation of 80S RNC by eIF6.

(C) Ubiquitination reactions of $^{35}$S-labeled 80S F-VHP-β RNCs at $Mg^{2+}$ concentrations from 3.4 mM to 14.4 mM. All reactions contained eIF6 and fraction 2 of the ribosome salt wash.

progressively lowered (Figure 3C). These observations argue that while the splitting factors may play a key role in identifying stalled ribosomes, they are not necessary for the specificity of the ubiquitination machinery for 60S over 80S RNCs; this preference is apparently intrinsic to the ubiquitination factors. Thus, ribosome splitting and ubiquitination do not need to be directly coordinated, as long as rejoining of the ribosome subunits is prevented, as would occur in vivo. We do not exclude the possibility that communication between the splitting and ubiquitination factors can improve efficiency, but our experiments have yet to provide evidence for this.

## Minimal Machinery for Stalled RNC Ubiquitination

To identify the factors for RNC ubiquitination via an unbiased strategy, we followed the splitting-dependent ubiquitination activity through several fractionation steps (see diagram, Figure S3A). Further separation of fraction 2 by cation exchange followed by sucrose gradient led to an enriched set of proteins with a single peak of activity (Figure 4A). No unexpected loss or heterogeneity of the activity was observed during fractionation, suggesting that the starting activity in the salt wash was fully represented in the final enriched fractions. Mass spectrometry on fractions 2–7 across the gradient provided an abundance profile for every protein, which was compared to the ubiquitination activity profile across the same fractions. This narrowed our focus to 18 cofractionating proteins (Figure S3C).

This list was compared to the set of proteins identified from a purified, native, and functional RQC complex (see Figure S3B for purification scheme). As a negative control, the native RQC sample was divided just before the affinity step, and one half was mildly digested with RNase to release the nascent chain while leaving the ribosome intact. Coomassie staining of the final native RQC preparation showed a set of proteins dependent on an intact tRNA, including the characteristic ribosomal protein profile (Figure 4B). All proteins from this experiment were identified by mass spectrometry (Table S1) and cross-checked with the candidates obtained from the functional fractionation strategy (Figure S3C).

Remarkably, the only E3 ligase common to both lists was Listerin (Figure S3C). Although four ligases were detected in the gradient fractions, only Listerin peaked in the same fractions as maximal ubiquitination activity (Figure 4C). Listerin was also the sole E3 ligase identified from the native RQC sample (Figure 4D). The only other protein we identified using this cross-validation approach was DHX29, a protein involved in 43S initiation complex formation (Pisareva et al., 2008). However, the large size of DHX29 (~150 kD) argued against its cofractionation with Listerin via an interaction, and nothing about it pointed to a role in ubiquitination. Thus, Listerin is the ligase for RNCs in this pathway, as had been expected from earlier genetic and biochemical analyses (Bengtson and Joazeiro, 2010; Brandman et al., 2012; Shao et al., 2013).

More surprising, however, was the absence of any other obvious functional factors revealed by the unbiased activity-based purification strategy. All other proteins that cofractionated with ubiquitination activity were absent from the native sample and were too large to have copurified via Listerin. While the mammalian homologs of yeast Tae2 (NEMF), Rqc1 (TCF25), and Cdc48 (p97) could be observed in the pull-down of either native RQC complexes or native Listerin-containing complexes (Figure 4D; data not shown), they were completely absent from the gradient fractions. This indicates that, in the absence of the ribosome, these factors are not in a stable physical complex with Listerin and, more importantly, that they are not strictly required for stalled RNC ubiquitination. These observations led us to strongly suspect that Listerin plus splitting factors would be sufficient for stalled RNC ubiquitination.

To test this, FLAG-tagged human Listerin was purified from HEK293T cells (Figure S4A) and verified to be enzymatically active in a RING domain-dependent manner (Figure S4B). Recombinant Listerin at physiological levels was able to recapitulate the level of purified RNC ubiquitination achieved by fraction 2 in the presence of splitting factors, E1, E2, ubiquitin, and energy (Figure 5A). A time course of ubiquitination under optimized conditions of all factors showed very rapid and highly processive nascent chain ubiquitination (Figure 5B), comparable to the reaction with complete lysate (Figure S1C). Like the endogenous activity, ubiquitination by purified Listerin was dependent on ribosome splitting factors, the E1 and E2 enzymes, the RING domain, and a ribosome-bound tRNA-associated substrate (Figure 5C).

At nonphysiologically high levels, Listerin could mediate a lower degree of slower and less processive RNC ubiquitination even in the absence of splitting factors (Figure S4C). This may

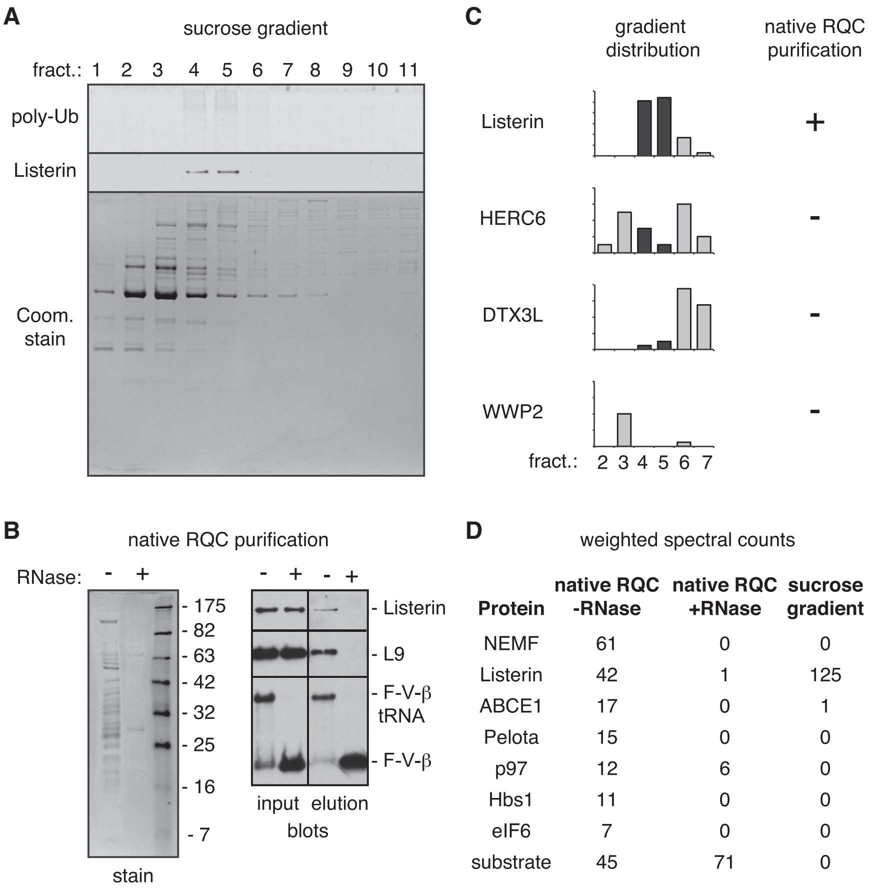


| Protein | native RQC -RNase | native RQC +RNase | sucrose gradient |
|---|---|---|---|
| NEMF | 61 | 0 | 0 |
| Listerin | 42 | 1 | 125 |
| ABCE1 | 17 | 0 | 1 |
| Pelota | 15 | 0 | 0 |
| p97 | 12 | 6 | 0 |
| Hbs1 | 11 | 0 | 0 |
| eIF6 | 7 | 0 | 0 |
| substrate | 45 | 71 | 0 |

**Figure 4. Identification of Ubiquitination Machinery**

(A) Fractions from the final sucrose gradient step of a purification scheme (see Figure S3A) were analyzed for RNC ubiquitination activity (top), Listerin immunoblot (middle), and Coomassie stain (bottom).

(B) Affinity purification of in vitro translated F-VHP-β RNCs via the scheme in Figure S3B. The products purified in the absence or presence of RNase were analyzed by Coomassie staining (left) and immunoblotting (right).

(C) Abundance profiles of E3 ubiquitin ligases across gradient fractions 2–7 from (A) identified by mass spectrometry. Fractions 4 and 5, where peak RNC ubiquitination activity was observed in (A), are highlighted in black.

(D) Weighted spectral counts of select proteins identified from mass spectrometry analysis of the native RQC purification from panel B (see Table S1 for the complete list). The spectral counts for these same proteins from the sucrose gradient fractions of (A) are also shown. (See also Figure S3 and Table S1.)

be due to either low affinity interactions between Listerin and 80S RNCs or some degree of spontaneous ribosome splitting to transiently expose 60S RNCs over time. To rigorously compare Listerin-mediated ubiquitination of 60S- versus 80S-RNCs, we staged the splitting and ubiquitination reactions (Figure S4D). Purified 80S RNCs were treated with splitting factors and eIF6 to generate a mixture of 60S and 80S nascent chain complexes. These complexes were separated on a sucrose gradient, and fractions containing each complex were presented to purified Listerin, E1, E2, ubiquitin, and ATP. Ubiquitination of 60S nascent chains was at least 10-fold more efficient than that of 80S nascent chains, whose ubiquitination was not convincingly detected (Figure S4E).

Additional experiments with 80S-RNCs showed that replacing Hbs1 with Hbs1-DN or adding subsets of splitting factors did not stimulate Listerin-mediated ubiquitination (data not shown). Thus, the entire set of wild-type splitting factors is needed for efficient Listerin-mediated ubiquitination. These results argue that ubiquitination is selective to 60S RNCs and that this specificity is apparently an intrinsic property of Listerin, since the splitting factor requirement can be bypassed by artificially removing the 40S subunit (Figures 3B and 3C). We conclude that the minimal machinery needed to processively ubiquitinate stalled nascent chains comprises of the E3 ligase Listerin; the splitting factors Hbs1, ABCE1, and Pelota; the E1 and E2 enzymes; ubiquitin; and energy.

## Listerin Specifically Recognizes 60S-Associated Substrates

A purified system for this part of the RQC pathway permitted us to examine the products of this well-defined reaction by cryo-EM. Concentrated 80S F-VHP-β RNCs were incubated with purified splitting factors, eIF6, and Listerin to assemble the complex of interest (Figure 6A). Parallel biochemical experiments verified that these complexes remained active for RNC ubiquitination (Figure S5A). The reactions were vitrified on EM grids and a data set containing 154,257 ribosome particles was collected. Initial particle classification using Relion (Scheres, 2012) identified a subset of 40,265 particles representing 60S ribosomes. Multiple rounds of subsequent classification of the 60S subset resolved two clear populations. The first, containing 11,710 particles, produced a map of the 60S subunit containing eIF6. The second, containing 9,148 particles, produced a 60S map containing eIF6 plus an additional density (Figure 6B; extra density in orange).

Because our reconstituted system contains only purified factors, and this additional density is not consistent with the known structures or positions of any of the splitting factors (Becker et al., 2011, 2012), we could reliably assign it to Listerin. Cryo-EM reconstructions of reactions in which Listerin was present at substantially lower concentrations failed to reveal the extra density (data not shown), further validating its assignment. The Listerin density tracks along the 60S subunit from near the 40S interface to near the exit tunnel (Figure 6B). Notably, the majority of the density does not appear to contact the ribosome, with relatively small interaction sites near either end. The clearest interaction is at the side closer to the exit tunnel, while the putative interaction near the 40S interface is poorly visible but close to eIF6.

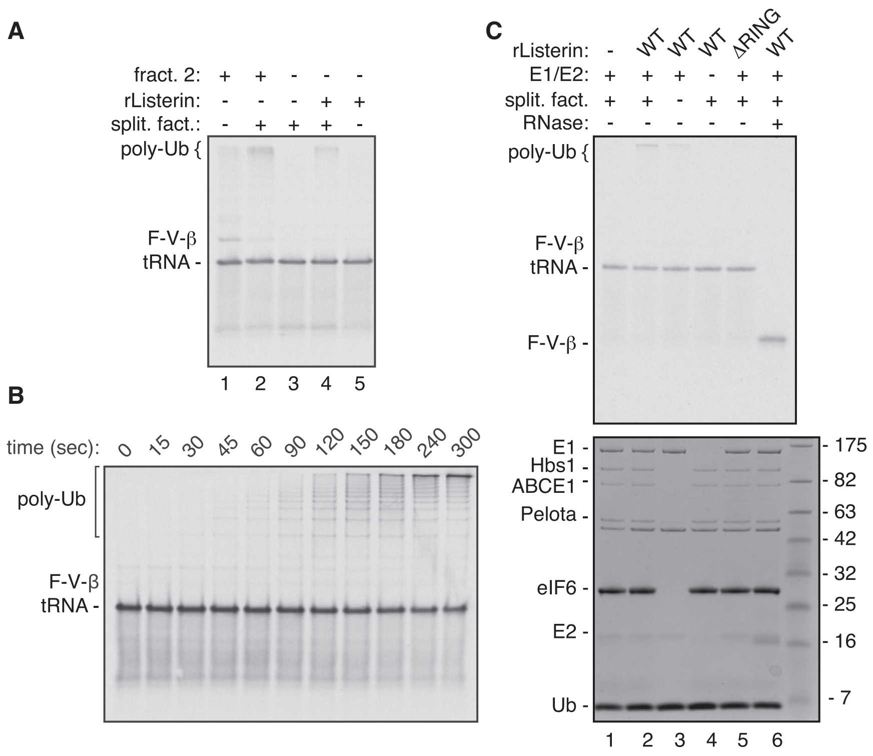


**Figure 5. Minimal Machinery for Stalled RNC Ubiquitination**

(A) Ubiquitination reactions of $^{35}$S-labeled 80S F-VHP-β RNCs with the indicated components comparing fraction 2 of the ribosome salt wash with purified Listerin at 0.3 nM, a concentration equivalent to that found in fraction 2.

(B) $^{35}$S-labeled 80S F-VHP-β RNCs were incubated with 75 nM E1, 250 nM UbcH5a, 10 μM ubiquitin, 50 nM splitting factors, 250 nM eIF6, 2.4 nM Listerin, and energy. Aliquots were removed at the indicated time points and analyzed by SDS-PAGE and autoradiography.

(C) Ubiquitination reactions of $^{35}$S-labeled 80S F-VHP-β RNCs with the indicated purified factors (Listerin at 12 nM). One reaction was pretreated with RNase (lane 6) before the reaction. Shown are the autoradiograph (top) and Coomassie stain (bottom) of the same gel. (See also Figure S4)

The overall resolution of this map was determined to be 4.9 Å, while local resolution (Figure S5B) ranged from ~4 Å for the 60S subunit and eIF6, to over 15 Å for parts of Listerin. Nevertheless, this initial view combined with biochemical and functional knowledge permits a number of provisional conclusions. The most unambiguous result is that this position of Listerin clashes extensively with the 40S subunit (Figure 6C, arrowheads). This agrees well with the requirement for subunit splitting and explains how Listerin is solely capable of discriminating 60S-substrates from 80S-nascent chains.

Definitively orienting Listerin within this density is hampered by relatively little prior structural knowledge of Listerin and the modest resolution of the current map. Nevertheless, it seems likely that the bulk of the strongly visible density corresponds to the numerous HEAT repeats predicted to form the central ~1,000 residues of Listerin (Lyumkis et al., 2013). The overall shape and volume of this density is consistent with similar HEAT repeat domains from other proteins such as exportin and the cullins. The Listerin density in our EM map matches the general shape of isolated Ltn1 visualized by negative stain EM (Figure 6D) (Lyumkis et al., 2013). Based on the orientation assigned to the negative stain map (Lyumkis et al., 2013), the side closer to the exit tunnel in our structure would be the C terminus. Our rigorous demonstration that Listerin directly ubiquitinates the nascent chain via its C-terminal RING domain is consistent with this assignment.

Since the RING domain is not actually necessary for Ltn1 binding to the yeast ribosome (Brandman et al., 2012), the major interaction with the ribosome at the exit tunnel side most likely occurs via the poorly characterized domain between the HEAT repeats and the RING domain. While the limited resolution of Listerin precludes detailed analysis of this interaction, the proteins nearby are L22, L31, and L19 (Figure 6E). Of these, L22 would be the strongest candidate for being directly involved since it is closest to the Listerin density. Future high-resolution structural information is needed to resolve this point. The RING domain has presumably contributed to the density that trails from L22 toward the exit tunnel, some of which could conceivably be the nascent chain. This would place the RING within ~40 Å of the nascent chain, which is well within the range of known RING-substrate distances (Deshaies and Joazeiro, 2009). Thus, Listerin binds to the 60S subunit such that 40S binding is mutually exclusive and the RING domain is near the exit tunnel.

## DISCUSSION

The normal translation cycle can be interrupted during elongation for any number of reasons ranging from physiologic pauses to pathologic stalls. Physiologic pauses are often linked to biosynthetic processes such as polypeptide folding (Komar, 2009), mRNA splicing (Rüegsegger et al., 2001; Yanagitani et al., 2011), frameshifting (Farabaugh, 1996), and protein localization (Walter and Blobel, 1981). By contrast, pathologic stalls are typically linked to degradative fates, including mRNA decay (Doma and Parker, 2006; Kuroha et al., 2010; Shoemaker and Green, 2012), polypeptide ubiquitination (Bengtson and Joazeiro, 2010; Shao et al., 2013), and ribosomal subunit degradation (Cole et al., 2009). Correctly linking the initiating event during translation to the appropriate downstream response is critical to cellular physiology, and defects in this coupling can lead to pathologic consequences (Chu et al., 2009; Rüegsegger et al., 2001; Yanagitani et al., 2011). The mechanisms by which the translation machinery interfaces with many of these downstream pathways remain enigmatic.

We have begun to investigate this issue for pathologic stalls that trigger the RQC pathway leading to nascent polypeptide ubiquitination and degradation. A critical question is what factors

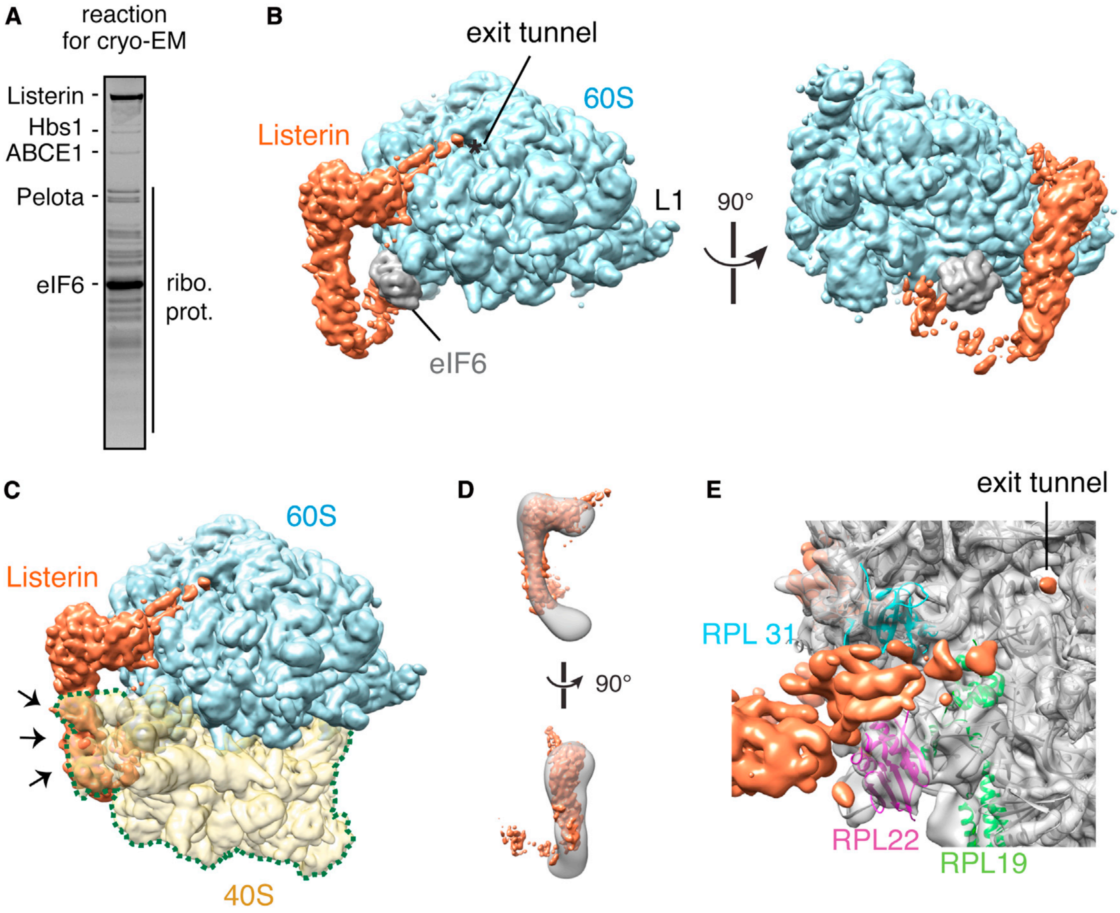


**Figure 6. Listerin Specifically Recognizes 60S Subunits**

(A) Coomassie stain of reaction used for cryo-EM analysis containing ~120 nM F-VHP-β RNCs, equimolar splitting factors (Hbs1, ABCE1, and Pelota), 1 mM ATP, 1 mM GTP, and a ~10-fold excess of purified Listerin and eIF6. Purified proteins and ribosomal proteins (ribo. prot.) are indicated.

(B) Cryo-EM reconstructions of the 60S ribosome subunit (cyan) with eIF6 (gray) and an additional density attributed to Listerin (orange). The ribosomal exit tunnel (asterisk) and L1 stalk are marked.

(C) Listerin binding to 60S ribosome subunits clashes (arrows) with where a 40S subunit (yellow) would bind on an 80S ribosome.

(D) Fitting of 60S-bound Listerin density into negative stain reconstructions of free Ltn1.

(E) Close-up of the region of Listerin interaction with the 60S ribosomal subunit near the exit tunnel with nearby proteins (RPL22, RPL19, and RPL31) indicated. (See also Figure S5.)

participate in the decisive step of discriminating stalled ribosomes to initiate the RQC pathway. The early contender from yeast studies was Ltn1, whose recruitment to ribosomes was thought to be triggered by stalling (Bengtson and Joazeiro, 2010). Somewhat puzzlingly, most Ltn1 was primarily associated with 60S subunits, not 80S ribosomes or polysomes (Bengtson and Joazeiro, 2010; Brandman et al., 2012; Shao et al., 2013). However, undegraded nascent chains in ΔLtn1 strains accumulated on 80S ribosomes, suggesting that Ltn1 may act on this species, which eventually gets disassembled to 60S subunits (Bengtson and Joazeiro, 2010; Defenouillère et al., 2013; Verma et al., 2013).

An alternative model was proposed based on experiments in a mammalian in vitro translation extract (Shao et al., 2013). Here, production of stalled nascent chains in reactions lacking the E2 enzyme led to both 60S and 80S complexes, with only the former containing Listerin and getting ubiquitinated when E1, E2, ubiquitin, and ATP were added. Furthermore, Hbs1 depletion or mutation partially inhibited 60S formation and nascent chain ubiquitination, suggesting that ribosome splitting precedes Listerin-mediated ubiquitination. This model was consistent with the observation that Ltn1 is found on 60S together with ubiquitinated nascent chains, but distinguishing between the two views remained a challenge.

Our reconstitution and structural studies provide three lines of evidence that decisively favor ribosome splitting preceding Listerin recruitment. First, purified 80S RNCs were not effectively ubiquitinated by either native or recombinant Listerin unless splitting factors were present in the reaction. Second, artificially splitting ribosomes by 60S trapping was sufficient to stimulate nascent chain ubiquitination. Third, the Listerin-60S structure shows extensive clashes with 40S, effectively precluding

interaction with 80S RNCs. We therefore conclude that ribosome splitting is upstream of Listerin recruitment and nascent chain ubiquitination. This suggests that splitting factors are a key element of stalled ribosome discrimination and initiation of the RQC pathway.

In our study, we split ribosomes using Pelota, Hbs1, and ABCE1, factors identified to recycle empty and stalled 80S ribosomes in earlier in vitro experiments (Pisareva et al., 2011). The physiologic targets of these factors are only now emerging and clearly include ribosomes at the end of truncated mRNAs (Guydosh and Green, 2014; Tsuboi et al., 2012). Because we employed a truncated mRNA in our experiments, the use of Pelota/Hbs1/ABCE1 is appropriate and physiologically relevant. Whether other types of stalls utilize the same recycling mechanism is not clear. It is possible that, depending on circumstances, factors in addition to or instead of Pelota/Hbs1/ABCE1 participate in splitting. For example, ribosome-associated factors such as Asc1/RACK1 (Brandman et al., 2012; Kuroha et al., 2010) and Hel2 (Brandman et al., 2012) seem to be important for stalling ribosomes at polybasic domains. Whether these factors are linked to the subsequent splitting reaction or involved in other types of stalls is not known. Similarly, initiation factors (Pisarev et al., 2007), release factors, or other RF3-like proteins such as Ski7 could also play a role in stall-induced splitting. The existence of multiple routes to ribosome recycling may explain why Dom34 and Hbs1 are not essential in yeast, why they seem to impact the RQC pathway in some but not other studies, and why they were not hits in genetic screens that found other RQC factors.

By contrast to this unresolved upstream step, the ligase that directly ubiquitinates the nascent chain would appear to be firmly settled. Earlier studies showed Ltn1 in yeast and Listerin in mammals as required for stalled nascent chain ubiquitination (Bengtson and Joazeiro, 2010; Brandman et al., 2012; Shao et al., 2013; Verma et al., 2013), strongly implicating this as the direct ligase. This expectation was rigorously validated in the current study, where both an unbiased activity-based purification strategy and a substrate-based coassociation strategy culminated with Listerin as the sole cofractionating E3 ligase. These results, together with our finding that recombinant Listerin can mediate ubiquitination at near-physiologic levels, establishes Listerin as the nascent chain E3 ligase for the RQC pathway.

The molecular basis of how Listerin finds its targets has been partially illuminated by our 60S-Listerin structure and addresses an otherwise puzzling issue: how can this ligase recognize its nascent chain target in only one of several potential contexts? A polypeptide in the 60S-NC complex should be efficiently targeted, while the identical polypeptide on an elongating or paused ribosome, free in solution, or as a mature protein must be scrupulously avoided. The answer lies in the fact that the ligase does not actually recognize its target, but rather the vessel housing that target. This is an emerging theme in quality control, where biosynthetic factors are increasingly found to act as adaptors between the polypeptide and a ligase (Hessa et al., 2011; Rodrigo-Brenni and Hegde, 2012).

For the RQC pathway, highly selective access is provided by removing the 40S subunit to expose regions occupied subsequently by Listerin. While this observation nicely explains Listerin specificity for 60S over 80S RNCs, it does not address why empty 60S subunits do not compete. Earlier experiments had suggested that such discrimination may involve the exposed tRNA of a 60S complex given that Listerin recruitment was more efficient when a nascent chain was present (Shao et al., 2013). Unfortunately, our current structure is poorly resolved in this region and does not address this issue.

It is possible that other RQC factors such as Tae2 or Rqc1 recognize and stabilize the tRNA. Indeed, we find that recombinant Listerin in our purified system is less stably associated with the 60S-NC complex than native Listerin in the crude salt wash (data not shown). Similarly, Ltn1 association with 60S subunits was reduced in ΔTae2 yeast strains (Defenouillère et al., 2013). It is therefore attractive to posit that, although not required for 60S-Listerin binding or nascent chain ubiquitination, Tae2 and/or Rqc1 stabilize this complex to improve efficiency or specificity of recognition. A stable complex may be important for preventing 40S rebinding and recruiting downstream factors such as Cdc48. Indeed, Cdc48 association with 60S complexes, but not ubiquitination per se, was dependent on Rqc1 (Brandman et al., 2012; Defenouillère et al., 2013). Analysis of these downstream steps remains an important goal that should be facilitated by the ability to fully reconstitute the preceding steps with purified factors.

Considering the roles provisionally assigned to the various factors from our and other studies, it would seem that the decisive and committed step of the RQC pathway is ribosome splitting. This would not only be irreversible due to loss of the 40S subunit and associated mRNA but also permits the ubiquitination machinery to efficiently access the nascent chain. This conclusion suggests that cotranslational ubiquitination (Duttler et al., 2013; Wang et al., 2013) should be mechanistically and conceptually distinguished from the RQC pathway analyzed here. While both involve ubiquitination at the ribosome, the RQC pathway is not cotranslational, as ubiquitination occurs after ribosome splitting via a ligase with 60S specificity. By contrast, cotranslational ubiquitination would necessarily involve polypeptides engaged in translation and therefore capable of being elongated (Wang et al., 2013). Since Listerin does not seem capable of binding 80S ribosomes, the mechanisms underlying cotranslational ubiquitination probably involve unidentified factors distinct from the RQC pathway. Thus, there appear to be multiple ribosome-associated pathways of quality control that warrant study in the future.

## EXPERIMENTAL PROCEDURES

### Reagents and General Biochemistry

Constructs encoding VHP-β, β, Hbs1, Hbs1-DN, Pelota, and eIF6 have been described (Shao et al., 2013). Standard procedures were used to generate tagged substrate constructs and expression constructs of WT and ΔRING Listerin and ABCE1. Rabbit polyclonal antibodies against Hbs1 and ABCE1 were raised with peptide antigens (Cambridge Research Biomedicals). All other antibody reagents and in vitro translation-based methods have been described (Hessa et al., 2011; Mariappan et al., 2010; Shao and Hegde, 2011; Shao et al., 2013; Sharma et al., 2010; Stefanovic and Hegde, 2007). Details can be found in Supplemental Experimental Procedures.

### Ubiquitination and Ribosome Splitting Assays

Ubiquitination reactions of F-VHP-β and splitting reactions of F-β 80S RNCs (5 nM) were at 32°C for 5–20 min with the components indicated in individual

figures. Final concentrations of purified components were as follows: 50 nM splitting factors, 250 nM eIF6, 75 nM E1, 250 nM of E2 (UbcH5a), 10 μM of His- or HA-tagged ubiquitin, and 0.3 nM to 50 nM Listerin, as indicated in the individual figure legends. Ubiquitination reactions were analyzed directly by SDS-PAGE or denatured and subjected to ubiquitin pull-downs. Splitting reactions were centrifuged in a TLA120.1 rotor at 70,000 rpm for 30 min and equal amounts of the total, supernatant, and pellet fractions analyzed by SDS-PAGE.

### Cryo-Electron Microscopy and Image Analysis

The reaction shown in Figure 6A was incubated for 15 min at 32°C and immediately vitrified on R2/2 Cu 400 mesh grids (Quantifoil) using an FEI Vitrobot. Automated data collection (EPU software, FEI) was conducted on a Titan Krios operated at 300 kV at 104,478× magnification with 1-s exposures ranging in defocus values from 2 to 3.5 μm. Particles were picked with e2boxer (Tang et al., 2007) and then classified and refined using Relion (Scheres, 2012). For comparisons in the displayed figures, all structures were low pass filtered to 8 Å. Figures were visualized and generated using Chimera (Pettersen et al., 2004).

## ACCESSION NUMBERS

The EM Data Bank accession numbers for the cryo-EM density maps reported in this study are 2702, 2703, and 2704.

## SUPPLEMENTAL INFORMATION

Supplemental Information includes five figures, one table, and Supplemental Experimental Procedures and can be found with this article online at http://dx.doi.org/10.1016/j.molcel.2014.07.006.

## ACKNOWLEDGMENTS

We thank Christos Savva for overseeing cryo-EM analysis, Xiaochen Bai and Felix Weis for help with image processing, and the other members of the LMB EM and scientific computing facilities for assistance. We thank Mark Skehel and the MS facility, Karina von der Malsburg for antibodies, Maryann Kivlen for constructs, Balaji Santhanam for sequence analysis, and members of the Hegde lab for discussions. This work was supported by the UK Medical Research Council (MC_UP_A022_1007 to R.S.H.), an MRC career development fellowship (S.S.), and a St. John's College Title A fellowship (S.S.).

Received: April 28, 2014
Revised: June 23, 2014
Accepted: July 10, 2014
Published: August 14, 2014

## REFERENCES

Becker, T., Armache, J.-P., Jarasch, A., Anger, A.M., Villa, E., Sieber, H., Motaal, B.A., Mielke, T., Berninghausen, O., and Beckmann, R. (2011). Structure of the no-go mRNA decay complex Dom34-Hbs1 bound to a stalled 80S ribosome. Nat. Struct. Mol. Biol. *18*, 715–720.

Becker, T., Franckenberg, S., Wickles, S., Shoemaker, C.J., Anger, A.M., Armache, J.-P., Sieber, H., Ungewickell, C., Berninghausen, O., Daberkow, I., et al. (2012). Structural basis of highly conserved ribosome recycling in eukaryotes and archaea. Nature *482*, 501–506.

Bengtson, M.H., and Joazeiro, C.A.P. (2010). Role of a ribosome-associated E3 ubiquitin ligase in protein quality control. Nature *467*, 470–473.

Brandman, O., Stewart-Ornstein, J., Wong, D., Larson, A., Williams, C.C., Li, G.-W., Zhou, S., King, D., Shen, P.S., Weibezahn, J., et al. (2012). A ribosome-bound quality control complex triggers degradation of nascent peptides and signals translation stress. Cell *151*, 1042–1054.

Chiti, F., and Dobson, C.M. (2006). Protein misfolding, functional amyloid, and human disease. Annu. Rev. Biochem. *75*, 333–366.

Chu, J., Hong, N.A., Masuda, C.A., Jenkins, B.V., Nelms, K.A., Goodnow, C.C., Glynne, R.J., Wu, H., Masliah, E., Joazeiro, C.A.P., and Kay, S.A. (2009). A mouse forward genetics screen identifies LISTERIN as an E3 ubiquitin ligase involved in neurodegeneration. Proc. Natl. Acad. Sci. USA *106*, 2097–2103.

Cole, S.E., LaRiviere, F.J., Merrikh, C.N., and Moore, M.J. (2009). A convergence of rRNA and mRNA quality control pathways revealed by mechanistic analysis of nonfunctional rRNA decay. Mol. Cell *34*, 440–450.

Defenouillère, Q., Yao, Y., Mouaikel, J., Namane, A., Galopier, A., Decourty, L., Doyen, A., Malabat, C., Saveanu, C., Jacquier, A., and Fromont-Racine, M. (2013). Cdc48-associated complex bound to 60S particles is required for the clearance of aberrant translation products. Proc. Natl. Acad. Sci. USA *110*, 5046–5051.

Deshaies, R.J., and Joazeiro, C.A. (2009). RING domain E3 ubiquitin ligases. Annu. Rev. Biochem. *78*, 399–434.

Dimitrova, L.N., Kuroha, K., Tatematsu, T., and Inada, T. (2009). Nascent peptide-dependent translation arrest leads to Not4p-mediated protein degradation by the proteasome. J. Biol. Chem. *284*, 10343–10352.

Doma, M.K., and Parker, R. (2006). Endonucleolytic cleavage of eukaryotic mRNAs with stalls in translation elongation. Nature *440*, 561–564.

Duttler, S., Pechmann, S., and Frydman, J. (2013). Principles of cotranslational ubiquitination and quality control at the ribosome. Mol. Cell *50*, 379–393.

Farabaugh, P.J. (1996). Programmed translational frameshifting. Annu. Rev. Genet. *30*, 507–528.

Gartmann, M., Blau, M., Armache, J.-P., Mielke, T., Topf, M., and Beckmann, R. (2010). Mechanism of eIF6-mediated inhibition of ribosomal subunit joining. J. Biol. Chem. *285*, 14848–14851.

Guydosh, N.R., and Green, R. (2014). Dom34 rescues ribosomes in 3′ untranslated regions. Cell *156*, 950–962.

Hessa, T., Sharma, A., Mariappan, M., Eshleman, H.D., Gutierrez, E., and Hegde, R.S. (2011). Protein targeting and degradation are coupled for elimination of mislocalized proteins. Nature *475*, 394–397.

Ito-Harashima, S., Kuroha, K., Tatematsu, T., and Inada, T. (2007). Translation of the poly(A) tail plays crucial roles in nonstop mRNA surveillance via translation repression and protein destabilization by proteasome in yeast. Genes Dev. *21*, 519–524.

Komar, A.A. (2009). A pause for thought along the co-translational folding pathway. Trends Biochem. Sci. *34*, 16–24.

Kuroha, K., Akamatsu, M., Dimitrova, L., Ito, T., Kato, Y., Shirahige, K., and Inada, T. (2010). Receptor for activated C kinase 1 stimulates nascent polypeptide-dependent translation arrest. EMBO Rep. *11*, 956–961.

Lyumkis, D., Doamekpor, S.K., Bengtson, M.H., Lee, J.-W., Toro, T.B., Petroski, M.D., Lima, C.D., Potter, C.S., Carragher, B., and Joazeiro, C.A.P. (2013). Single-particle EM reveals extensive conformational variability of the Ltn1 E3 ligase. Proc. Natl. Acad. Sci. USA *110*, 1702–1707.

Mariappan, M., Li, X., Stefanovic, S., Sharma, A., Mateja, A., Keenan, R.J., and Hegde, R.S. (2010). A ribosome-associating factor chaperones tail-anchored membrane proteins. Nature *466*, 1120–1124.

Pettersen, E.F., Goddard, T.D., Huang, C.C., Couch, G.S., Greenblatt, D.M., Meng, E.C., and Ferrin, T.E. (2004). UCSF Chimera—a visualization system for exploratory research and analysis. J. Comput. Chem. *25*, 1605–1612.

Pisarev, A.V., Hellen, C.U.T., and Pestova, T.V. (2007). Recycling of eukaryotic posttermination ribosomal complexes. Cell *131*, 286–299.

Pisarev, A.V., Skabkin, M.A., Pisareva, V.P., Skabkina, O.V., Rakotondrafara, A.M., Hentze, M.W., Hellen, C.U.T., and Pestova, T.V. (2010). The role of ABCE1 in eukaryotic posttermination ribosomal recycling. Mol. Cell *37*, 196–210.

Pisareva, V.P., Pisarev, A.V., Komar, A.A., Hellen, C.U.T., and Pestova, T.V. (2008). Translation initiation on mammalian mRNAs with structured 5'UTRs requires DExH-box protein DHX29. Cell *135*, 1237–1250.

Pisareva, V.P., Skabkin, M.A., Hellen, C.U.T., Pestova, T.V., and Pisarev, A.V. (2011). Dissociation by Pelota, Hbs1 and ABCE1 of mammalian vacant 80S ribosomes and stalled elongation complexes. EMBO J. *30*, 1804–1817.

Rodrigo-Brenni, M.C., and Hegde, R.S. (2012). Design principles of protein biosynthesis-coupled quality control. Dev. Cell *23*, 896–907.

Rüegsegger, U., Leber, J.H., and Walter, P. (2001). Block of HAC1 mRNA translation by long-range base pairing is released by cytoplasmic splicing upon induction of the unfolded protein response. Cell *107*, 103–114.

Sato, S., Ward, C.L., and Kopito, R.R. (1998). Cotranslational ubiquitination of cystic fibrosis transmembrane conductance regulator in vitro. J. Biol. Chem. *273*, 7189–7192.

Scheres, S.H.W. (2012). RELION: implementation of a Bayesian approach to cryo-EM structure determination. J. Struct. Biol. *180*, 519–530.

Shao, S., and Hegde, R.S. (2011). A calmodulin-dependent translocation pathway for small secretory proteins. Cell *147*, 1576–1588.

Shao, S., von der Malsburg, K., and Hegde, R.S. (2013). Listerin-dependent nascent protein ubiquitination relies on ribosome subunit dissociation. Mol. Cell *50*, 637–648.

Sharma, A., Mariappan, M., Appathurai, S., and Hegde, R.S. (2010). In vitro dissection of protein translocation into the mammalian endoplasmic reticulum. Methods Mol. Biol. *619*, 339–363.

Shoemaker, C.J., and Green, R. (2011). Kinetic analysis reveals the ordered coupling of translation termination and ribosome recycling in yeast. Proc. Natl. Acad. Sci. USA *108*, E1392–E1398.

Shoemaker, C.J., and Green, R. (2012). Translation drives mRNA quality control. Nat. Struct. Mol. Biol. *19*, 594–601.

Shoemaker, C.J., Eyler, D.E., and Green, R. (2010). Dom34:Hbs1 promotes subunit dissociation and peptidyl-tRNA drop-off to initiate no-go decay. Science *330*, 369–372.

Stefanovic, S., and Hegde, R.S. (2007). Identification of a targeting factor for posttranslational membrane protein insertion into the ER. Cell *128*, 1147–1159.

Tang, G., Peng, L., Baldwin, P.R., Mann, D.S., Jiang, W., Rees, I., and Ludtke, S.J. (2007). EMAN2: an extensible image processing suite for electron microscopy. J. Struct. Biol. *157*, 38–46.

Tsuboi, T., Kuroha, K., Kudo, K., Makino, S., Inoue, E., Kashima, I., and Inada, T. (2012). Dom34:hbs1 plays a general role in quality-control systems by dissociation of a stalled ribosome at the 3′ end of aberrant mRNA. Mol. Cell *46*, 518–529.

Turner, G.C., and Varshavsky, A. (2000). Detecting and measuring cotranslational protein degradation in vivo. Science *289*, 2117–2120.

Valenzuela, D.M., Chaudhuri, A., and Maitra, U. (1982). Eukaryotic ribosomal subunit anti-association activity of calf liver is contained in a single polypeptide chain protein of Mr = 25,500 (eukaryotic initiation factor 6). J. Biol. Chem. *257*, 7712–7719.

Verma, R., Oania, R.S., Kolawa, N.J., and Deshaies, R.J. (2013). Cdc48/p97 promotes degradation of aberrant nascent polypeptides bound to the ribosome. Elife (Cambridge) *2*, e00308–e00308.

Walter, P., and Blobel, G. (1981). Translocation of proteins across the endoplasmic reticulum III. Signal recognition protein (SRP) causes signal sequence-dependent and site-specific arrest of chain elongation that is released by microsomal membranes. J. Cell Biol. *91*, 557–561.

Wang, F., Durfee, L.A., and Huibregtse, J.M. (2013). A cotranslational ubiquitination pathway for quality control of misfolded proteins. Mol. Cell *50*, 368–378.

Wolff, S., Weissman, J.S., and Dillin, A. (2014). Differential scales of protein quality control. Cell *157*, 52–64.

Yanagitani, K., Kimata, Y., Kadokura, H., and Kohno, K. (2011). Translational pausing ensures membrane targeting and cytoplasmic splicing of XBP1u mRNA. Science *331*, 586–589.

Molecular Cell, Volume *55*

## Supplemental Information

# Reconstitution of a Minimal Ribosome-Associated Ubiquitination Pathway with Purified Factors

**Sichen Shao and Ramanujan S. Hegde**

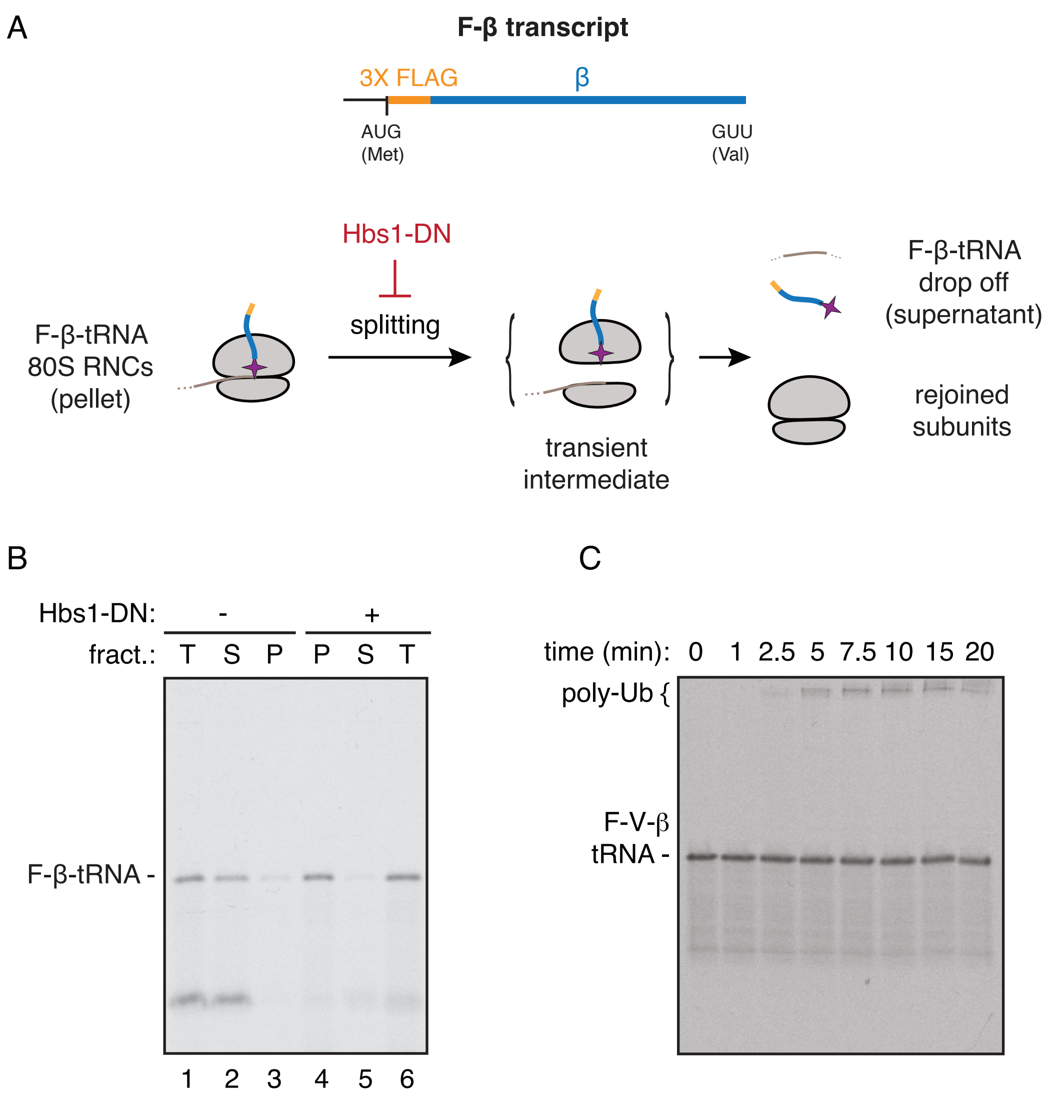


**Fig. S1** (Related to Fig. 1) (A) Schematic diagram showing the principle of the drop off assay to monitor ribosome splitting. The substrate is a stop codon-less model substrate (F-β, top) containing an N-terminal 3X FLAG tag (orange) followed by the unstructured region of Sec61β (blue) and ending in a valine codon (GUU). In this assay, stalled F-β ribosome-nascent chains (RNCs) are stably ribosome-associated as long as the tRNA is intact. Ribosome splitting would transiently allow the short nascent chain-tRNA to "back-slide" out of the 60S subunit before 40S re-joining can occur. Centrifugation can distinguish drop off products (supernatant) from 80S- or 60S-associated products (pellet). Thus, an intact tRNA-attached nascent chain in the supernatant can only arise as a consequence of ribosome splitting. A dominant negative version of the splitting factor Hbs1 (Hbs1-DN) should inhibit splitting, thus preventing drop off of F-β-tRNA. (B) Drop off assay of total translation reactions (T) of F-β with or without Hbs1-DN. In the absence of Hbs1-DN, the majority of F-β-tRNA is found in the supernatant (S), indicating efficient ribosome splitting activity (lanes 1-3). Hbs1-DN effectively inhibits splitting, causing all of F-β-tRNA to remain associated with ribosomal pellets (P, lanes 4-6). (C) Autoradiograph of a timecourse of purified $^{35}$S labeled 80S F-VHP-β RNC ubiquitination (see Fig. 1a) incubated with of 75 nM E1, 250 nM E2, 10 µM tagged ubiquitin, energy, and cytosolic S-100. F-V-β-tRNA denotes primary tRNA-associated nascent chain product, and poly-Ub denotes ubiquitinated substrate.

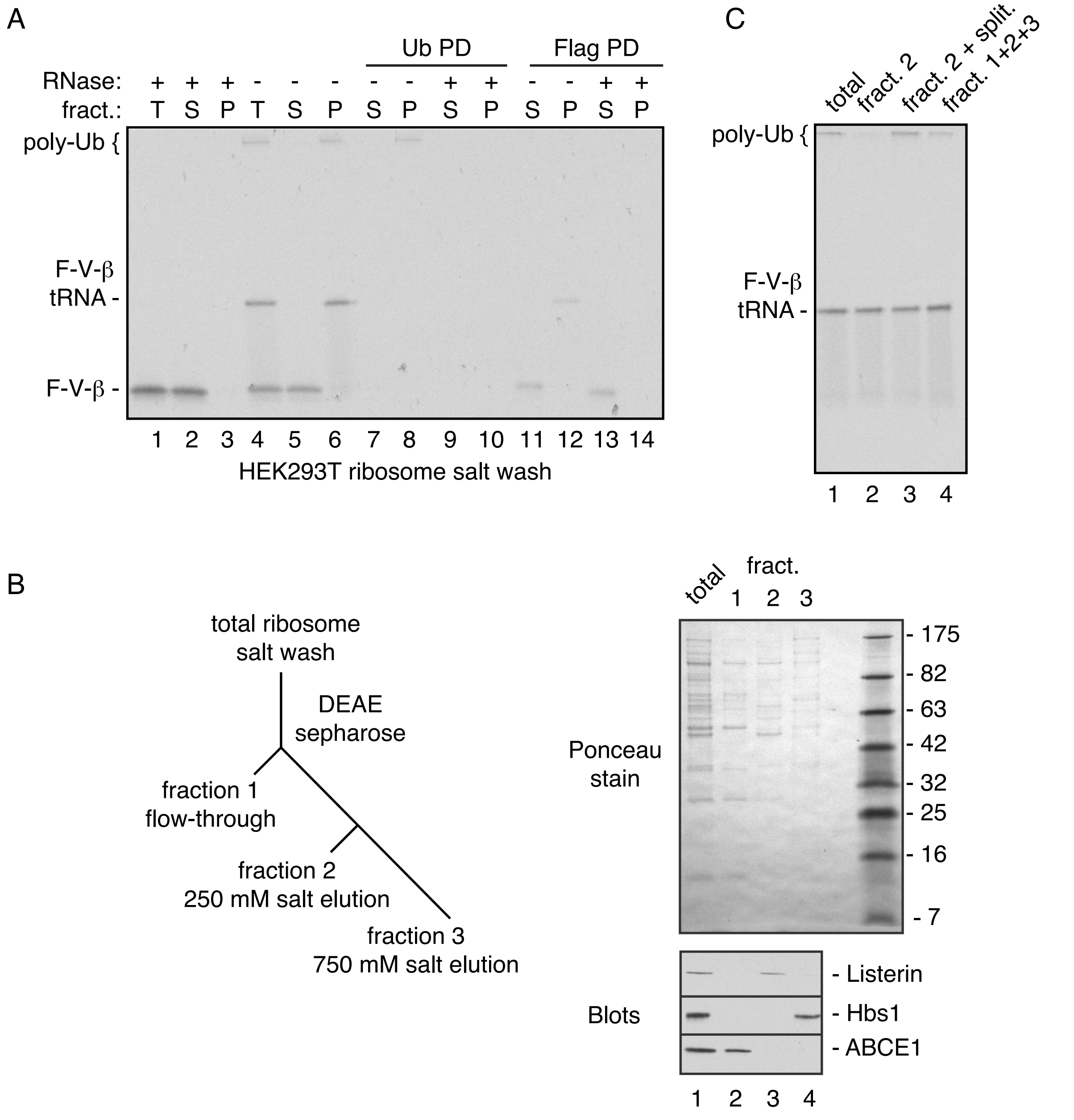


**Fig. S2** (Related to Fig. 2) (A) The ribosome salt wash from HEK293T cells can mediate poly-ubiquitination (poly-Ub) of purified $^{35}$S labeled F-VHP-β RNCs. Ubiquitination is dependent on an intact tRNA (compare lanes 1-3 of reactions conducted after RNase treatment to lanes 4-6). Total reactions (T) were centrifuged to isolate soluble factors (S) and ribosomal pellets (P), showing that ubiquitinated products remain ribosome-associated (lanes 6, 8, and 12). Ubiquitinated products can be distinguished by pulldowns against tagged ubiquitin (lanes 7-10). (B) Fractionation procedure to generate three fractions from total ribosome salt wash via anion exchange chromatography (left). The total ribosome salt wash (lane 1) and the individual fractions (lanes 2-4) were analyzed by SDS-PAGE and transferred to nitrocellulose membranes for immunoblotting (right). Ponceau stain of the membrane (top) demonstrates distinct protein profiles between the three fractions. Immunoblotting (bottom) reveals that the splitting factors Hbs1 and ABCE1 are fractionated away from each other and from the E3 ligase Listerin during this process. (C) Purified 80S F-VHP-β was incubated with 75 nM E1, 250 nM E2, 10 µM tagged ubiquitin, energy, and either total ribosome salt wash (lane 1), fraction 2 (lane 2), fraction 2 with recombinant splitting factors (lane 3), or a combination of fractions 1, 2, and 3 (lane 4). Reactions were directly analyzed by SDS-PAGE and autoradiography to visualize the tRNA-associated F-VHP-β nascent chains (F-V-β-tRNA) and ubiquitinated products (poly-Ub). Fraction 2 can mediate ubiquitination in the presence of recombinant splitting factors (lane 3) to the levels obtained with total ribosome salt wash (lane 1) or with the combination of all three fractions of the salt wash (lane 4).

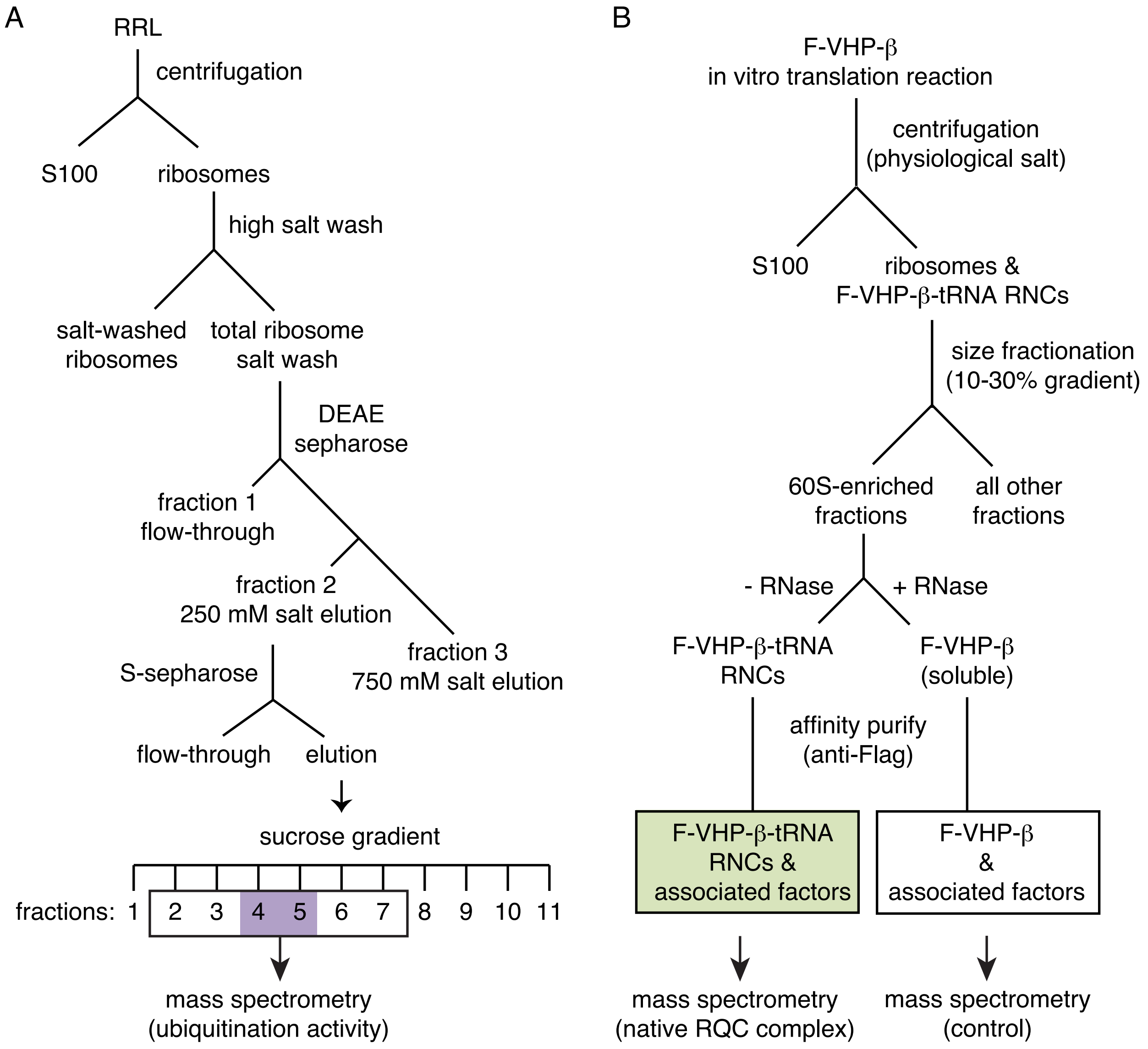


C

| | **2** | **3** | **4** | **5** | **6** | **7** | **Total** | **in Native** |
|---|---|---|---|---|---|---|---|---|
| DHX29 | 5 | 10 | 77 | 50 | 27 | 12 | 181 | 12 |
| Listerin | 0 | 0 | 51 | 54 | 17 | 3 | 125 | 42 |
| CKAP5 | 2 | 14 | 55 | 32 | 8 | 1 | 112 | 0 |
| ARD1 | 3 | 0 | 31 | 31 | 3 | 7 | 75 | 0 |
| SAMD9L | 0 | 0 | 23 | 26 | 12 | 2 | 63 | 0 |
| SMC1A | 0 | 0 | 21 | 25 | 10 | 7 | 63 | 0 |
| PLCB3 | 0 | 6 | 28 | 17 | 7 | 1 | 59 | 0 |
| ECM29 | 0 | 0 | 24 | 24 | 6 | 1 | 55 | 0 |
| SAMD9 | 0 | 0 | 18 | 23 | 10 | 2 | 53 | 0 |
| NAA20 | 0 | 3 | 8 | 13 | 1 | 0 | 25 | 0 |
| XRN1 | 0 | 0 | 6 | 6 | 0 | 0 | 12 | 0 |
| SBF1 | 0 | 0 | 5 | 2 | 0 | 1 | 8 | 0 |
| RPTOR | 0 | 0 | 4 | 3 | 0 | 0 | 7 | 0 |
| CBP | 0 | 0 | 4 | 1 | 0 | 0 | 5 | 0 |
| PARVB | 0 | 0 | 2 | 2 | 0 | 0 | 4 | 0 |
| CTPS2 | 0 | 0 | 2 | 3 | 0 | 0 | 5 | 0 |
| FACI | 0 | 0 | 2 | 1 | 0 | 0 | 3 | 0 |
| EHD4 | 0 | 0 | 2 | 1 | 0 | 0 | 3 | 0 |

**Fig. S3** (Related to Fig. 4) (A) Fractionation scheme for partial purification of splitting-dependent RNC ubiquitination activity. Rabbit reticulocyte lysate (RRL) was centrifuged to isolate native ribosomes and S100. A total salt wash was prepared from the ribosomes by adjusting salt levels to 750 mM KAc and centrifugation through a high salt 0.5 M sucrose cushion. The ribosome salt wash was desalted and then fractionated by anion exchange chromatography (DEAE-sepharose) into the flow-through (fraction 1) and two step elutions (fractions 2-3). Fraction 2, which contains ribosome splitting-dependent ubiquitination activity (Fig. 2b), was further fractionated by cation exchange (S-sepharose). The salt elution, which contains the ubiquitination activity, was further separated on a 5-25% sucrose gradient into 11 fractions. Of these, fractions 4 and 5 (purple) were identified to have splitting-dependent ubiquitination activity (Fig. 4a). Mass spectrometry analysis was used to identify the proteins present in gradient fractions 2-7 (see panel C). (B) Affinity purification scheme to isolate native RNC complexes containing RQC components. F-VHP-β RNCs were produced by in vitro translation (without Hbs1-DN) and pelleted via centrifugation. The ribosomal pellet was resuspended and subject to size fractionation on a 10-30% sucrose gradient. The fractions enriched for 60S ribosomal subunits were pooled and divided in two aliquots. One aliquot was treated with RNase while the other was left untreated. Both samples were then subject to affinity purification of the RNCs via the N-terminal 3X FLAG tag on the nascent chain. The elutions of the F-VHP-β RNCs and the control sample treated with RNase were analyzed by Coomassie staining (Fig. 4b) and mass spectrometry to identify associating proteins (Table S1). (C) Table of all protein identifications across the gradient fractions from panel A whose weighted spectral counts peak in the same fractions as ubiquitination activity (purple). Spectral counts for these same proteins from the native RQC sample prepared in panel B are also shown (green).

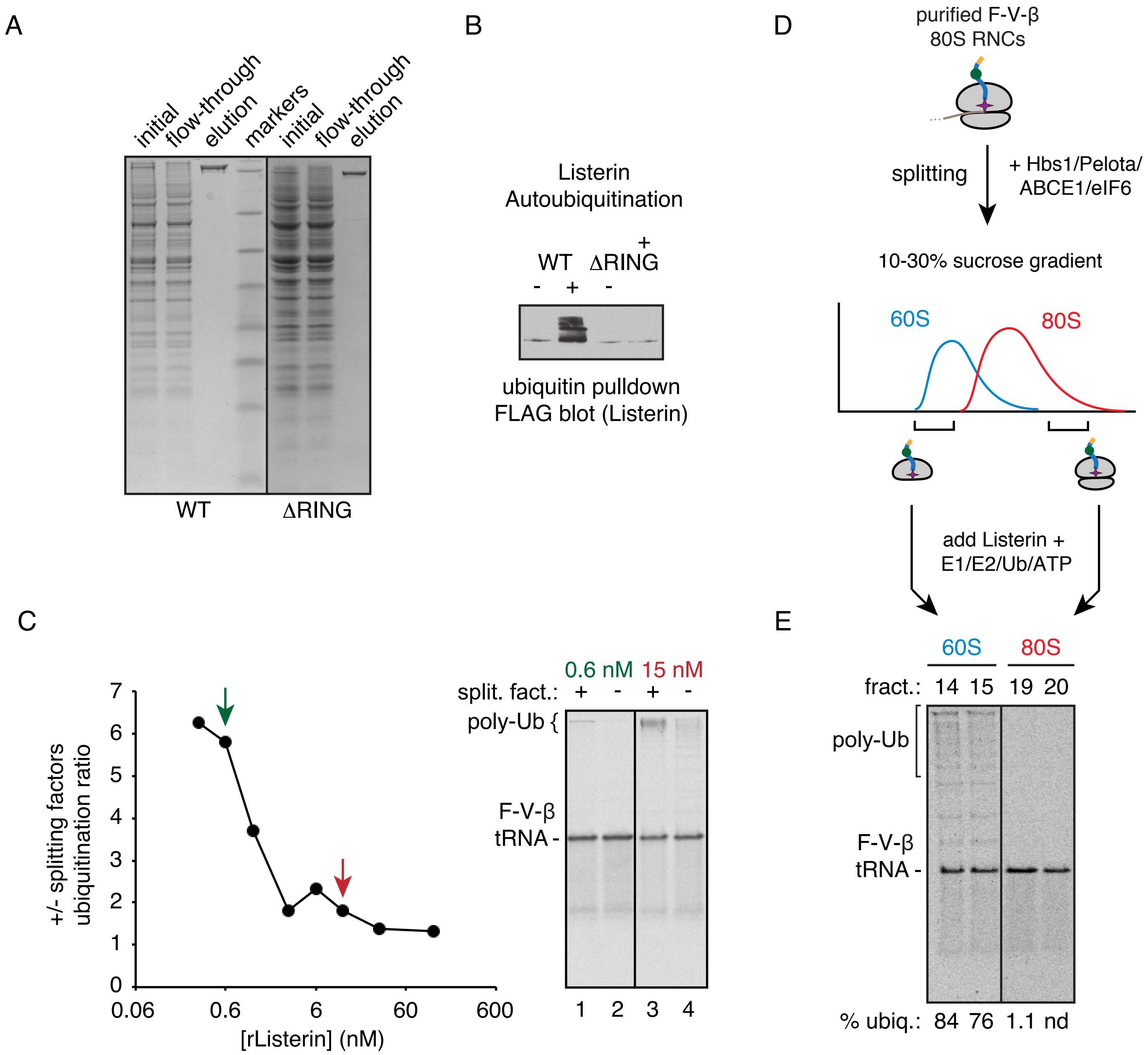


**Fig. S4** (Related to Fig. 5) (A) Coomassie stained gel of Flag-tagged wildtype (WT) and ΔRING Listerin purified from transiently transfected HEK293T cells. (B) Purified WT or ΔRING Listerin was incubated with 75 nM E1, 250 nM E2 (UbcH5a), 10 µM tagged ubiquitin, and energy. Ubiquitin pulldowns from these reactions were analyzed by anti-FLAG immunoblot to detect Listerin. Note that Listerin is autoubiquitinated in a reaction that is dependent on the E1 and E2 enzymes and the RING domain. (C) Purified Listerin was titrated into reactions containing purified 80S F-VHP-β-tRNA RNCs (F-V-β-tRNA), E1 and E2 enzymes, ubiquitin, and energy with or without splitting factors (split. fact.). Reactions were analyzed by SDS-PAGE and autoradiography (representative samples shown on right). Polyubiquitination (poly-Ub) chains containing four or more ubiquitins were quantified, and the ratio of ubiquitination obtained with splitting factors to the level obtained without splitting factors was plotted against Listerin concentration (left, log scale on x-axis). Compared to physiological concentrations of Listerin (green arrow, lanes 1 and 2), which is strongly dependent on splitting factors for ubiquitination, higher amounts of Listerin (red arrow, lanes 3 and 4) ubiquitinate substrates at comparable levels with or without splitting factors. However, ubiquitination in the absence of splitting factors is not as processive (compare lanes 3 and 4). (D) Experimental scheme to isolate and compare competency of 60S- versus 80S-RNCs for Listerin-mediated ubiquitination. (E) 60S- or 80S-RNCs isolated from a splitting reaction of purified F-V-β-tRNA RNCs were incubated with 75 nM E1, 250 nM E2, 6 nM rListerin, 10 µM tagged ubiquitin, and energy. Percent ubiquitination is indicated (% of total substrate in ubiquitinated species; nd is not detected). Note that 60S RNCs are preferred substrates for Listerin-mediated ubiquitination.

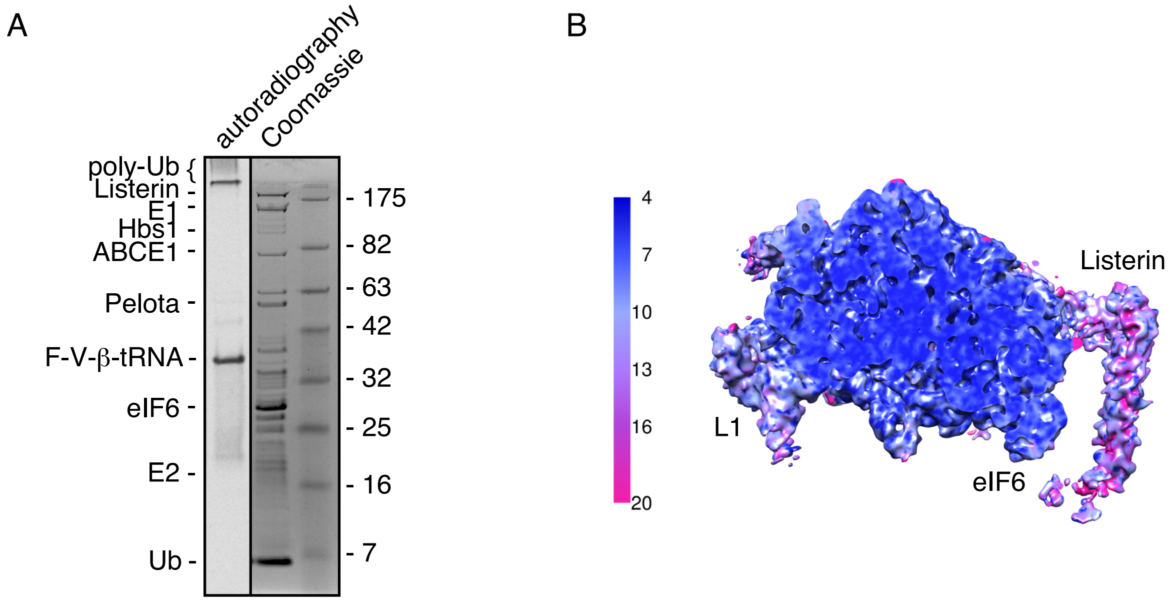


**Fig. S5** (Related to Fig. 6) (A) Ubiquitination reaction of purified $^{35}$S labeled 80S F-VHP-β-tRNA RNCs with all purified factors at levels comparable to those used for cryo-EM studies in Fig. 6a. Autoradiograph shows efficient nascent chain ubiquitination; Coomassie stained image of the same gel is also shown. (B) Local resolution map of 60S-Listerin reconstruction showing that the ribosome and eIF6 are much better resolved than the Listerin density.

Specific for tRNA

| ID | + RNase | - RNase |
|---|---|---|
| PAIRB | 3 | 75 |
| NEMF | 0 | 61 |
| LTN1 | 1 | 42 |
| TPP2 | 1 | 37 |
| SLFN14 | 1 | 24 |
| TTC37 | 0 | 31 |
| LARP1 | 0 | 23 |
| SKIV2 | 0 | 21 |
| ABCE1 | 0 | 17 |
| PABP | 0 | 17 |
| PFKL | 2 | 13 |
| PELO | 0 | 15 |
| DJC21 | 0 | 16 |
| NACA | 0 | 13 |
| YBOX1 | 0 | 13 |
| TCPG | 0 | 13 |
| FOCAD | 0 | 13 |
| DHX29 | 0 | 12 |
| HBS1L | 0 | 11 |
| DESP | 0 | 9 |
| CCT8 | 0 | 9 |
| SMBP2 | 0 | 10 |
| DNAJC13 | 0 | 9 |
| GROL | 0 | 8 |
| APOD | 0 | 8 |
| PSMD3 | 1 | 7 |
| PA2G4 | 1 | 7 |
| DHX9 | 0 | 8 |
| MARR | 0 | 7 |
| ZNF622 | 0 | 7 |
| SND1 | 0 | 7 |
| HS90B | 0 | 7 |
| CASP14 | 0 | 6 |
| CCT2 | 0 | 7 |
| C6orf86 | 0 | 5 |
| PSB5 | 1 | 4 |
| C1TC | 0 | 6 |
| ABCF1 | 0 | 5 |
| IFRD1 | 0 | 5 |
| CASB | 1 | 3 |
| TGM3 | 1 | 3 |
| LOX12 | 1 | 3 |
| TCPD | 0 | 4 |
| AGO2 | 0 | 4 |
| CLH1 | 0 | 3 |
| AP2A2 | 0 | 3 |
| G3P | 0 | 3 |
| ZC3HF | 0 | 4 |
| SMC2 | 0 | 3 |
| GEMIN5 | 0 | 3 |
| DSC1 | 0 | 3 |
| TCPA | 0 | 3 |

Not specific for tRNA

| ID | + RNase | - RNase |
|---|---|---|
| IGKC | 98 | 63 |
| IGH1M | 75 | 57 |
| SC61B | 47 | 31 |
| VIL1 | 24 | 14 |
| HBB | 12 | 22 |
| H13 | 32 | 0 |
| KRT6C | 14 | 17 |
| HSP7C | 13 | 18 |
| ERCC4 | 12 | 14 |
| TRY10 | 17 | 9 |
| PRSS1 | 12 | 6 |
| VCP | 6 | 12 |
| PYR1 | 8 | 8 |
| HBA | 5 | 6 |
| PSMD2 | 8 | 3 |
| RFET7 | 4 | 6 |
| DCD | 3 | 6 |
| BCEN2424 | 6 | 3 |
| PSMD1 | 4 | 5 |
| CASA2 | 7 | 1 |
| PRS4 | 5 | 3 |
| PRS7 | 3 | 5 |
| MALE | 5 | 2 |
| CASA1 | 4 | 2 |
| PSMD4 | 3 | 3 |
| PRS8 | 2 | 4 |
| PSD11 | 2 | 3 |
| LGB2 | 5 | 0 |
| PSB6 | 2 | 2 |
| THIO | 1 | 2 |
| RANBP10 | 2 | 1 |
| C27C1 | 1 | 2 |
| PRS10 | 3 | 0 |

Translation-related factors

| ID | + RNase | - RNase |
|---|---|---|
| EF2 | 7 | 146 |
| REF | 50 | 29 |
| EIF3A | 0 | 44 |
| EIF3C | 1 | 24 |
| EIF3B | 5 | 6 |
| IF4GI | 0 | 12 |
| EIF3E | 2 | 18 |
| IF2G | 4 | 5 |
| EIF3D | 1 | 7 |
| EIF6 | 0 | 7 |
| IF2B | 1 | 5 |
| EI2BD | 0 | 6 |
| EI2BE | 2 | 5 |
| IF2A | 0 | 4 |
| IF2P | 0 | 4 |
| IF4A1 | 0 | 3 |
| EIF3I | 0 | 3 |
| EIF3G | 1 | 0 |
| FARSB | 0 | 10 |
| LARS | 0 | 8 |
| DARS | 0 | 7 |
| IARS | 0 | 6 |
| EPRS | 0 | 7 |
| FARSA | 0 | 3 |
| QARS | 1 | 1 |

60S proteins

| ID | + RNase | - RNase |
|---|---|---|
| RPL3 | 0 | 166 |
| RPL5 | 0 | 107 |
| RPL7A | 0 | 89 |
| RPLP0 | 1 | 81 |
| RPL18 | 12 | 71 |
| RPL4 | 0 | 70 |
| RPL7 | 3 | 87 |
| RPL13 | 3 | 61 |
| RPL24 | 3 | 56 |
| RPL6 | 0 | 58 |
| RPL11 | 6 | 39 |
| RPL13A | 4 | 39 |
| RPL21 | 0 | 41 |
| RPL8 | 1 | 37 |
| RPL23A | 4 | 33 |
| RPL10 | 0 | 36 |
| RPL30P | 2 | 33 |
| RPL12 | 3 | 32 |
| RPL19 | 0 | 34 |
| RPL30 | 2 | 38 |
| RPL17 | 2 | 30 |
| RPL23 | 2 | 30 |
| RPL27A | 2 | 28 |
| RPL26 | 1 | 30 |
| RPL14 | 0 | 31 |
| RPL27 | 1 | 28 |
| RPL35A | 1 | 28 |
| RPL18A | 0 | 28 |
| RPL31 | 2 | 26 |
| RPL15 | 1 | 26 |
| RPL28 | 2 | 26 |
| RPLP2 | 0 | 26 |
| RPL38 | 1 | 25 |
| PL34 | 1 | 22 |
| RPS7 | 2 | 20 |
| RPL36A | 1 | 21 |
| RPL36 | 2 | 18 |
| RPL22 | 1 | 17 |
| RPL9 | 0 | 16 |
| RPL35 | 1 | 15 |
| RPL37A | 0 | 17 |
| RPL10A | 0 | 13 |
| RPL30.1 | 0 | 10 |
| RPL32 | 0 | 11 |
| RPL22L | 0 | 9 |
| RPLS2 | 0 | 8 |
| RPLA1 | 0 | 4 |
| RPL37 | 0 | 3 |

40S proteins

| ID | + RNase | - RNase |
|---|---|---|
| RPS2 | 3 | 125 |
| RPS3 | 6 | 124 |
| GBLP | 2 | 93 |
| RPS3A | 7 | 71 |
| RPS4X | 2 | 69 |
| RPS27A | 23 | 40 |
| RPS6 | 0 | 60 |
| RPS18 | 8 | 51 |
| RPS8 | 0 | 55 |
| RPS9 | 5 | 43 |
| RPSA | 3 | 42 |
| RPS16 | 1 | 44 |
| RPS19 | 5 | 37 |
| RPS27L | 3 | 38 |
| RPS15A | 3 | 35 |
| RPS21 | 2 | 53 |
| RPS23 | 0 | 29 |
| RPS14 | 2 | 26 |
| RPS5 | 2 | 29 |
| RPS12 | 2 | 29 |
| RPS25 | 1 | 23 |
| RPS24 | 0 | 32 |
| RPS20 | 0 | 20 |
| RPS11 | 0 | 18 |
| RPS17 | 0 | 19 |
| RPS29 | 2 | 14 |
| RPS13 | 3 | 16 |
| RPS28 | 1 | 13 |
| RPS30 | 0 | 14 |
| RPS26.2 | 0 | 13 |
| RPS27 | 0 | 8 |
| RPS10 | 0 | 7 |
| RSP17 | 0 | 6 |

**Table S1 (Related to Fig. 4) Mass spectrometry of the native mammalian RQC complex.**
F-VHP-β RNCs produced by in vitro translation were purified to isolate native RQC complexes with and without RNase A treatment as in Fig. S3b. Gel slices from the purified samples (Fig. 4b) were analyzed by mass spectrometry. All identified proteins with more than 3 spectral counts are listed. The F-VHP-β substrate and components thought to be involved in the RQC pathway from earlier work are highlighted in yellow and green, respectively.

**Supplemental Experimental Procedures**

### *Plasmids and antibodies*

The constructs encoding untagged VHP-β and β, WT and dominant negative Hbs1, Pelota, and eIF6 have been described (Shao et al., 2013). For isolation of 80S RNCs, the open reading frames of VHP-β and β were cloned into an SP64 vector containing an N-terminal 3X tandem Flag tag using standard protocols. The human open reading frames of Listerin and ABCE1 were cloned into a pcDNA-based vector encoding an N-terminal 3X tandem Flag tag using standard procedures. ΔRING Listerin was generated using Phusion mutagenesis. Anti-Listerin (Abcam), anti-Flag (Sigma), anti-RPL9 (Santa Cruz), and anti-RPS16 (Santa Cruz) antibodies have been described (Shao et al., 2013). Rabbit polyclonal antibodies against Hbs1 and ABCE1 were generated with KLH-conjugated peptide antigens [Hbs1-ARHRNVRGYNYDEDFE, ABCE1-IKDVEQKKSGNYFFLDD] (Cambridge Research Biomedicals). Anti-Flag resin and 3X Flag peptide used for affinity purification were obtained from Sigma.

### *In vitro transcription and translation*

All transcripts were generated using PCR products. The 5' primer anneals just before the SP6 promoter in the SP64 vector. The 3' primer to generate F-VHP-β anneals to residues 63-68 of the Sec61β sequence and appends an additional valine residue to generate a stable valyl-tRNA (see Fig. 1a). The 3' primer to generate F-β truncates after residue 40 of the Sec61β open reading frame and appends the residues "MLKV" to generate stable RNCs (Fig. S1a). Transcription reaction were carried out with PCR templates and SP6 polymerase for 1 hour at 37°C. The transcripts were used directly for translation in an in vitro rabbit reticulocyte (RRL) system as previously described (Shao et al., 2013; Sharma et al., 2010).

### *Purification of salt-washed 80S RNCs*

A translation reaction of F-VHP-β or F-β was assembled and incubated at 32°C. After 7 minutes, an excess (~50 nM) of dominant negative Hbs1 (Hbs1-DN) was added and the reaction was allowed to proceed for an additional 18 minutes (see diagram, Fig. 1a). Translation reactions were placed on ice and adjusted to a final concentration of 750 mM KAc and 15 mM $MgAc_2$. 1 mL of salt-adjusted translation reaction was layered on a 1.6 mL high salt sucrose cushion (50 mM Hepes, pH 7.4, 750 mM KAc, 15 mM $MgAc_2$, 0.5 M sucrose) and spun for 1 hour at 100,000 rpm in a TLA100.3 rotor in a Beckman Optima-Max ultracentrifuge. For isolation of RNCs under physiological conditions (Fig. 1c), no adjustments were made to the salt concentrations and ribosomes were isolated from translation reactions through a 0.5 M sucrose cushion in physiological salt (50 mM Hepes, pH 7.4, 100 mM KAc, 2.5 mM $MgAc_2$). The ribosome pellet was resuspended in RNC buffer (50 mM Hepes, 100 mM KAc, 5 mM $MgAc_2$, 1 mM DTT) and incubated with anti-Flag resin for 1-1.5 hour at 4°C. The resin was washed three times with RNC buffer with 0.1% Triton X-100, three times with 50 mM Hepes, 250 mM KAc, 5 mM $MgAc_2$, 0.5% Triton X-100, 1 mM DTT, and three times with RNC buffer. Bound RNCs were eluted with 0.1 mg/ml 3X Flag peptide in RNC buffer. Two elutions of one column volume each were conducted for 30 minutes at room temperature and combined.

### *Preparation of S-100 and ribosome salt wash*

Rabbit reticulocyte lysate (RRL) S-100 was prepared by spinning crude RRL in a TLA100.3 rotor at 100,000 rpm for 40 minutes. The supernatant was used directly for downstream

assays (Fig. 1e). The pellet containing crude ribosomes was resuspended into ribosome wash buffer (20 mM Hepes, pH 7.4, 100 mM KAc, 1.5 mM $MgAc_2$, 0.1 mM EDTA, 1 mM DTT) and re-pelleted over a 0.5 M sucrose cushion in ribosome wash buffer. The ribosome pellet ("native ribosomes") was resuspended in 20 mM Hepes, pH 7.4, 200 mM KAc, 1.6 mM $MgAc_2$, 10% glycerol, 1 mM DTT and stored at -80°C. To generate a ribosome salt wash (Fig. 2a), native ribosomes were adjusted to a final concentration of 750 mM KAc, 15 mM $MgAc_2$. 1 mL of the ribosomes was layered over a 1.6 mL high salt 0.5 M sucrose cushion and centrifuged in a TLA100.3 rotor at 100,000 rpm for 1 hour. The supernatant was saved and either dialyzed against RNC buffer containing 10% glycerol for functional assays or desalted over a PD-10 column (GE Healthcare) equilibrated in RNC buffer for further fractionation steps (Fig. S2b). To generate a ribosome salt wash from tissue culture cells (Fig. S2a), HEK293T cells were lysed in 50 mM Hepes, pH 7.4, 100 mM KAc, 2.5 mM $MgAc_2$, 0.5% Triton X-100, 1 mM DTT, 1X protease inhibitor cocktail (Roche) and spun in a tabletop centrifuge for 10 minutes at 4°C. Ribosomes and the accompanying ribosome salt wash from the post-nuclear supernatant were then isolated exactly according to the procedure for reticulocyte lysate above.

### *Fractionation of ribosome salt wash*

Desalted total ribosome salt wash was passed over a DEAE-sepharose column equilibrated in RNC buffer (Fig. S2b). The flow-through (fraction 1) was collected, and the column was washed extensively with RNC buffer. The first step elution (fraction 2) was with 50 mM Hepes, 250 mM KAc, 5 mM $MgAc_2$, 1 mM DTT. The column was washed three times with the first elution buffer before the second step elution (fraction 3) with 50 mM Hepes, pH 7.4, 750 mM KAc, 5 mM $MgAc_2$, 1 mM DTT. For functional assays, all elutions were dialyzed against RNC buffer containing 10% glycerol. Fraction 2 after the DEAE step purification was further fractionated over an S-sepharose column equilibrated in RNC buffer (Fig. S3a). The flow-through and elution with 750 mM KAc were collected and analyzed for splitting-dependent ubiquitination activity. The elution was then fractionated over a 5-25% sucrose gradient in RNC buffer in a TLS-55 rotor at 55,000 rpm for 5 hours 4°C with the slowest acceleration and deceleration settings. Eleven 200 μL fractions were collected from the top and analyzed for activity (Fig. 4a). Fractions 2-7 were then processed for mass spectrometry analysis (Fig. 4d).

### *Affinity purification of native nascent chain-tRNA RQC complexes*

An 8 ml translation reaction of F-VHP-β (without Hbs1-DN) was incubated for 30 minutes at 32°C to permit generation of native RQC complexes (see Fig. 1a). 1 ml aliquots were layered over 1.6 mL sucrose cushions containing 0.5 M sucrose in physiological salt (50 mM Hepes, pH 7.4, 100 mM KAc, 2.5 mM $MgAc_2$, 1 mM DTT) and spun in a TLA110 rotor at 100,000 rpm for 1 hour at 4°C. The ribosomal pellets were resuspended in a total of 800 μL of RNC buffer. 200 μL was loaded onto each of four 4.8 mL 10-30% sucrose gradients and spun in a MLS-50 rotor at 50,000 rpm for 2 hours at 4°C with the slowest acceleration and deceleration settings. Twenty-five 200 μL fractions were collected from the top. Based on previous characterizations of this gradient, fractions 14-17, which contain the majority of 60S ribosomal subunits (Shao et al., 2013), were pooled for downstream purification. The pooled fractions were divided into two halves. One half was treated with 50 μg/mL RNase A at room temperature for 15 min to generate a negative control of non-tRNA specific interacting proteins. Both samples (with and without RNase treatment) were incubated with 50 μL of packed anti-Flag resin at 4°C for 1 hour. The resin was washed three times in RNC buffer with 0.1% Triton X-100, three times with 50 mM Hepes, pH 7.4, 250 mM KAc, 5 mM

$MgAc_2$, 0.5% Triton X-100, 1 mM DTT, and then another three times with RNC buffer with 0.1% Triton X-100. Elutions were carried out with 0.15 mg/mL 3X Flag peptide in RNC buffer with 0.1% Triton X-100 for 30 minutes at room temperature. Two sequential elutions were combined and subject to TCA precipitation and subsequent analysis by SDS-PAGE (Fig. 4b) and mass spectrometry (Table S1).

***Ubiquitination and ribosome subunit splitting assays***

For most functional assays, F-VHP-β RNCs at 5 nM was incubated with S-100 (Fig. 1e), ribosome salt wash, or ribosome salt wash fractions (Fig. 2b-2d, S2a, and S2c) at ratios normalized to their levels in total lysate based on the fractionation procedure. With purified components, increasing the concentration of RNCs to ~60-100 nM had little qualitative effect on the results (Fig. S5a). Recombinant splitting factors (Fig. 2a) were added to a final concentration of either 50 nM or to equimolar levels as the RNCs in the reaction (Fig. 2c, 2d, 5b, 5c, and S5a). Recombinant Listerin was added at a range of 0.3 nM ("physiological" concentration by comparison to fraction 2) and titrated up to 120 nM (Fig. S4c). Unless otherwise indicated, all reactions contained 75 nM E1, 250 nM of E2 (UbcH5a), and 10 μM of His- or HA-tagged ubiquitin (all from Boston Biochem). Where applicable (Fig. 1b, 4c, and 5c), reactions were treated with 50 μg/mL of RNase A for 10 minutes at room temperature before the addition of ubiquitination reagents. Reactions were conducted at 32°C for 5-20 minutes, and either analyzed directly by SDS-PAGE and autoradiography, subject to additional centrifugation steps, or rapidly denatured for ubiquitin pulldowns as indicated in individual figure legends. To monitor ribosome subunit splitting (Fig. 2c), isolated 80S F-β RNCs were incubated with the desired components at the same concentrations and conditions as for ubiquitination reactions. The reactions were then centrifuged in a TLA120.1 rotor at 70,000 rpm for 30 minutes at 4°C. The supernatant was saved and the pellet was resuspended in the original reaction volume's worth of 1% SDS. Equal amounts of the total, supernatant, and pellet fractions were loaded for SDS-PAGE analysis and autoradiography.

***Purification of recombinant proteins***

His-tagged eIF6 and Pelota were purified via Ni-NTA using standard procedures. Briefly, the constructs were transformed into BL21(DE3) cells. Cells were grown under antibiotic selection, induced overnight at 16°C with 0.2 mM IPTG, and harvested and lysed with a microfluidizer in lysis buffer (1X PBS, 250 mM NaCl, 10 mM imidazole, 1X protease inhibitor cocktail, 1 mM DTT). Lysates were clarified by centrifugation, bound to a Ni-NTA column by gravity flow, and washed extensively with lysis buffer. Elution was carried out in lysis buffer containing 250 mM imidazole. Peak fractions were pooled and dialyzed into RNC buffer with 10% glycerol. Flag-tagged Hbs1, Hbs1-DN, and ABCE1 were transfected into HEK293T cells using TransIT 293 (Mirus). Cells were split 1:4 the day after transfection and purified two days later. Generally, eight 10 cm dishes of confluent cells were harvested in ice cold PBS and lysed in a total volume of 1.5 mL of lysis buffer (50 mM Hepes, pH 7.4, 100 mM KAc, 5 mM $MgAc_2$, 0.5% Triton X-100, 1 mM DTT, 1X protease inhibitor cocktail). Lysates were spun for 10 minutes in a tabletop centrifuge at 4°C and the post-nuclear supernatant was incubated with 100 μL of packed anti-Flag resin for 1-1.5 hour at 4°C. The resin was washed three times with lysis buffer, three times with 50 mM Hepes, pH 7.4, 400 mM KAc, 5 mM $MgAc_2$, 0.5% Triton X-100, 1 mM DTT, and three times with RNC buffer. Elutions were carried out with one column volume of 0.1 mg/mL 3X Flag peptide in RNC buffer at room temperature for 30 minutes. Two sequential elutions were combined for downstream assays. Flag-tagged WT and ΔRING Listerin (Fig. 5) were transfected into HEK293T cells using TransIT 293 (Mirus), split 1:4 the day after transfection, and purified

two days after. Sixteen 10 cm dishes of confluent Listerin-expressing cells were harvested in ice cold PBS and lysed in ~1 mL 25 mM Hepes, pH 7.4, 125 mM KAc, 15 mM $MgAc_2$, 1:20,000 dilution of RNasin (Promega), 50 μg/mL cycloheximide, 100 μg/mL digitonin, 1 mM DTT, 1X protease inhibitor cocktail (Roche), rotating at 4°C for 10 minutes. The lysates were then spun in a tabletop centrifuge at 4°C for 10 minutes. The post-nuclear supernatant was centrifuged in a TLA100.3 rotor at 100,000 rpm for 40 minutes at 4°C. The S-100 was incubated with 100 μL of packed anti-Flag resin for 1-1.5 hour at 4°C. The resin was washed three times in the buffer used to lyse the cells, three times in 25 mM Hepes, pH 7.4, 400 mM KAc, 5 mM $MgAc_2$, 100 μg/mL digitonin, 1 mM DTT, and three times in 25 mM Hepes, pH 7.4, 250 mM KAc, 5 mM $MgAc_2$, 10% glycerol, 50 μg/mL digitonin, 1 mM DTT. Elutions were carried out with one column volume of 0.1 mg/mL 3X Flag peptide in the final wash buffer at room temperature for 30 minutes. Two sequential elutions were combined for downstream assays.

***Miscellaneous biochemistry***

Samples for ubiquitin pulldowns were denatured by boiling in 1% SDS, 0.1 M Tris, pH 8. His-ubiquitin pulldowns were performed with Ni-NTA in 1X PBS, 250 mM NaCl, 0.5% Triton X-100, 10 mM imidazole. HA-ubiquitin and Flag-ubiquitin pulldowns were performed in 1X PBS, 250 mM NaCl, 0.5% Triton X-100 with either anti-Flag resin or polyclonal anti-HA antibody with Protein A agarose beads. All samples were incubated with resin for 1-2 hours rotating at 4°C, washed twice in the pulldown buffer and eluted with protein sample buffer for SDS-PAGE analysis. 10 mM EDTA was included in the elution of the pulldowns with Ni-NTA to chelate the nickel. TCA precipitations were carried out at 4°C for 10 minutes with 15% TCA and 0.5% Triton X-100. Proteins were washed once with cold acetone, air dried, and solubilized directly protein sample buffer for SDS-PAGE analysis. SDS-PAGE was performed with 7.5% or 10% Tris-tricine gels. For immunoblots, gels were transferred to 0.2 μm nitrocellulose membrane (Bio-Rad) and blocked in 5% milk in PBS with 0.1% Tween-20. Primary antibodies were incubated at the following concentrations: 1:500 anti-Listerin, 1:4000 anti-Hbs1, 1:4000 anti-ABCE1, 1:100 anti-RPL9, 1:100 anti-RPS16, 1:500 anti-TCF25, 1:5000 anti-Flag. For autoradiography, gels were fixed and stained in 0.1% Coomassie R250 in 40% ethanol, 10% acetic acid for at least 20 minutes. After destaining in 10% ethanol, 7.5% acetic acid, gels were dried and exposed to MR film (Kodak). For mass spectrometry, SDS-PAGE gels were stained with colloidal Coomassie stain (Pierce) and destained in water. For gradient fractions, gels were run approximately only 1 inch into the separating gel. ~1 mm gel slices were excised from each lane and analyzed for protein identifications by in-house mass spectrometry facilities.

***Cryo-electron microscopy and image analysis***

Reactions containing approximately 120 nM F-VHP-β RNCs, equimolar amounts of splitting factors, and approximately 10-fold excess of Listerin and eIF6 (Fig. 6a) were incubated for 15 minutes at 32°C and immediately frozen on grids. Samples were applied to glow-discharged R2/2 Cu 400 mesh grids (Quantifoil) coated with a thin (~50 Å) layer of continuous carbon and vitrified with an FEI Vitrobot. Grids were screened on a Tecnai T12. Several datasets collected on a Polara allowed for sample optimization, mainly to increase the amount of eIF6 and Listerin in the reaction to favor stable 60S subunits and complex formation. Overnight automated data collection (EPU software, FEI) was conducted on a Titan Krios operated at 300 kV, at 104,478X magnification. One second exposures yielding a total dose of 35 electrons/$Å^2$ were collected with defocus values ranging from 2 to 3.5 μm. Particles were picked with e2boxer (EMAN2) (Tang et al., 2007), yielding an initial dataset

of 172,267 particles, which was processed through Relion (Scheres, 2012). After 2D classification, 154,257 particles underwent 3D classification. 80S and 60S ribosome classes, consisting of 70,883 and 40,265 particles, respectively, were combined and refined separately. The 3D refinements were then used to reclassify the corresponding datasets. For the 80S ribosomes, this yielded classes of 80S ribosomes with P and E-site tRNA density (44,255 particles) and of empty ribosomes with and without eEF2 bound. The 80S ribosome with tRNA density was then individually refined, corrected for beam-induced movement and radiation damage by movie frame processing (Bai et al., 2013), and further refined. This resulted in a final resolution, according to gold-standard FSC criteria, of 3.8 Å for the 80S with P and E-site tRNA density. For the 60S ribosomes, the first 3D classification yielded a class of 26,925 particles with a weak additional density that corresponded to Listerin. This class was further refined and used for another round of 3D classification. Listerin-containing particles from this classification step (15,188) were refined and corrected for movement and radiation damage. This refined model was used for another round of classification with a mask for the Listerin density, yielding 9,148 particles with clear Listerin density (Fig. 6b and 6c). Refinement of this class yielded a final structure with a resolution of 4.9 Å, although the local resolution for Listerin itself ranges from 5 to more than 15 Å (Fig. S5b). After the first round of 3D classification, classes of empty 60S subunits (11,710 particles) were also combined and refined to a final resolution of 4.4 Å. For comparisons in the displayed figures, all structures displayed were low pass filtered to 8 Å. Listerin difference density was fit into "snapshot V" of negative stain reconstructions of yeast Ltn1 (emd-2252, Fig. 6d) (Lyumkis et al., 2013). Rigid body fitting of eIF6-bound 60S (Fig. 6e) was performed with the crystal structure of *T. thermophila* 60S ribosome subunit in complex with eIF6 (molecule 2, PDB 4A17 and 4A19) (Klinge et al., 2011). Figures were visualized, analyzed, and generated using the UCSF Chimera package (Pettersen et al., 2004).

## Supplemental References

Bai, X.-C., Fernandez, I.S., McMullan, G., and Scheres, S.H. (2013). Ribosome structures to near-atomic resolution from thirty thousand cryo-EM particles. Elife *2*, e00461–e00461.

Klinge, S., Voigts-Hoffmann, F., Leibundgut, M., Arpagaus, S., and Ban, N. (2011). Crystal structure of the eukaryotic 60S ribosomal subunit in complex with initiation factor 6. Science *334*, 941–948.
